# Supplementary material for: Effect of midwife-led pelvic floor muscle training on prolapse symptoms and quality of life in women with pelvic organ prolapse in Ethiopia: A Cluster-randomized controlled trial
Source: PLoS Med. 2025 Mar 31;22(3):e1004468. doi: 10.1371/journal.pmed.1004468 (PMC11977982; doi:10.1371/journal.pmed.1004468)
Supplement: S1 File — (DOCX) [file pmed.1004468.s001.docx]

CONSORT 2010 checklist of information to include when reporting a Randomized trial

| **Section/Topic** | **Item No** | **Checklist item** | **Reported on page No**** |
| --- | --- | --- | --- |
| **Title and abstract** | 1a | Identification as a randomized trial in the title | Title page 1 |
|  | 1b | Structured summary of trial design, methods, results, and conclusions _(for_ _specific_ _guidance_ _see_ _CONSORT_ _for_ _abstracts)_ | Abstract section 2 |
| **Introduction**  Background and | 2a | Scientific background and explanation of rationale | Introduction paragraph 3-6 |
| objectives | 2b | Specific objectives or hypotheses | Introduction paragraph 7 |
| **Methods**  Trial design | 3a | Description of trial design (such as parallel, factorial) including allocation ratio | Study design paragraph 1 |
|  | 3b | Important changes to methods after trial commencement (such as eligibility criteria), with reasons | N/A |
| Participants | 4a | Eligibility criteria for participants | Study design paragraph 1 |
|  | 4b | Settings and locations where the data were collected | Outcome paragraph 2-5 |
| Interventions | 5 | The interventions for each group with sufficient details to allow replication, including how and when they were | Procedure paragraph 2-13 |
|  |  | actually administered |  |
| Outcomes | 6a | Completely defined pre-specified primary and secondary outcome measures, including how and when they | Outcome paragraph 1 and 5 |
|  |  | were assessed |  |
|  | 6b | Any changes to trial outcomes after the trial commenced, with reasons | N/A |
| Sample size | 7a | How sample size was determined | Study design paragraph 2-4 |
|  | 7b | When applicable, explanation of any interim analyses and stopping guidelines | N/A |
| Randomisation: |  |  |  |
| Sequence | 8a | Method used to generate the random allocation sequence | Randomization paragraph 1 |
| generation | 8b | Type of randomisation; details of any restriction (such as blocking and block size) | Randomization paragraph 2 |
| Allocation | 9 | Mechanism used to implement the random allocation sequence (such as sequentially numbered containers), | Randomization paragraph 2 |
| concealment |  | describing any steps taken to conceal the sequence until interventions were assigned |  |
| mechanism |  |  |  |
| Implementation | 10 | Who generated the random allocation sequence, who enrolled participants, and who assigned participants to | Randomization paragraph 3 |
|  |  | interventions |  |
| Blinding | 11a | If done, who was blinded after assignment to interventions (for example, participants, care providers, those | Randomization paragraph 4 |

|  |  | assessing outcomes) and how |  |
| --- | --- | --- | --- |
|  | 11b | If relevant, description of the similarity of interventions | Procedure paragraph 10-13 |
| Statistical methods | 12a | Statistical methods used to compare groups for primary and secondary outcomes | Statistical analysis paragraph 1-5 |
|  | 12b | Methods for additional analyses, such as subgroup analyses and adjusted analyses | Statistical analysis paragraph 4 |
| **Results**  Participant flow (a | 13a | For each group, the numbers of participants who were randomly assigned, received intended treatment, and |  |
| diagram is strongly |  | were analysed for the primary outcome | Figure 1 |
| recommended) | 13b | For each group, losses and exclusions after randomisation, together with reasons | Figure 1 |
| Recruitment | 14a | Dates defining the periods of recruitment and follow-up | Randomization paragraph 3 |
|  | 14b | Why the trial ended or was stopped | Procedure paragraph 8 |
| Baseline data | 15 | A table showing baseline demographic and clinical characteristics for each group | Table 1 |
| Numbers analysed | 16 | For each group, number of participants (denominator) included in each analysis and whether the analysis was |  |
|  |  | by original assigned groups | Tables 2-4 |
| Outcomes and | 17a | For each primary and secondary outcome, results for each group, and the estimated effect size and its |  |
| estimation |  | precision (such as 95% confidence interval) | Tables 3-4 |
|  | 17b | For binary outcomes, presentation of both absolute and relative effect sizes is recommended | N/A |
| Ancillary analyses | 18 | Results of any other analyses performed, including subgroup analyses and adjusted analyses, distinguishing | Table 4 |
|  |  | pre-specified from exploratory |  |
| Harms | 19 | All important harms or unintended effects in each group _(for_ _specific_ _guidance_ _see_ _CONSORT_ _for_ _harms)_ | Adverse events paragraph 1 |
| **Discussion**  Limitations | 20 | Trial limitations, addressing sources of potential bias, imprecision, and, if relevant, multiplicity of analyses | Discussion paragraph 7 |
| Generalisability | 21 | Generalisability (external validity, applicability) of the trial findings | Discussion paragraph 6 |
| Interpretation | 22 | Interpretation consistent with results, balancing benefits and harms, and considering other relevant evidence | Discussion paragraph 2-5 |
| **Other information**  Registration | 23 | Registration number and name of trial registry | Methodology section last paragraph |
| Protocol | 24 | Where the full trial protocol can be accessed, if available | Supplementary file 3 |
| Funding | 25 | Sources of funding and other support (such as supply of drugs), role of funders | Declaration paragraph 5 |

*We strongly recommend reading this statement in conjunction with the CONSORT 2010 Explanation and Elaboration for important clarifications on all the items. If relevant, we also recommend reading CONSORT extensions for cluster randomised trials, non-inferiority and equivalence trials, non-pharmacological treatments, herbal interventions, and pragmatic trials. Additional extensions are forthcoming: for those and for up to date references relevant to this checklist, see [www.consort-statement.org.](http://www.consort-statement.org/)
