# Supplementary material for: Effect of midwife-led pelvic floor muscle training on prolapse symptoms and quality of life in women with pelvic organ prolapse in Ethiopia: A Cluster-randomized controlled trial
Source: PLoS Med. 2025 Mar 31;22(3):e1004468. doi: 10.1371/journal.pmed.1004468 (PMC11977982; doi:10.1371/journal.pmed.1004468)
Supplement: S3 File — (DOCX) [file pmed.1004468.s003.docx]

**HAWASSA UNIVERSITY**

**COLLEGE OF MEDICINE AND HEALTH SCIENCES**

**SCHOOL OF PUBLIC HEALTH**

**EPIDEMIOLOGY OF PELVIC ORGAN PROLAPSE IN SIDAMA NATIONAL REGIONAL STATE, ETHIOPIA**

**A PhD DESSERTATION PROPOSAL**

**MELESE SIYOUM (MSc)**

**ADVISOR: AYALEW ASTATKIE (PhD, ASSOCIATE PROFESSOR)**

**CO-ADVISORS: WONDWOSEN T/SELASSIE (PhD, ASSISTANT PROFESSOR)**

**RAHEL NARDOS (OBSTETRICIAN AND GYNECOLOGIST, ASSOCIATE PROFESSOR)**

**CO-AUTHOR: BINIYAM SIRAK (OBESTETRICIAN AND GYNECOLOGIST)**

**APRIL, 2022**

**HAWASSA, ETHIOPIA**

**EPIDEMIOLOGY OF PELVIC ORGAN PROLAPSE IN SIDAMA NATIONAL REGIONAL STATE, ETHIOPIA**

**MELESE SIYOUM (MSc)**

**ADVISORS: AYALEW ASTATKIE (PhD, ASSOCIATE PROFESSOR)**

**CO-ADVISORS: WONDWOSEN T/SELASSIE (PhD, ASSISTANT PROFESSOR)**

**RAHEL NARDOS (OBSTETRICIAN AND GYNECOLOGIST, ASSOCIATE PROFESSOR)**

**CO-AUTHOR: BINIYAM SIRAK (OBESTETRICIAN AND GYNECOLOGIST)**

**A DISSERTATION PROPOSAL SUBMITTED TO THE SCHOOL OF PUBLIC HEALTH, COLLEGE OF MEDICINE AND HEALTH SCIENCES, HAWASSA UNIVERSITY IN PARTIAL FULFILMENT OF THE REQUIREMENTS FOR THE DEGREE OF DOCTOR OF PHILOSOPHY IN PUBLIC HEALTH.**

**APRIL, 2022**

**HAWASSA, ETHIOPIA**

# **REVIEWERS’ APPROVAL SHEET**

This is to confirm that we have reviewed the proposal entitled ‘**Epidemiology of Pelvic Organ Prolapse in Sidama National Regional State, Ethiopia**’ submitted in partial fulfilment of the requirements for the degree of Doctor of Philosophy in Public Health, and carried out by Melese Siyoum, ID.No. PhDPuHeR/0004/13.

Therefore we recommend that the student has addressed the comments provided and fulfilled the requirements and hence hereby can submit the proposal to the school.

Name of Reviewer Signature Date

1. ___________________ ____________ _____________
2. ___________________ ____________ _____________

# **ADVISORS’ APPROVAL SHEET**

I hereby declare that this PhD dissertation Proposal is my original work and has not been presented for a degree in any other university, and all sources of material used for this thesis / dissertation have been duly acknowledged.
Name: ___________________________________
Signature: _______________________

This is to certify that proposal entitled ‘**Epidemiology of Pelvic Organ Prolapse in Sidama National Regional State, Ethiopia**’ submitted in partial fulfilment of the requirements for the degree of Doctor of Philosophy in Public Health, the post graduate programme of School of Public Health, and carried out by Melese Siyoum, ID.No. PhDPuHeRe/0004/13, under our supervision.

Therefore we recommend that the student has fulfilled the requirements and hence hereby can submit the proposal to the school.

Name of major advisor Signature Date

___________________ ____________ _____________

Name of co-advisor Signature Date

___________________ ____________ _____________

# **ACKNOWLEDGEMENTS**

I would want to express my gratitude to Hawassa University for allowing me to participate in this program. Also, I would like to thank my advisors, Ayalew Astatkie (PhD, Associate Professor) and Wondwosen T/Selasie (PhD, Assistant Professor), for their helpful remarks and direction in identifying the target area and reviewing the literature to better grasp this study issue. I would want to thank the personnel at the Midwifery Department for their patience and assistance when I first started this program. Dr. Rahel Nardos, Dr. Biniyam Sirak, and my friends Mr. Shewangizaw Mekonnen, Bedilu Deribe, Rekiku Fikre, and Zelalem Tenaw provided me with both formal and informal support. Finally, I would like to express my gratitude to Tigist Bekele, my wife, for taking on all of my obligations and leading my family.

**CONTENTS**

[**ADVISORS’ APPROVAL SHEET** iii](#_Toc100153457)

[**ACKNOWLEDGEMENTS** iv](#_Toc100153458)

[**LIST OF TABLES** viii](#_Toc100153459)

[**LIST OF FIGURES** ix](#_Toc100153460)

[**ABBREVIATIONS** x](#_Toc100153461)

[**SUMMARY** xi](#_Toc100153462)

[**1.** **INTRODUCTION** 1](#_Toc100153463)

[1.1. Background 1](#_Toc100153464)

[1.2. Statement of the problem 3](#_Toc100153465)

[**2.** **LITERATURE REVIEW** 6](#_Toc100153466)

[2.1. Prevalence of Pelvic Organ Prolapse 6](#_Toc100153467)

[2.2. Risk Factors for Pelvic Organ Prolapse 7](#_Toc100153468)

[2.3. Conceptual Framework 12](#_Toc100153469)

[2.4. Impact of POP on Health-Related Quality of Life 13](#_Toc100153470)

[2.5. Interventions against POP 13](#_Toc100153471)

[2.5.1. Conservative Management 13](#_Toc100153472)

[2.5.2. Surgical management of POP 15](#_Toc100153473)

[2.6. Justification of the Proposed Study 16](#_Toc100153474)

[2.7. Significance of the Study 16](#_Toc100153475)

[**3.** **OBJECTIVES** 19](#_Toc100153476)

[**3.1 General Objective** 19](#_Toc100153477)

[**3.2. Specific Objectives** 19](#_Toc100153478)

[**4.** **METHODS** 20](#_Toc100153479)

[**PHASE-I PROJECT** 20](#_Toc100153480)

[**PAPER-I** 20](#_Toc100153481)

[Objective 20](#_Toc100153482)

[The Original POP-SS questionnaire 20](#_Toc100153483)

[Translation and Cultural Adaptation of POP-SS 21](#_Toc100153484)

[Study Participants 22](#_Toc100153485)

[Sample Size 22](#_Toc100153486)

[Sampling Procedure 22](#_Toc100153487)

[Statistical Analysis 22](#_Toc100153488)

[**5.** **PAPER II** 25](#_Toc100153489)

[Specific Objectives 25](#_Toc100153490)

[Study Setting and Period 25](#_Toc100153491)

[Study Design 26](#_Toc100153492)

[Study Population 26](#_Toc100153493)

[Sample Size Determination 27](#_Toc100153494)

[Sampling Techniques 28](#_Toc100153495)

[Data Collection Tools and Procedures 30](#_Toc100153496)

[Study Variables 33](#_Toc100153497)

[Data Source and Measurement 33](#_Toc100153498)

[Data Quality Assurance 35](#_Toc100153499)

[Data Management and Analysis 36](#_Toc100153500)

[**PHASE-II PROJECT** 38](#_Toc100153501)

[**6.** **PAPER-III** 38](#_Toc100153502)

[Objective 38](#_Toc100153503)

[Study Area and Period 38](#_Toc100153504)

[Study Design 38](#_Toc100153505)

[Study Subjects 38](#_Toc100153506)

[Eligibility Criteria 38](#_Toc100153507)

[Sample Size Determination 39](#_Toc100153508)

[Randomization 39](#_Toc100153509)

[Description of Intervention 41](#_Toc100153510)

[Variables 42](#_Toc100153511)

[Data Measurements 42](#_Toc100153512)

[Data Collection Tools and Procedure 43](#_Toc100153513)

[Data Quality Control 43](#_Toc100153514)

[Data Management and Analysis 44](#_Toc100153515)

[**7.** **PAPER-IV** 46](#_Toc100153516)

[Objectives 46](#_Toc100153517)

[Study Area and Period 46](#_Toc100153518)

[Study Design, Study Subjects, Randomization, Sampling Technique and Description of Intervention 46](#_Toc100153519)

[Sample Size Determination 46](#_Toc100153520)

[Data Collection Tools and Procedures 47](#_Toc100153521)

[Variables 48](#_Toc100153522)

[Data Measurements 48](#_Toc100153523)

[Quality Assurance 49](#_Toc100153524)

[Data Management and Analysis 49](#_Toc100153525)

[Ethical Considerations 49](#_Toc100153526)

[Trial Registration 50](#_Toc100153527)

[**8.** **EXPECTED OUTCOME** 51](#_Toc100153528)

[**9.** **BENEFICIARIES** 51](#_Toc100153529)

[**10.** **COLLABORATORS** 51](#_Toc100153530)

[**11.** **TIME TABLE** 53](#_Toc100153531)

[**12.** **BUDGET** 54](#_Toc100153532)

[**13.** **REFERENCES** 55](#_Toc100153533)

[**ANNEX I: Information Sheet** 69](#_Toc100153534)

[**ANNEX II: English Version Questionnaire** 72](#_Toc100153535)

[**ANNEX-III: Sidaamu Afoo Version Questionnaire** 88](#_Toc100153536)

[**ANNEX-IV: Lifestyle Counselling and Pelvic Floor Muscle Training Protocol** 110](#_Toc100153537)

[**ANNEX-IV: Sidaamu Afii Version of Lifestyle Counselling and Pelvic Floor Muscle Training Protocol** 115](#_Toc100153538)

# **LIST OF TABLES**

[Table 1: Sample size calculation based on risk factors of Pelvic Organ Prolapse for the study to be conducted in Sidama National Regional State, Ethiopia, 2022. 28](#_Toc100035625)

[Table 2:Stage of Pelvic Organ Prolapse classified according to the Standardized Pelvic Organ Quantification System (6, 123) 34](#_Toc100035626)

[Table 3: Potential collaborators and their role for the study on Pelvic Organ Prolapse in Sidama Region, Ethiopia, 2022 52](#_Toc100035627)

[Table 4: Time table indicating activity schedules for the study conducted on Pelvic Organ Prolapse in Sidama Region, Ethiopia, 2022 53](#_Toc100035628)

[Table 5: Budget Breakdown for the study conducted on Pe/vic Organ Prolapse in Sidama Region, Ethiopia, 2022. 54](#_Toc100035629)

# **LIST OF FIGURES**

[Figure 1: Conceptual framework indicating risk factors and impacts of Pelvic Organ Prolapse. Compiled from related literature, 2022. 12](file:///C:\Users\user\Desktop\POP\Final%20Submitted%20Proposal.docx#_Toc100153372)

[Figure 2: Map of Hawassa University Health and Demographic Surveillance System Sites from Dale and Wonsho Districts (136) 26](#_Toc100153373)

[Figure 3: Flow diagram of sampling procedure for the study conducted on Epidemiology of Pelvic Organ Prolapse in Sidama National Regional State, Ethiopia, 2022. 30](file:///C:\Users\user\Desktop\POP\Final%20Submitted%20Proposal.docx#_Toc100153374)

[Figure 4: Flow diagram for the cluster randomized control trial study on Effect of Pelvic Muscle Training on Prolapse symptom in Sidama Region, Ethiopia, 2022. 40](file:///C:\Users\user\Desktop\POP\Final%20Submitted%20Proposal.docx#_Toc100153375)

# **ABBREVIATIONS**

| AUGS | American Uro-Gynecology Society |
| --- | --- |
| BMI | Body Mass Index |
| CFA | Confirmatory Factor Analysis |
| CRAIQ-7 | Colorectal-Anal Impact Questionnaire-7 |
| EFA | Exploratory Factor Analysis |
| DHSS | Demographic and Health Surveillance System |
| HRQL | Health Related Quality of Life |
| ICC | Intra-class Correlation Coefficient |
| ICS | International Continence Society |
| IUGA | International Urogynecology Association |
| KMO | Kaiser-Meyer-Olkin |
| NIH | National Institute of Health |
| PCA | Principal Component Analysis |
| PFDI-20 | Pelvic Floor Distress Inventery-20 |
| PFMT | Pelvic Floor Muscle Training |
| PGIC | Patient Global Impression of Change |
| PGI-I | Patient Global Impression of Improvement |
| POP | Pelvic Organ Prolapse |
| POP-SS | Pelvic Organ Prolapse Symptom Score |
| POP-Q | Pelvic Organ Prolapse Quantification |
| P-QoL | Prolapse Quality of Life |
| SCC | Spearman’s correlation coefficient |
| RTC | Randomized Controlled Trial |
| VIF | Variance Inflation Factor |

WHAT IS THE DESSERTATION ABOUT

**LIST OF ORIGINAL PAPERS**

# **SUMMARY**

**Background**: Pelvic Organ Prolapse is a disorder of a woman's pelvic floor characterized by the descent of the uterus, bladder, rectum, or bowel. It is common both in developed and underdeveloped countries. When diagnosed objectively, the prevalence exceeds 50% in general women and increases with age and parity. However, due to the asymptomatic nature of the disease, many women do not seek treatment and suffer from the problem. It affects women’s quality of life highly. Moreover, evidence on the general prevalence of the disease and the effectiveness of conservative treatment is lacking from low-income countries.

**Objectives:** This study is planned to determine the prevalence of the pelvic organ prolapse and evaluate the effect of pelvic floor muscle training for the management of pelvic organ prolapse symptoms and its impact on health-related quality of life in the Sidama region, Ethiopia, 2022.

**Methods:** This study will be conducted in two phases. Initially, the pelvic organ prolapse Symptom Score questionnaire will be translated into Sidaamu Afoo and evaluated for its psychometric properties on a sample of 70 women with pelvic organ prolapse recruited from the gynecology outpatients of Adare General Hospital and Yirgalem Hamline Fistula Center from May 1-25, 2022. Then a complex sample survey will be conducted to establish the prevalence of prolapse based on a sample size of 816 women. It will be conducted in eight kebeles of the Dale and the Wonsho districts from June 1–July 30, 2022. A multistage stratified sampling technique will be used to recruit study participants. The data will be collected through face-to-face interviews with the selected women at the household level. Then all women will be invited to have a pelvic examination at a nearby health facility where the prevalence of the prolapse will be determined by pelvic examination. A complex sample survey analysis will be used to determine the prevalence and determinants of pelvic organ prolapse. In the second phase of the project, a cluster-randomized controlled trial study will be conducted to evaluate the effect of pelvic floor muscle training for the reduction of prolapse symptoms and improving quality of life among 136 (68 participants in each arm). The health-related quality of life will be assessed by using a translated Sidaamu Afoo version of the Prolapse Quality of Life tool, validated in the Amharic version. A Student t-test will be used to evaluate the mean change between the two groups (control and intervention), both for prolapse symptoms and quality of life separately. Linear Mixed-effect Model will be used to control potential confounders in multivariable linear regression for both outcomes.

**Work Plan and Budget**: This study will be conducted from April to November 2022. A total of **762, 295** Ethiopian Birr **($15,245.9)** is needed to conduct this study.

**Key terms:** Ethiopia, Pelvic Organ Prolapse, prolapse, POP, quality of life, pelvic floor muscle exercise, PFMT, pelvic muscle training

# **INTRODUCTION**

## Background

The pelvic organ refers to the uterus and different vaginal compartments with their adjacent organs like bladder, rectum, and bowel. Prolapse refers to falling or slipping or downward displacement of an organ ([1](#_ENREF_1)). Therefore, Pelvic Organ Prolapse (POP) is one of the pelvic floor disorders in women characterized by the descent of one of the vaginal walls, cervix, uterus, bladder, or rectum into the vaginal lumen ([1-6](#_ENREF_1)). Pelvic organs are maintained to their normal position by the support of levator-ani muscle and connective tissue attachments of the vaginal to the pelvis. When the levator-ani muscle or its nerve supply (pudendal nerve) is injured or when connective tissues are damaged, the organs move downward (descent) ([7-10](#_ENREF_7)).

Though it is affecting millions of women worldwide, there was a lack of standardized and validated definitions of POP due to the lack of a document that defined all elements required for its diagnosis comprehensively ([2](#_ENREF_2), [4](#_ENREF_4)). In 1993, the International Consistency Society (ICS), the American Urogynecology Society (AUGS), and the Society of Gynecologic Surgeons (SGS) drafted the first standardized tool for Pelvic Organ Prolapse Quantification (POP-Q) System to measure POP objectively IV ([6](#_ENREF_6)). This tool was developed to reliably describe the support of anterior, posterior, and apical vaginal segments to a fixed vaginal segment, hymen, and established the staging system from stage 0 to stage 4. This definition was simply based on anatomical change but not focused on the relationship between anatomical prolapse and pelvic floor symptoms. It was also challenging to classify which stage is abnormal.

In 2001, the National Institute of Health (NIH) defined vaginal supports as normal only when there is a complete absence of prolapse (stage 0) and abnormal if there is any amount of descent ([11](#_ENREF_11)). It was stated that while the clinical definition of prolapse would include the presence and severity of symptoms, they preferred this classification due to the lack of a large epidemiological study that evaluates the association of prolapse and its symptoms before and after intervention ([5](#_ENREF_5), [11](#_ENREF_11)).

The advantages of POP-Q are that it is specific and objectively quantifies the prolapse. However, it is too complex to understand the measurements as the measurement needs a ruler (centimeter) and nine points in the vagina and around it. Therefore, it was found to be mandatory to have a simple technique for measurement. In 2006, a simplified version of the POP-Q (S-POP) system that retains the ordinal POP-Q stage but simplifies the measurement technique and reduces the number of points measured was established. This technique uses only four points (anterior, posterior, apex/cuff, and cervix) for measurement ([12](#_ENREF_12), [13](#_ENREF_13)).

In 2010, as the body of knowledge increased, both the above two definitions were found to be over-inclusive since the majority of women have a loss of uterovaginal support including stage II POP-Q. It was suggested that the definition should consider the symptoms of prolapse which determine the severity of the disease. Patients with descent beyond hymen who have no symptom of the bulge would be classified as asymptomatic POP and those with bulge should be classified as symptomatic POP ([5](#_ENREF_5)). Mild descent of the pelvic organ is common and should not be considered pathologic, and it should be considered only if causing prolapse symptoms or sexual dysfunction ([14](#_ENREF_14)). The International Urogynecology Association (IUGA) and International Continence Society (ICS) defined POP as the descent of one or more of the anterior vaginal wall, posterior vaginal wall, the uterus, or vaginal vault correlated with relevant POP symptoms ([3](#_ENREF_3)).

Older terms like cystocele, rectocele, uterocele, and enterocele are currently considered meaningless because they describe the protruding organ behind the affected structure of vaginal support. The current definition preferred to characterize the site affected as anterior prolapse, posterior or apical ([3](#_ENREF_3), [9](#_ENREF_9), [10](#_ENREF_10), [14](#_ENREF_14)).

In general, from1996 onwards, there have been improvements in the measurement of POP, and now, there are internationally accepted assessment methods both for structural support and symptoms of the prolapse ([1](#_ENREF_1), [15](#_ENREF_15)). In 2016, the IUGA and ICS concluded that diagnosis of POP, demands clear clinical evidence, starting with women having symptoms related to the downward displacement of the organ. They developed a document that encompasses all required elements for a diagnosis like symptoms, signs, clinical assessment, functional investigation of POP, imaging investigations, terminology for conservative and surgical management. They recommended an interval review (5-10 years) to keep the document updated and widely acceptable ([1](#_ENREF_1)).

POP is usually asymptomatic, and when present, the common symptoms used for POP diagnosis include: a feeling of something coming down from the vagina, uncomfortable feeling in the vagina that worsens when standing, heaviness feeling in the lower abdomen, dragging feeling in the lower back, the difficulty of emptying the bladder, feeling of incomplete bladder emptying and feeling of incomplete bowel emptying ([16](#_ENREF_16)).

The management options include either conservative management (Lifestyle modification, use of pessary or Pelvic Floor muscle Training) or surgically managed. The conservative management is recommended to reduce the risk of POP caused by modifiable risks ([1](#_ENREF_1), [3](#_ENREF_3), [17-19](#_ENREF_17)).

## Statement of the problem

POP is a common condition for which women of all ages and parity seek surgical treatment ([20](#_ENREF_20)). One problem of POP is that it is difficult to establish the exact prevalence due to the difference in the method of diagnosing, use of different tools, the difference in study subjects, and entry criteria ([4](#_ENREF_4), [9](#_ENREF_9), [10](#_ENREF_10), [20](#_ENREF_20)). When diagnosed objectively based on pelvic examination, worldwide, POP affects up to 50% of women ([15](#_ENREF_15)). The risk is increased in low and middle-income countries where the prevalence exceeds 60% ([21](#_ENREF_21), [22](#_ENREF_22)).

The risk factors that have been identified so far are both non-modifiable (family history, ethnicity, and age) and modifiable (obesity, underweight, heavy lifting, and chronic medical problems) ([1](#_ENREF_1), [3](#_ENREF_3), [17-19](#_ENREF_17)).

Even though mortality is uncommon in POP, prolapse often co-exists with other disorders that affect women's quality of life. A study from southern California shows that 69% of POP cases were presented with other pelvic floor disorders (37% overactive bladder, 40% stress urinary incontinence and 50% have fecal incontinence). Similarly, a review of published literature shows that overactive bladder co-existence with POP varies from 10-50% ([23](#_ENREF_23), [24](#_ENREF_24)). A systematic review and meta-analysis of papers published from 1996 to 2016 show that hydronephrosis was presented in 3.5% to 30.6% of cases ([25](#_ENREF_25)).

Co-existence of these diseases aggressively affects women's quality of life and in addition to the immediate health problems. A community-based study from Pakistan showed that 60.8% of POP patients reported that POP has a great impact on their overall quality of life ([26](#_ENREF_26)). Qualitative studies from Swedish and northern parts of Ethiopia revealed that POP patients were suffering to handle their daily activities due to the symptom of POP like bulging mass through the vagina while lifting weights ([27](#_ENREF_27), [28](#_ENREF_28)). POP has also a psychological impact on which women were suffering from depression ([29](#_ENREF_29), [30](#_ENREF_30)). Stigma and discrimination due to POP were reported to result in a psychological burst ([27](#_ENREF_27), [28](#_ENREF_28)).

POP also causes a vaginal bulge, voiding dysfunction, and sexual dysfunction which adversely affects the quality of life ([9](#_ENREF_9), [14](#_ENREF_14)). Sexual function is complex and may not be managed with surgery for POP. The relationship status and sexual desire might not improve by structural change (surgery) ([31](#_ENREF_31)). It was reported that sexual dysfunction like a disorder of sexual desire, arousal, and pain is common with a patient with anterior prolapse ([32](#_ENREF_32)).

Moreover, sometimes the treatment of POP by itself has different complications like infection, irritation, bleeding, pain, ulceration, and failure rate ([9](#_ENREF_9), [33](#_ENREF_33))

With these all complications (pain and severe difficulties carrying out daily activities), women with POP do not disclose it and refrain from seeking treatment. The disclosure is related to discrimination, stigma, and divorce ([27](#_ENREF_27)). Even in developed countries, women are ashamed of disclosing POP even to a health professional and suffer from the problem ([34](#_ENREF_34)). Almost 80% of POP patients from low-income countries were delayed at least for 12 months before seeking treatment even for severe degree prolapse ([26](#_ENREF_26), [35](#_ENREF_35), [36](#_ENREF_36)). POP is also a public health burden (beyond individual burden), it decreases the quality of life, increase the use of health care resource and decrease productivity ([37](#_ENREF_37)). Moreover, as a result of lifestyle modification, it is expected that the number of elderly population will increase from time to time, and the POP will become more prevalent shortly and increase economic and public health burden ([2](#_ENREF_2), [4](#_ENREF_4), [15](#_ENREF_15)). As its current status, the high cost for treatment and risk of recurrence after surgery makes POP both a health and financial burden ([2](#_ENREF_2)).

In Ethiopia, in addition to the universally identified risk factors, there are additional risk factors that are expected to increase the prevalence of POP. Women in Ethiopia are prone to a high number of vaginal deliveries, early marriage, and early childbirth, heavy lifting/carrying, and low body mass index ([38-40](#_ENREF_38)). Previous studies show that the prevalence of POP varies from 1-56.3% based on the diagnosis method used and study settings ([39](#_ENREF_39), [41](#_ENREF_41)). Most of the previous studies were health facility-based ([27](#_ENREF_27), [35](#_ENREF_35), [36](#_ENREF_36), [40](#_ENREF_40)), and the others were conducted using the symptomatic approach for POP diagnosis ([41](#_ENREF_41)). Both approaches have a high probability of underestimating the prevalence of POP. A study from Dabat district, Northern Ethiopia ([39](#_ENREF_39)), deserves acknowledgment for revealing the difference in the prevalence of POP when diagnosed by symptom score (28%) and thorough pelvic examination (56.3%). However, it has missed some important factors like BMI, family history, and ethnicity. Analysis for certain variables like age and number of delivery were based on very few (single) observations. Besides that, while evidence from developed countries shows that pelvic floor muscle exercise reduces pelvic organ prolapse symptoms and health-related quality of life ([42-44](#_ENREF_42)), there is a lack of evidence from low-income countries like Ethiopia. The use of pessary and pelvic floor muscle exercise is not yet implemented in Ethiopia (except the one under the Pilot study for pessary implementation).

We could not get any published data from Sidama region on this topic so far. Therefore, this study aims to determine the prevalence of and risk factors for POP in the community using both objective and subjective/symptom-based measures. The study will also pilot the feasibility and effectiveness of pelvic floor muscle exercise for the treatment of POP symptoms and health-related quality of life.

# **LITERATURE REVIEW**

## Prevalence of Pelvic Organ Prolapse

It is too challenging to establish the population prevalence of POP as the prevalence varies widely based on the criterion used to diagnose the condition. In addition, the category of study population affects the prevalence of POP ([45](#_ENREF_45)). In general, a review of the literature shows that anterior prolapse is two times more common than posterior prolapse and three times common than apical prolapse ([9](#_ENREF_9), [26](#_ENREF_26)). Most women with POP are asymptomatic and the actual prevalence may differ when diagnosed by symptom (patient self-report) and thorough pelvic examination ([7](#_ENREF_7), [8](#_ENREF_8), [10](#_ENREF_10)). For instance, a review of English language scientific literature published before January 2012 reported that the prevalence of POP was 3-6% when diagnosed by symptom and reach up to 50% when diagnosed based on pelvic examination ([15](#_ENREF_15)) on the same population.

In Netherland, a study that assessed the entire population of a small town reported that 40% of women aged 45 to 85 years had POP stage II and above on pelvic examination, but only 12% of them were symptomatic ([46](#_ENREF_46))**.** A community-based study in East Lebanon shows that 49.8% of women aged 15-60 years had clinically significant POP ([37](#_ENREF_37)). In Turkish, among women visiting university hospitals, Prolapse stage ≥2 was detected in 27.1% of patients ([47](#_ENREF_47)). In Maryland, among women older than 40 years and appointed for surgery, POP beyond the hymen was observed in 24.8% of cases ([48](#_ENREF_48)).

A community-based study conducted in rural China shows that the prevalence of symptomatic POP (confirmed stage II and above among women who complain symptom) was 9.1% ([49](#_ENREF_49)). Another study conducted in China among obese women show that the POP prevalence was 15.8% ([50](#_ENREF_50)). These studies may be underreported since non-symptomatic cases were not examined. The narrative review on POP prevalence and diagnosis 2019, concludes that many women who suffer from POP is may be due to the asymptomatic nature of the disease or they may not seek medical attention for other reasons ([4](#_ENREF_4)).

In 2011, a review of different literature from 16 low and middle-income countries reported that the mean prevalence of pelvic organ prolapse is 19.7% which ranges from 3.4 to 56.4% ([21](#_ENREF_21)). Similarly, in the rural community of Gambia, among women who underwent pelvic examination, 46% have some degree of prolapse from which only 13% of women with moderate to severe prolapse reported symptoms ([51](#_ENREF_51)). In rural Nepal among mothers visiting rural health centers for health care seeking, 60.9% were diagnosed to have POP stage II and above ([22](#_ENREF_22)).

In Ethiopia, the prevalence of POP varies from 1-56.3% based on the diagnosis method used and study settings. Based on the symptomatic approach, POP was diagnosed among 1% -28% of women. However, pelvic examination of the same population showed that 56.3% of them have at least grade two prolapses ([38](#_ENREF_38), [39](#_ENREF_39), [41](#_ENREF_41), [52](#_ENREF_52)). Despite its high prevalence, women are not seeking treatment and suffer from the problem due to fear of stigma, lack of access to treatment, and financial problems ([21](#_ENREF_21), [26](#_ENREF_26), [35](#_ENREF_35), [36](#_ENREF_36)).

## Risk Factors for Pelvic Organ Prolapse

So far, different studies have identified various risk factors for the development of POP. The risk factors were identified to contribute to either weakening of the collagen (connective tissue) or damage to levator-ani muscle and its nerve supply. Defects in the collagen and levator-ani muscle are important etiologic factors for the development of POP ([4](#_ENREF_4), [8](#_ENREF_8), [53](#_ENREF_53), [54](#_ENREF_54)). While all literature agrees on some risk factors like the risk of old age and vaginal birth, there are also contradicting findings on the role of certain factors like physical activity, body mass index, hysterectomy, and race/ethnicity. The findings of various literature were presented as follows.

1. **Sociodemographic and economic risk factors**

**Age**: Ageing is one of the most commonly reported risk factors for POP. The proposed effect of age on POP is maybe the combination of physiologic aging, hypo-estrogenic, and age-related degenerative disease. Aging plays a complex role in the pathogenesis of organ prolapse ([7](#_ENREF_7), [8](#_ENREF_8)). Two recent (2017) community-based studies from rural China show that there is a consistent increase in POP prevalence with increasing age. Compared to 20-29-year-old women, the risk increases twofold for those older than 50 years and by fivefold for those older than 70 years ([49](#_ENREF_49), [50](#_ENREF_50)). A community-based study from East Lebanon also shows that POP prevalence dramatically increases with age which reaches up to 75% among 50-59 years women and become plateau phase after that ([37](#_ENREF_37)). A similar study from Brazil and Pakistan also reported that age 35 years or older increases the risk of POP almost six-fold ([26](#_ENREF_26), [55](#_ENREF_55)).

In Ethiopia, three studies from the northern part of Ethiopia and one systematic review and meta-analysis study also confirmed that age greater than 40 years were significantly associated with POP ([38-40](#_ENREF_38), [56](#_ENREF_56)). In general, all studies that assess the association of age with POP agree that the risk of POP increase with age.

**Level of education**: A community-based study from Turkey and Ethiopia showed that POP is more associated with a lack of formal education ([56](#_ENREF_56), [57](#_ENREF_57)). This might be due to the high probability of homebirth and increased number of vaginal birth among uneducated females.

**Carrying heavy objectives:** An online survey data collected from 3,934 women, compared the symptom of POP among women categorized by the amount of weight lifting for exercise (<15Kg, 16 –50kg, >50 kg, and inactive women). The proportion of women with symptoms is 7% in a heavy lifting group (>50kg) and 21% among inactive groups ([58](#_ENREF_58)). Another study conducted among nulliparous women with at least 6-months history of weight lifting reported a relatively low prevalence of POP ([59](#_ENREF_59)). However, studies from low-income countries and rural China shows that POP is more prevalent among women who perform physical labor ([21](#_ENREF_21), [26](#_ENREF_26), [39](#_ENREF_39), [49](#_ENREF_49), [60](#_ENREF_60)). One community-based study from Tanzania and two studies (institution-based) from Ethiopia show that carrying heavy objectives for more than five hours was a significant risk factor for POP ([40](#_ENREF_40), [61](#_ENREF_61)). Even a two-hour daily carrying heavy objective was associated with POP ([39](#_ENREF_39)).

**Ethnicity/race:** Studies that compared different ethnic groups reported that the risk of POP differs across ethnic groups. A prospective study from Netherland and China reported that apical prolapse is common in china whereas anterior prolapse is common in Caucasians ([62](#_ENREF_62)). Studies from America that compared white and black women reported that white women are more suffer from POP than black women ([53](#_ENREF_53), [63](#_ENREF_63), [64](#_ENREF_64)). It is justified that black women commonly have a narrow pubic arch that is protective against POP compared to the gynecoid pelvis ([7](#_ENREF_7), [8](#_ENREF_8)). However, two studies from Uganda and South Africa show that black women developed more POP than Caucasian women ([65](#_ENREF_65), [66](#_ENREF_66)). A community-based study from rural Gambia showed that Wolof ethnicity was more at risk of POP compared to Mandinka ethnic group ([51](#_ENREF_51)). This was explained as it is pointing to genetic factor or cultural factor. In Ethiopia, evidence about the effect of ethnicity is not reported.

1. **Obstetrics and gynecologic risk factors**

**Vaginal delivery**: Similar to aging, vaginal delivery is the commonest risk factor of POP, for which the mechanism of action is identified. Ultrasound-assisted investigations show that during vaginal delivery, direct and indirect injury to the pelvic muscle by compression of the fetal part and neurologic injury from over-stretching of the pudendal nerve is common ([4](#_ENREF_4), [7-10](#_ENREF_7)).

In Maryland, a large cohort of women who gave birth showed that vaginal delivery carries a risk compared to cesarean delivery ([67](#_ENREF_67)). A systematic review and meta-analysis study that assess the association between mode of delivery and POP in 2018, showed that vaginal delivery has an increased risk of POP compared to cesarean delivery ([68](#_ENREF_68)). There are also similar findings from community-based studies from China and Brazil ([49](#_ENREF_49), [50](#_ENREF_50), [58](#_ENREF_58), [69](#_ENREF_69)). In general, large-scale cross-sectional studies;and systematic review and Meta-analysis studies confirmed that vaginal delivery has the strongest association with POP as compared to cesarean delivery ([58](#_ENREF_58), [70](#_ENREF_70)). Studies conducted in Ethiopia were more focused on the effect of parity than the mode of delivery ([39](#_ENREF_39), [56](#_ENREF_56)).

**Number of childbirth (parity):** Several studies were reported that parity was a risk for POP. Community-based studies from China, Lebanon, and Swedish reported that the risk of POP increased at least twice with parity ([37](#_ENREF_37), [71](#_ENREF_71)). However, a retrospective review of 1,746 women from Sydney published in 2020 shows that even though vaginal birth affect POP, the number of birth did not associate with POP ([72](#_ENREF_72)). Studies from developing countries like Nigeria and Ethiopia (where high parity is common) show that POP is associated with high parity ([39](#_ENREF_39), [40](#_ENREF_40), [56](#_ENREF_56), [73](#_ENREF_73), [74](#_ENREF_74)).

**Forceps Assisted delivery:** Forceps-assisted deliveries were associated with POP compared to spontaneous vaginal delivery. Community-based cohort studies from Maryland and Swedish (from 2005-2015), show that the risk of developing POP and the need for surgery is increased among women assisted with forceps compared to those not assisted with forceps ([67-69](#_ENREF_67)). A systematic review and Meta-analysis published by 2021 also reported that forceps delivery is a risk for symptomatic POP ([70](#_ENREF_70)). Evidence about the effect of instrumental delivery on POP is scarce in Ethiopia.

**Prolonged labor:** Prolonged and obstructed labor were identified to increase the risk of POP ([18](#_ENREF_18)). Institution-based studies from Nigeria and Hospitals of the Amhara region, Ethiopia reported that prolonged labor was significantly associated with POP ([40](#_ENREF_40), [73](#_ENREF_73)).

**Age at first delivery:** A Study conducted in Amhara referral hospitals among patients visiting gynecology clinic show that age less than 20 years at first delivery was significantly associated with increased risk of POP ([40](#_ENREF_40)).

**Menopause:** While the effect of menopause is related to aging, studies have shown that there is a direct relationship between menopause and increased risk of POP irrespective of age and parity. The hormonal change during menopause causes a drop in estrogen hormone and affects the strength of collage ([4](#_ENREF_4), [75](#_ENREF_75)). A retrospective study from Nigeria shows a strong association between POP and menopause ([73](#_ENREF_73)). In Ethiopia, one study conducted in the Gurage zone showed that menopause was associated with pelvic floor disorder ([76](#_ENREF_76)). However, there is no adequate report specifically on the association of menopause and POP.

**Hysterectomy:** History of pelvic surgery, especially hysterectomy, was found to risk one of vaginal compartment prolapse. It was evidenced that intraoperative damage to connective tissues, blood supply, and nerves was found to increase central compartment prolapse ([4](#_ENREF_4)). A follow-up study of women who underwent surgery from 2015-17 in Mekele, Ethiopia, showed that vaginal repair has an anatomical failure in 34% and symptomatic recurrence in 23% ([33](#_ENREF_33)).

1. **Nutritional factors**

Studies from developed countries show that obesity and overweight were associated with POP. The possible way in which obesity risks to POP is maybe through increasing intra-abdominal pressure ([7](#_ENREF_7), [8](#_ENREF_8), [77](#_ENREF_77)). Two systematic reviews and meta-analyses of papers from developed countries show that a higher body mass index > 24kg/m^2^ was a significant risk factor for primary POP ([17](#_ENREF_17), [78](#_ENREF_78)). A community-based study from East Lebanon and China also shows that a higher body mass index remains a significant risk factor for POP ([37](#_ENREF_37), [49](#_ENREF_49)). In contrast to the above findings, studies from Ethiopia show that lower body mass index (< 18kg/m^2^) was associated with POP ([38](#_ENREF_38), [56](#_ENREF_56), [79](#_ENREF_79)). It was justified that chronic under-nutrition may cause poor tissue tensile strength ([21](#_ENREF_21)). Two recently published systematic review and meta-analysis studies reported that there was no association between POP and BMI ([74](#_ENREF_74), [80](#_ENREF_80))**.**

1. **Family history (genetic) of pelvic organ prolapse**

Genetic predisposition may aggravate or impact POP development as the strength of collagen (the main component of connective tissue) that supports pelvic facia is determined by genetic factors ([4](#_ENREF_4)). In developed countries, the association between family history/genetics and POP was well researched. A population-based study that involved 4628 women diagnosed with POP and their families shows that the risk of developing POP is increasing with the number of family histories affected by POP. History of at least one first-degree relative has increased the risk of POP by two fold, and history of two first-degree relatives increased the risk more than three fold ([81](#_ENREF_81)). One systematic review and meta-analysis also show that on average, a positive family history of POP increases the risk threefold ([82](#_ENREF_82)). This is similar to a case-control study that revealed a family history of prolapse has remained associated with advanced prolapse ([83](#_ENREF_83)). Another study that assessed family history of POP among women who underwent gynecological surgery shows that family history has increased the risk ([84](#_ENREF_84)). A case-control study from Bahir-Dar also reported that a family history of POP was associated with POP ([56](#_ENREF_56)).

1. **Medical history**

**Constipation:** Reports from China, Korea, Nigeria, and Ethiopia show a significant association between POP and constipation ([40](#_ENREF_40), [50](#_ENREF_50), [56](#_ENREF_56), [73](#_ENREF_73)). However, one observational study and systematic review of papers from developed countries show that there is no association ([17](#_ENREF_17), [63](#_ENREF_63)).

**Chronic cough**: Studies from rural China and Nigeria reported that chronic cough (>3weeks) was associated with symptomatic POP ([49](#_ENREF_49), [73](#_ENREF_73)). Intra-abdominal pressure caused by chronic cough and constipation causes excessive strain on the pelvic structure including the pudendal nerve ([4](#_ENREF_4), [50](#_ENREF_50)).

**Diabetes mellitus**: Systematic review and meta-analysis of papers from developed countries only, and retrospective study of managed cases at Turkey tertiary hospitals show that diabetes mellitus was a risk factor for POP ([17](#_ENREF_17), [85](#_ENREF_85)). If there are co-morbidities like diabetes mellitus, there will be poor tissue features through neuropathy and genetic factors that predispose to POP ([4](#_ENREF_4), [50](#_ENREF_50)). Reports on the effect of chronic medical cases are scarce from Ethiopia.

## Conceptual Framework

Based on the findings of the above literature, the following conceptual framework is developed (Figure 1). The conceptual framework shows that the different risk factors cause POP either through direct damage of pelvic muscles (levator-ani muscle and its nerve supply) or by causing collagen to weaken ([37](#_ENREF_37), [39](#_ENREF_39), [47](#_ENREF_47), [50](#_ENREF_50), [56](#_ENREF_56), [57](#_ENREF_57), [61](#_ENREF_61), [65](#_ENREF_65), [68](#_ENREF_68), [76](#_ENREF_76), [78](#_ENREF_78), [81](#_ENREF_81), [86](#_ENREF_86), [87](#_ENREF_87)).

**OBESTETRICS/GYNECOLOGY**

- Vaginal delivery
- Parity
- Instrumental delivery
- Age at 1^st^ delivery
- Prolonged labour
- Menopause
- History of pelvic surgery/hysterectomy

**SOCIO-DEMOGRAPY**

- Age
- Level of education
- Occupation (weight lift)
- Ethnicity/race

**MEDICAL CASES**

- Chronic cough
- Constipation
- DM

DM

**Family History of Pelvic Organ Prolapse**

**Pelvic Organ Prolapse (POP)**

**Nutritional factors**

- BMI > 24Kg/m^2^
- BMI < 18 Kg/m^2^

**Pelvic Organ Prolapse symptoms**

**Pelvic Floor Muscle Training**

**Prolapse-QoL**

Figure 1: Conceptual framework indicating risk factors and impacts of Pelvic Organ Prolapse. Compiled from related literature, 2022.

## Impact of POP on Health-Related Quality of Life

POP is a complicated disorder with both physical and functional elements ([88](#_ENREF_88)), and as a result, it can have a substantial influence on the quality of life and psychological well-being of women who are affected. Indeed, prolapse can cause a number of urinary, bowel, and sexual disorders, all of which can negatively impact a patient's quality of life. Sexual dysfunctions, in particular, are quite frequent in women with POP ([32](#_ENREF_32), [88](#_ENREF_88), [89](#_ENREF_89)). POP patients usually complain problems with sexual desire, arousal, orgasm, and pain, which can lower their quality of life and negatively impact their relationships ([32](#_ENREF_32)). Social, physical, and psychological constraints caused by pelvic organ prolapse may have an impact on a patient's interpersonal connections also ([90](#_ENREF_90), [91](#_ENREF_91)). A study conducted among 3,114 middle aged women (50 -61) reported that, the presence of POP symptoms was linked to a lower quality of life score on each domain of health (physical mobility, pain, emotional reaction, social isolation, energy, and sleep ([92](#_ENREF_92)). Other similar studies also reported that POP causes physical, social, psychological, occupational, household, and/or sexual limits in women, producing severe symptomatic distress and poor quality of life ([90](#_ENREF_90), [92](#_ENREF_92)). Moreover, it is recommended that the major goal of treatment should be to improve health-related quality of life by reducing this symptoms. For the clinical assessment to develop more appropriate treatment, the severity and impact of prolapse symptoms on quality of life are critical ([93](#_ENREF_93)).

## Interventions against POP

The risk factors that have been identified to be predisposing factors are both non-modifiable (family history, ethnicity, and age) and modifiable (obesity, underweight, heavy lifting, and chronic medical problems). Lifestyle modification is recommended to reduce the risk of POP caused by modifiable risks ([1](#_ENREF_1), [3](#_ENREF_3), [17-19](#_ENREF_17)). Once the POP is developed it is managed either by conservative management or by surgical management.

### Conservative Management

Recent evidence and guidelines recommend conservative management for mild degree prolapse with no symptoms ([1](#_ENREF_1), [42](#_ENREF_42), [94](#_ENREF_94)). Non-surgical therapy such as pessary, pelvic floor muscle training, or both can help with symptom relief as well as weight loss in obese people. However, for women with severe prolapse, most of these treatments are ineffective; hence, surgical therapy is more appropriate rcumstances ([95](#_ENREF_95), [96](#_ENREF_96)).

1. **Pessary**: is a device inserted into the vagina to support the descending vaginal compartment ([3](#_ENREF_3), [94](#_ENREF_94)). While it is effective in managing mild degree prolapse, there are risks of infection and irritation ([9](#_ENREF_9), [19](#_ENREF_19), [97](#_ENREF_97)). Moreover, the use of pessary is not well implemented in low-income countries including Ethiopia (except the one under pilot study).
2. **Pelvic Muscle Training**: The importance of Pelvic Floor Muscle Training to relieve POP symptoms and its effectiveness was frequently confirmed by Randomized Control Trials from developed countries. However, still, evidence from low-income countries is lacking on its feasibility and effectiveness. In this document, the findings from previous literature are discussed in detail, as it is part of the current and planned study.

Trial studies were conducted in westerns to evaluate the effect of pelvic floor muscle exercise on the reduction of prolapse symptoms, improving prolapse stage, and prevention of secondary prolapse. A review of six trail papers published between 1996-2010 shows that pelvic floor muscle exercises improved prolapse symptoms by 17% compared to the control groups ([94](#_ENREF_94)). A randomized control trial conducted on women aged 55 years and over with mild symptoms shows that 57% of women in the pelvic floor muscle training (PFMT) group have improved symptoms after 3months of training while improvement was seen only in 13% of control groups ([44](#_ENREF_44)).

Another systematic review of 13 papers which included 2,340 patients also shows that women in the Pelvic Floor Muscle Training get improvement than the control groups in prolapse symptom score with a mean difference of -3.07 ([98](#_ENREF_98)).

A study conducted among 109 Scandinavian-speaking women with POP stage I –III show that 39% of women who received lifestyle advice and Pelvic Floor Muscle Training reported improved sexual function compared to only 5% of women who received lifestyle advice only ([99](#_ENREF_99)). Similarly, a multicentre randomized control trial conducted in 23 centers in the United Kingdom, New Zealand, and Australia show that after 16 weeks of individualized pelvic muscle training, 12 months of prolapse symptoms were significantly less in interventional groups ([42](#_ENREF_42)).

There are contradicting findings on regarding the effect of PFMT on improving the prolapse stage. A randomized control trial study in Norway involving 109 women shows that Pelvic Floor Muscle Training improved at least one pelvic organ prolapse quantification system stage in 19% of participants ([100](#_ENREF_100)). However, other controlled trial studies reported that there was no change in the prolapsed stage ([42](#_ENREF_42), [101](#_ENREF_101)). Other studies reported that the effect of physical exercise on pelvic muscle strength is controversial and needs an adequate sample size for generalization ([101](#_ENREF_101), [102](#_ENREF_102)). A recent (2021), Systematic review and meta-analysis of randomized control trial studies concluded that even though PFMT significantly changed the self-reported symptom as improved, there was no distinct difference on POP-SS ([103](#_ENREF_103)).

To evaluate the effect of various treatments to treating POP, the most significant outcome is quality of life. It is suggested that researchers explain how POP treatment affects quality of life. A vast number of researches have investigated the impact of various treatments by assessing quality of life ([94](#_ENREF_94), [104](#_ENREF_104)). Randomized control trial studies show that PFMT has significantly improved prolapse-quality of life. An assessor blinded pilot study conducted on 29 POP patients for 16 weeks showed that POP-related symptoms such as stress urine incontinence, frequency, post micturition dribbling, and bowel obstruction decreased dramatically. As a result, the overall health, physical limits, emotion, and severity assessments all had considerably improved after PFMT ([105](#_ENREF_105)). Similarly, a systematic review and meta-analysis study conducted to evaluate quality of life after POP treatments showed that PFMT had a relative influence on quality of life ([106](#_ENREF_106)). Another two randomized control trial studies also showed that PFMT significantly improved bowel-related quality of life ([107](#_ENREF_107), [108](#_ENREF_108)).

While these all findings are from developed countries, there is a lack of evidence from low-income countries including Ethiopia.

### Surgical management of POP

In developing countries, women seek treatment at an advanced stage of prolapse and are usually managed surgically ([26](#_ENREF_26), [35](#_ENREF_35), [56](#_ENREF_56), [61](#_ENREF_61), [73](#_ENREF_73)). Surgical management is mainly classified as reconstructive and obliterative. Obliterative surgery is usually performed for elderly women who do not need future fertility. The procedure includes removal of the descended vaginal compartment and closing of the vagina (the uterus may or may not be removed). Reconstructive surgery is aimed to maintain the normal pelvic anatomy. The procedure involves paravaginal repair and vaginal vault suspension by utero-sacral ligaments ([3](#_ENREF_3), [19](#_ENREF_19), [33](#_ENREF_33)). Currently, surgical management of POP is challenging as the need for uterine preservation is increased ([109](#_ENREF_109)). The procedure can be performed vaginally or abdominally ([3](#_ENREF_3), [19](#_ENREF_19), [33](#_ENREF_33)).

While it is the commonly used treatment technique in low-income countries, still it has both anatomical and symptomatic recurrence. Intraoperative damage to connective tissues, blood supply, and nerves was found to increase central compartment prolapse ([4](#_ENREF_4)). A follow-up study of women who underwent surgery from 2015-17 in Mekele, Ethiopia, showed that vaginal repair has an anatomical failure in 34% and symptomatic recurrence in 23% ([33](#_ENREF_33)).

## Justification of the Proposed Study

In addition to the universally identified risk factors, there are additional risk factors in Ethiopia that are expected to increase the prevalence of POP. Women in Ethiopia are prone to a high number of vaginal deliveries, early marriage, early childbirth, heavy lifting/carrying, and low body mass index ([38-40](#_ENREF_38)).

Studies conducted in Ethiopia on the POP prevalence and risk factors were systematically searched in PubMed using key terms and 23 studies were identified. Google Scholar, African Journal of Online, and Directory of Open Access Journals were also searched for important literature. Most of the identified studies were health facility-based ([27](#_ENREF_27), [35](#_ENREF_35), [36](#_ENREF_36), [40](#_ENREF_40)); and the other studies were conducted using the symptomatic approach for POP diagnosis ([41](#_ENREF_41)). Both approaches have a high probability of underestimating the prevalence of POP. It is evidenced that pain and severe difficulties while carrying out daily activities were common with POP patients. However, patients do not disclose it and seek treatment since the disclosure was related to discrimination, stigma, and divorce ([27](#_ENREF_27)). Almost 80% of POP patients from Pakistan and Ethiopia were delayed at least for 12 months before seeking treatment for severe degree prolapse ([26](#_ENREF_26), [35](#_ENREF_35), [36](#_ENREF_36)). Therefore, a community-based study is important to reveal the prevalence in at early stage in the general population. Unlike the previous studies, the risk factors will be identified by using a complex survey analysis that matches with sampling design.

Moreover, since there is a lack of evidence about the effect of PFMT in prolapse symptom management in low-income countries like Ethiopia, this study will evaluate its effect both on prolapse symptom and quality of life.

## Significance of the Study

Understanding about the epidemiology of POP has both clinical and public health importance. In general, POP has significant public health importance as it decreases the quality of life, increase the use of health care resource and decrease productivity ([37](#_ENREF_37)). It is an important public health issue that needs to be a focus of primary prevention ([110](#_ENREF_110)). As a result of lifestyle modification, it is expected that the number of elderly population will increase from time to time, and the POP will become more prevalent shortly and increase economic and public health burden ([2](#_ENREF_2), [4](#_ENREF_4), [15](#_ENREF_15)). The increase in life expectancy and improved quality of life increase both the prevalence of disease and the number of treatment-seeking women ([4](#_ENREF_4)). As a public health concern, at least to allocate resources to women with POP, an understanding of its incidence/prevalence, risk factors, clinical implication, and treatment options are needed to treat and develop preventive strategies. Identifying modifiable risk factors may also help for patient counselling and prevention in patients at risk ([2](#_ENREF_2)). Since the risk factors of POP are multifactorial, the approach to risk factor modification and prevention may also differ across settings ([63](#_ENREF_63)). So, to develop the best and most- effective preventive and therapeutic strategies, the prevalence and risk factors at the community level should be defined ([37](#_ENREF_37)). Therefore, this study will determine the prevalence and identify associated risk factors of POP among women in Sidama National regional State. The regional health bureau and other organizations working on women's health will use the findings.

On the Clinical aspect, as its current status, the high cost for treatment and risk of recurrence after surgery makes POP both a health and financial burden ([2](#_ENREF_2)). POP is currently a common disorder that has a remarkable economic burden on the health system and individuals ([7](#_ENREF_7)). Health care providers need to understand epidemiology to screen for the disorders and treat them to improve quality of life and decrease the economic burden of individuals and the health care system ([111](#_ENREF_111)).

The demand for conservative management is common especially for women giving birth at an older age ([109](#_ENREF_109)). Moreover, currently, the most important outcome of POP treatment is health-related quality of life and patient satisfaction ([14](#_ENREF_14), [112](#_ENREF_112)). POP causes a vaginal bulge, voiding dysfunction, and sexual dysfunction which adversely affects the quality of life ([9](#_ENREF_9), [14](#_ENREF_14)). Sexual function is complex and may not be managed with surgery for POP. The relationship status and sexual desire might not improve by structural change (surgery) ([31](#_ENREF_31)).

If the result of the current study is effective in improving prolapse symptoms and quality of life, the intervention will be expanded to wider areas. Therefore, evaluating the effectiveness of this non-surgical, cheap and no side effect treatment option has important value to improve women's health. This study will also reveal how much women’s health-related quality of life is affected by prolapse symptoms. This in turn will contribute to enabling the family and community to understand and help women with pelvic organ prolapse. The long-term goal of this study is to produce knowledge used to improve health care for women with Pelvic Organ Prolapse in Sidama National Regional State, Ethiopia.

# **OBJECTIVES**

# **3.1** General Objective

To Assess the Prevalence and risk factors of Pelvic Organ Prolapse; and Effect of Pelvic Floor Muscle Training on Prolapse Symptom and Quality of Life in Sidama National Regional State, Ethiopia, 2022.

# **3.2.** Specific Objectives

1. To Translate the Pelvic Organ Prolapse Symptom Score (POP-SS) into Sidaamu Afoo and Evaluate its Psychometric Properties.
2. To determine the prevalence and identify the risk factors of pelvic organ prolapse in Sidama National Regional State
3. To evaluate the effect of Pelvic Floor Muscle Training for reducing pelvic organ prolapse symptoms among women with POP in Sidama National Regional State
4. To evaluate the effect of Pelvic Floor Muscle Training to improve Prolapse Quality of Life among women with POP in Sidama National Regional State.

# **METHODS**

This study will be conducted in two phases. Phase one is used to address objective one and two, and the second phase will be conducted to answer objectives three and four.

# **PHASE-I PROJECT**

# **PAPER-I**

Validation of the Sidaamu Afoo version of the Pelvic Organ Prolapse Symptom Score in Sidama National Regional State, Ethiopia, 2022.

## Objective

To Translate the Pelvic Organ Prolapse Symptom Score into Sidaamu Afoo and evaluate its psychometric properties among Sidaamu Afoo speaking women, in Ethiopia, 2022.

## The Original POP-SS questionnaire

The original version of the POP-SS was initially developed by Professor Suzan Hagen in English Speaking women ([16](#_ENREF_16)). Later it was translated to and validated in Turkish, Amharic, and Chinese ([113-115](#_ENREF_113)). The POP-SS has seven questions with a five-point Likert scale which ranges from zero to four (0= never felt symptom, 1= occasionally, 2= sometimes, 3= most of the time, and 4= all of the time), a higher score indicating severe symptoms ([16](#_ENREF_16)). The participants will be asked how often they have had the following symptoms in the last four weeks: 1) A feeling of something coming down from the vagina? 2) Uncomfortable feeling in the vagina that worsens when standing? 3) A heaviness feeling in the lower abdomen? 4) A dragging feeling in the lower back? 5) A difficulty of emptying the bladder? 6) A feeling of incomplete bladder emptying? 7) A feeling of incomplete bowel emptying? The total score will be calculated by summing up all responses for the seven-question and the total score will range from 0 to 28. If the participants' total response score is different from zero, she will be considered as having symptoms of prolapse.

To increase the accuracy of measurements, instrument (data collection tool) translation, cross-cultural adaptation, and validation for the language of the target population are mandatory ([116-118](#_ENREF_116)). Therefore, this study is aimed to translate and validate the POP-SS tool in Sidamu Afoo.

## Translation and Cultural Adaptation of POP-SS

Translation and cultural adaptation of the POP-SS tool will be conducted according to the standard method of translation and adaptation recommendation ([117](#_ENREF_117)). The permission to translate the tool will be received from the original author of the POP-SS, Professor Suzan Hagen ([16](#_ENREF_16)).

**Forward Translation**: Three native Sidaamu Afoo speakers (two gynecologists and one sociologist) who are fluent in English will translate the tool from English to Sidaamu Afoo independently. A common draft version of Sidamu Afoo will be produced from the three translated versions in consensus between the three authors and one language expert. This version will be back-translated into English by two independent fluent English and Sidaamu Afoo speakers. The original and back-translated versions will be checked for any discrepancies by the authors and referred back to the developers for conceptual equivalence. After incorporating necessary changes or comments, the first Sidaamu Afoo version will be produced.

For expert committee review, expertise from gynecology, midwifery, public health, and Sidaamu Afoo instructors will review the final forward and backward translations against the original version. All issues, if present, will be addressed and a preliminary version will be created and circulated among review members. The expert committees will evaluate the suitability of each item and rate its relevance. The agreement will be calculated using the Content Validity Index ([119](#_ENREF_119)).

To evaluate the equivalence and comprehensibility of the translated Sidaamu Afoo version, it will be face-validated and pretested among ten women who speak Sidaamu Afoo and admitted to Adare General Hospital and Yirgalem Hamlin Fistula Center with a diagnosis of POP.

## Study Participants

The study participants will be women aged 18 years and above who visit the gynecology outpatient department (clinics) of Adare General Hospital and Yirgalem Hamlin Fistula Center from April 01 – to May 30, 2022 will be recruited consecutively.

## Sample Size

To ensure the statistical robustness of the analysis, the sample size is determined based on the recommendation of at least 5-10 subjects per item of the tool by the consensus-based Standards for the Selection of the Health Measurement Instruments (COSMIN) ([120](#_ENREF_120), [121](#_ENREF_121)). Accordingly, the estimated sample size is 70 (10 subjects per item).

## Sampling Procedure

All women who visit gynecology outpatient departments of Yirgalem Hamlin Fistula Center and Adare General Hospitals during the study period will be assessed for the presence of prolapse symptoms by two important questions. Women will be asked whether they had a feeling of bulging/pressure/something coming down from their vagina or whether they had a visible mass protruding from the vagina in the past one year ([114](#_ENREF_114), [122](#_ENREF_122), [123](#_ENREF_123)). All women who have the above symptom will be invited for pelvic examination to confirm the prolapse. To measure the test-retest reliability, 35 women who will complete the questionnaire two weeks later will be purposefully selected based on their accessibility after two weeks. Then all women will be examined and the stage of prolapse will be classified according to the standard Pelvic Organ Prolapse Quantification system.

## Statistical Analysis

The data will be entered into Epidata version 3.1 and analyzed using STATA version 16 software. After cleaning and coding the data, sociodemographic characteristics and clinical information will be described by frequency, percentage, mean and standard error, or median and inter-quartile range based on normal distribution of the data.

**Content validity**: whether the questionnaire could be understood by the patients and expert, and whether all important and relevant items had been included by the expert panel will be evaluated. Face validity, the extent to which the questionnaire is a measure of what it is intended to measure in the opinion of patient and expertise will be evaluated by the expert committee through the adaptation process ([124](#_ENREF_124)). Experts will evaluate the comprehensiveness and the relevance using the scale that range from 1- 4 (1 = not relevant, 2 = somewhat relevant, 3 = quite relevant and 4 = highly relevant) ([125](#_ENREF_125), [126](#_ENREF_126)). Experts’ agreement on relevancy will be calculated using the Content Validity Index (CVI), and agreement ≥ 80% will be considered acceptable ([119](#_ENREF_119), [125](#_ENREF_125)). Moreover, the extent to which the tool is acceptable to participants will be evaluated using the estimated time required to fill out (respond to) the questionnaire, percentage of a fully completed tool, percentage of difficult items, and level of missing data ([127](#_ENREF_127)). The questionnaires’ content validity will be evaluated to ensure that there are only a few missing responses, used the full range of scores with little skew, have few ceiling (best possible score) or floor (poorest possible score). Floor and ceiling effects will be computed by percentage frequencies of the lowest and highest score achieved. Accordingly, ceiling and floor effects are considered presented if > 15% of participants achieved this score ([120](#_ENREF_120)). The presence of ceiling and floor indicates that the tool fails to measure extreme symptoms.

**Construct validity:** Construct validity will be evaluated by explanatory factor analysis using principal component analysis (PCA) and known-group validity. To run the factor analysis, its appropriateness will be determined by Kaiser-Meyer-Olkin (KMO) statistics and Bartlett’s test of sphericity. The value of Kaiser-Meyer-Olkin (KMO) is acceptable if it is > 0.5 ([128](#_ENREF_128)) and Bartlett’s test is acceptable if it is significant, P-value < 0.05 ([129](#_ENREF_129)). To identify the number of meaningful factors, the Scree plot representing eigenvalues associated with each factor will be used. Factors with eigenvalues > 1 will be considered meaningful and retained for rotation ([130](#_ENREF_130)). In addition, to estimate factor correlations, the varimax orthogonal rotation procedure will be used, and communalities and factor loadings ≥ 0.4 will be considered sufficient ([131](#_ENREF_131)). Items with factor loading ≥ 0.4 on more than one factor will be considered to be cross-loading ([128](#_ENREF_128)). To evaluate the known-group validity, the median difference of POP-SS values among the four stages of POP as classified by the POP-Q system will be compared by using the Kruskal-Wallis test.

**Criterion Validity:** How well the questionnaire correlates with an existing gold standard, will be evaluated by comparing the POP-SS score with the stages of prolapse confirmed through pelvic examination. Spearman’s correlation coefficient (SCC) and Kruskal-Wallis test will be used for statistical analysis. The size of the correlation coefficient will be interpreted as: 0.8 – 1.0 excellent, 0.6 - 0.8 very good, 0.41 – 0.6 good, 0.21-0.4 sufficient and 0.00-0.2 poor ([132](#_ENREF_132)).

**Reliability test:** Questionnaire reliability will be assessed using agreement and consistency indices. Cronbach alpha between 0.7 and 0.95 ([120](#_ENREF_120)) will be considered adequate for the internal consistency of the POP-SS questionnaire. Each item’s reliability will be analyzed by assessing its item-total correlation and the overall reliability if a specific item is deleted. Item-total correlation of ≥ 0.5 and inter-item correlation of ≥ 0.3 will be considered adequate ([133](#_ENREF_133)).

Test-retest reliability will be computed based on the Intra-class Correlation Coefficient (ICC). Single rating, absolute agreement, and a two-way mixed-effect model will be used and an Intra-class Correlation Coefficients (ICC) of ≥ 0.7 will be accepted ([120](#_ENREF_120)).

# **PAPER II**

Prevalence and risk factors of Pelvic Organ Prolapse among women in Sidama Region, Ethiopia; 2022**.**

## Specific Objectives

1. To determine the prevalence of Pelvic Organ Prolapse among women in Sidama National Regional State, Ethiopia
2. To identify risk factors associated with Pelvic Organ Prolapse among women in Sidama National Regional State, Ethiopia

## Study Setting and Period

This study will be conducted in the Dale and Wonsho districts of Sidama National Regional State, Ethiopia from 01 June – 24 July 2022. Both woredas are known for their highly dense population and coffee production. Dale district (woreda) has 10 health centers and 33 health posts. Hamlin Fistula Center, which provides care for fistula cases and pelvic organ prolapse is found in Yirga-Alem town in Dale district. Wonsho district has five health centers and 17 health posts. According to the 2021 report of Sidama Regional Health Bureau, the total population of Dale district is 254,653 and that of Wonsho district is 129,730 ([134](#_ENREF_134)). According to the Ethiopian Central Statistical Agency, the female population accounts for 49.7% of Dale woreda population and 49.2% of Wonsho population ([135](#_ENREF_135)). Dale district has 36 rural and two urban kebeles while Wonsho district has 17 rural and one urban kebeles (smallest administrative units in Ethiopia).

In 2017, Hawassa University has established its own Health and Demographic Surveillance System site in Dale and Wonsho districts of Sidama Region. The HDSS site include 10 rural kebeles and two urban kebeles. The Surveillance site is called Dale-Wonsho Health and Demographic Surveillance Site (D-W HDSS). The Map of the HDSS site is obtained from previous study (Figure 2).


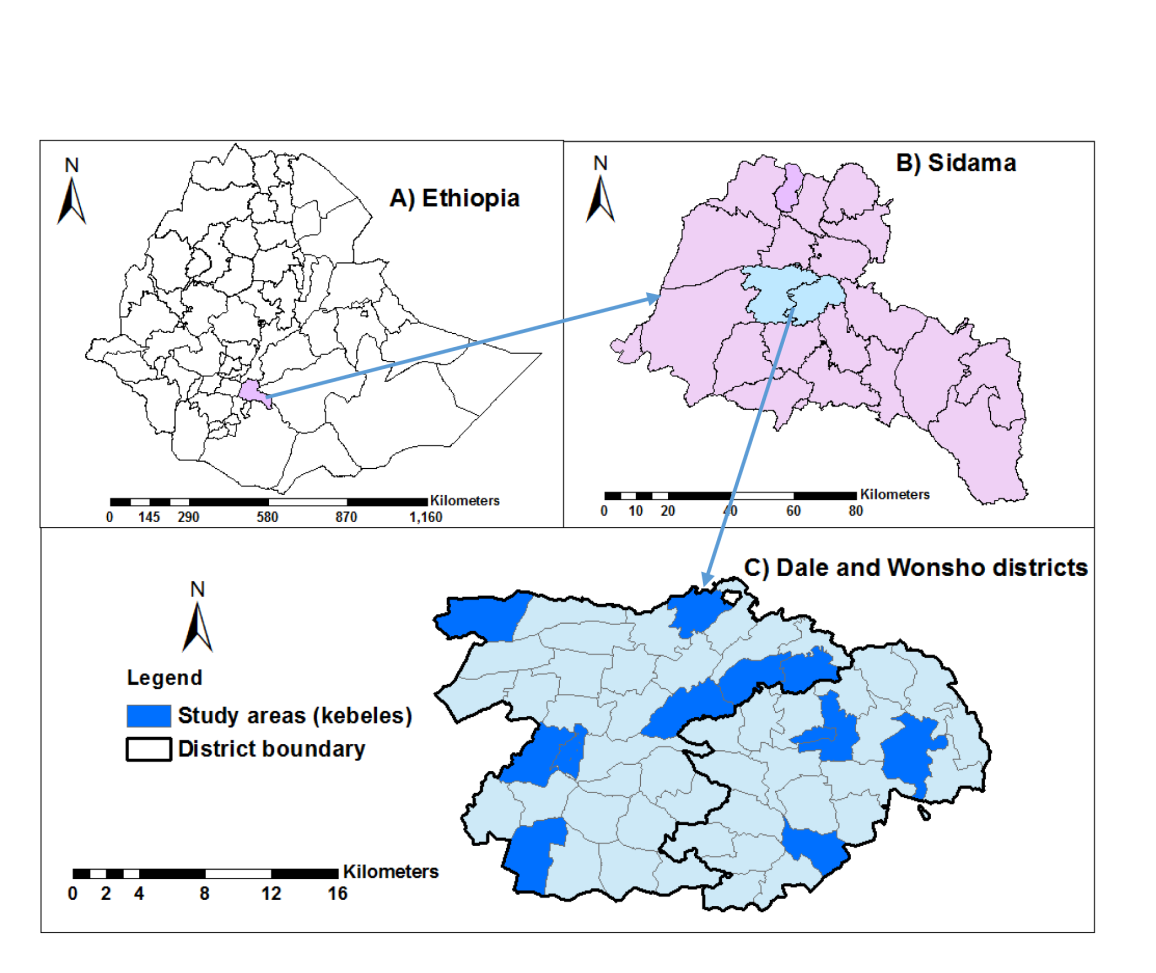


Figure 2: Map of Hawassa University Health and Demographic Surveillance System Sites from Dale and Wonsho Districts ([136](#_ENREF_136)).

.

## Study Design

A community-based cross-sectional survey design will be used to determine the prevalence of pelvic organ prolapse among women in the Sidama region. In a survey study, data are collected either from the whole population or a subset of the population to answer the research question. These studies are useful to assess the burden of disease and inform the planning and allocation of resources. A survey study should be representative of the population to have valid generalization ([137](#_ENREF_137)).

## Study Population

All women who have given birth (at least once) or ≥ 18 years old, and permanent residents (≥ 6months) of the Health and Demographic Survey Site of Hawassa University will be included in this study.

**Inclusion Criteria:** Women whose household information is found in the Health and Demographic Survey of Hawassa University and who have given birth or ≥ 18 years old will be included.

**Exclusion criteria:** Women who are pregnant (confirmed or suspected), in the postpartum period (six weeks), never practiced sexual intercourse, or severely sick during the data collection period will be excluded.

## Sample Size Determination

The sample size was calculated using Open-Epi version 3.1 both for the prevalence of the POP and its associated risk factors. The sample size for the pelvic organ prolapse prevalence study was calculated based on the prevalence of pelvic organ prolapse of 56.3%, a non-response rate of 7% from previous similar study([39](#_ENREF_39)), with the assumption of a 95% confidence interval, and a design effect of 2, the final sample size is 813.

The sample size was also calculated for risk factors from a previous similar studies conducted in the northern part of Ethiopia ([39](#_ENREF_39)) by using Open-Epi version 3.1. For each variable significantly associated with POP, the sample size was calculated with the assumption of 95% confidence level, 80% power, unexposed to exposed ratio of one to one, proportion of outcomes among unexposed categories, and their respective odds ratios. Finally, a design effect of two and non-response rate of 7% were used to calculate the final sample size (Table 1).

Table 1: Sample size calculation based on risk factors of Pelvic Organ Prolapse for the study to be conducted in Sidama National Regional State, Ethiopia, 2022.

| Exposure Variables | | Confidence level | Power | % with outcome | Odds ratio | Design effect | % non-Response rate | Sample size |
| --- | --- | --- | --- | --- | --- | --- | --- | --- |
| Women age | ≤34 | 95% | 80 | 17.58 | Reference | | | |
|  | 35-44 | 95% | 80 | 49.15 | 2.21 | 2 | 7 | 659 |
|  | >45 | 95% | 80 | 29.970.1 | 3.57 | 2 | 7 | 246 |
| Parity | 0 | 95% | 80 | 11.6 | Reference | | | |
|  | 1 | 95% | 80 | 21.15 | 3.16 | 2 | 7 | 396 |
|  | 2 | 95% | 80 | 49.4 | 6.61 | 2 | 7 | 134 |
|  | 3 | 95% | 80 | 59.9 | 8.86 | 2 | 7 | 99 |
|  | 4+ | 95% | 80 | 72.5 | 11.73 | 2 | 7 | 78 |
| Hours of carrying heavy objectives | ≤1 | 95 | 80 | 25.1 | Reference | | | |
|  | 2 | 95% | 80 | 62.5 | 4.34 | 2 | 7 | 160 |
|  | 3 | 95% | 80 | 76.32 | 9 | 2 | 7 | 82 |
|  | 4+ | 95% | 80 | 64.8 | 5.1 | 2 | 7 | 134 |
| Age at last delivery | >36 | 95% | 80 | 77.1 | 2.7 | 2 | 7 | 344 |
|  | ≤36 | 95 | 80 | 51.9 | Reference | | | |

The sample size calculated for the prevalence study produced a larger sample size, 813, that will be used in this study.

## Sampling Techniques

A complex sample survey will be used to recruit study participants. A survey sampling is a process of selecting representative samples from the population and measuring the selected samples to get evidence about population parameters ([138](#_ENREF_138)). If the selection of study participants passes through a series of stages like multilevel sampling, cluster sampling, or stratification, it is called complex sampling. The features of large survey sampling are stratification, clustering, and unequal probability sampling ([138-141](#_ENREF_138)).

The current planned study will be conducted in the Dale and Wonsho districts of the Sidama region. The two districts were selected due to the accessibility of the sampling frame from the Health and Demographic Surveillance System (DHSS) of Hawassa University. The DHSS involved a total of seven rural kebeles (Wayicho, Hida Kality, Shoye, Dagiya, Magara, Danshe sire and Gidamo); and one urban kebeles (Aposto) from Dale district, and three rural kebeles (Mamana, Bokaso and Gishire); and one urban kebele (Wonsho 01) from the Wonsho district. The list of households from these kebeles will be used for the sampling frame in the current planned study.

In this study, Hawassa University Health and Demographic Surveillance System (HDSS) site will be initially stratified into two districts (Dale and Wonsho). Then each district will be re-stratified into urban and rural kebeles. Since there are only one urban kebeles included in to the DHSS from each district, both kebeles will be selected to ensure representativeness. From rural kebeles, representative kebeles will be selected from both districts (four rural kebeles from Dale and two rural kebeles from Wonsho district) based on their selection probability. If the selected rural kebeles (clusters) are adjacent to each other, the second cluster will be replaced by the next kebele (cluster) to create a buffer zone. Finally, equal number, 101.6 approximated to 102, eligible women from each kebele (clusters) will be selected using computer-generated random numbers. This approximation will adjust the sample size to 816 to sample equal number of participants from each clusters. Therefore, this study includes four strata: Dale-Rural, Dale-Urban, Wonsho-Rural, and Wonsho-Urban; eight clusters (kebeles) and 816 eligible women (Figure 3). If there is more than one eligible woman in the selected household, one of them will be selected by the lottery method.

Dale rural (7) () kebele

816 women will be invited to a nearby health facility

Wonsho rural (3) kebele

Select 4kebeles women

Select 2 kebele

Dale Urban 1

Wonsho Urban 1

102 women selected kebeles kebele selected

408 women

102 women selected kebeles kebele selected

204 women selected eles kebele selected

Dale district 8kebeles

Wonsho district 04 kebeles

Select 1kebele women

Select 1kebele women

Dale-Wonsho Health and Demographic Survey Site

**Stratified**

**Stratified**

**Selection probability**

**SRS**

Figure 3: Flow diagram of sampling procedure for the study conducted on Epidemiology of Pelvic Organ Prolapse in Sidama National Regional State, Ethiopia, 2022.

## Data Collection Tools and Procedures

The subjective data (self-reported POP symptom) will be collected using a structured, locally validated questionnaire, and the objective data will be collected by measuring the degree of prolapse in centimeters. The data collection tool has 10 parts (sections). Part-I is about socio-demographic characteristics of the participants’, which has eight items. Part-II is focused on the POP-related lifestyle of the participants’, which has six items. Part-III contains 14 items that ask about the Obstetrics and gynecologic history of the participants. Part-IV is about the wealth index of the study participants’ household which has 28 items. Part-V is about POP-SS, which has nine items for baseline assessment and one additional question for end-line assessment that relates to participants’ perceived change of POP-SS after the intervention (PFMT). Part-VI is about the general Pelvic Floor Disability Index, which has three main components and a total of 20 items. Part-VI asks about involuntary leakage of urine, which has four items. Part-VIII is about Obstetric Fistula, which has three items. Part-IX is about objectively measured POP and related measures, which have 14 measurements, of which nine points were measured from in and around the vagina to diagnose POP. The last part (part-X) is about prolapse-related quality of life, which has a total of 20 items divided into nine domains.

The questionnaires used to collect data about socio-demographic characteristics, lifestyle, obstetrics, and gynecologic history were developed from a review of related literature ([23](#_ENREF_23), [26](#_ENREF_26), [37](#_ENREF_37), [39](#_ENREF_39), [55](#_ENREF_55), [61](#_ENREF_61), [62](#_ENREF_62), [79](#_ENREF_79)). It was initially developed in the English version and will be translated into the local language (Sidaamu Afoo) by a native speaker of the language and an expert in maternal or reproductive health. The wealth index questionnaire was adopted from the Ethiopian Demographic and Health Survey in English version ([142](#_ENREF_142)). The tool used to measure Pelvic Organ Prolapse Symptom is a standard tool validated in English, Turkish, Chinese, and Amharic Version ([16](#_ENREF_16), [113-115](#_ENREF_113)). It will be translated into Sidaamu Afoo and evaluated for its psychometric properties (Paper-I) and then the final version will be used. The POP-SS has seven questions with a five-point Likert scale which ranges from zero to four (0= never felt symptom, 1= occasionally, 2= sometimes, 3= most of the time, and 4= all of the time), a higher score indicating severe symptoms ([16](#_ENREF_16)). The participants will be asked how often they have had the following symptoms in the last four weeks: 1) A feeling of something coming down from the vagina? 2) Uncomfortable feeling in the vagina that worsens when standing? 3) A heaviness feeling in the lower abdomen? 4) A dragging feeling in the lower back? 5) A difficulty of emptying the bladder? 6) A feeling of incomplete bladder emptying? 7) A feeling of incomplete bowel emptying? The total score will be calculated by summing up all responses to the seven-question test, and the total score will range from 0 to 28.

Other pelvic floor disorders, including bladder and bowel symptoms, will be assessed using the International Consultation of Incontinence Questionnaire-Short Form (ICIQ-SF) ([143](#_ENREF_143)) and the Pelvic Floor Disability Index (PFDI-20) ([144](#_ENREF_144)). The International Consultation of Incontinence Questionnaire-Short Form (ICIQ-SF) is a four-item, disease-specific tool that assesses the symptoms and quality of life of patients with urinary incontinence. The four items are pertaining to the frequency of leakage, amount of leakage, interference with everyday life, and the perceived cause of leakage. Each item has its own response scale, and the total score is calculated as the sum of the first three items (relating to frequency, volume of leakage, and interference with everyday life). It is treated as a continuous scale with a possible range from 0 to 21, with the higher scores indicating worse symptoms ([87](#_ENREF_87)). The Pelvic Floor Disability Index (PFDI-20) consists of 20 items that contain three subscales: Pelvic Organ Prolapse Distress Inventory-6 (POPDI-6), Urinary Distress Inventory-6 (UDI-6), and Colorectal-Anal Distress Inventory-8 (CRADI-8). Each item has a response of either no (0) or yes (1) depending on the absence or presence of a complaint. If answered yes, the level of discomfort will be rated as 1 = unimportant, 2 = a little, 3 = moderate, or 4 = a lot. Both of these tools will be translated into the local language and translated back to English to check their consistency. The participants will be asked how often they have had the symptoms in the last three months.

To diagnose and quantify the stage of the prolapse objectively, a ruler marked with a centimeter will be used to measure the prolapse of nine points in and around the vagina. In addition, a weight scale and meter marked in centimeters will be used to measure a women’s weight and height to know her Body Mass Index.

Prolapse Quality of Life questionnaires will be used to assess the quality of life among women who have a POP (diagnosed objectively). Health-related quality of life among patients with POP will be measured using the disease-specific tool called Prolapse-Quality of Life (P-QoL) ([90](#_ENREF_90)). It was initially developed in 2005 to measure the severity of prolapse symptoms, its impact on patients’ health-related quality of life, and treatment outcome in English-speaking women. It is prolapse specific, a multidimensional tool with nine domains and 20 items. The domains include general health perception (GHP), prolapse impact (PI), role (RL), physical limitation (PL), social limitation (SL), personal relationship (PR), emotional disturbance (EMO), sleep/energy disturbance (SE), and severity measures (SM). Each domain has its own items/questionnaire each containing four points (0= none/not at all, 1= slightly or little, 2= moderately, and 3= a lot). Each domain has a score of 0 to 100, a higher score indicating poor quality of life. This tool was translated and validated in the Amharic version ([145](#_ENREF_145)). The Amharic version will be translated to Sidaamu Afoo and back-translated to Amharic to check its consistency before actual data collection.

Subjective data will be collected by seven data collectors who were fluent in Sidaamu Afoo, experienced in data collection, and recruited by Hawassa University to collect data from HDSS sites. The data collectors will go to the selected households and collect the data through face-to-face interviews with eligible women (one woman who gave birth at least once or was older than 18 years) from April to June 2022. The interview is mainly focused on socio-economy and demography, obstetrics and gynecology history, wealth index, and symptoms of Pelvic Organ Prolapse. At the end of the interview, all study participants will be invited for a pelvic examination at a nearby health facility; and given an appointment (date and time) irrespective of their symptoms.

Pelvic examinations will be performed by trained staff of Yirgalem Hamlin Fistula Center. On each appointment date, this staff will be assigned to each selected health facility (either health center or hospital) and perform a pelvic examination to diagnose POP by using the Pelvic Organ Prolapse Quantification system ([6](#_ENREF_6)). To perform a pelvic examination, the woman will be informed about the procedure, empty her bladder and bowel. The patient can be positioned in lithotomy position or left lateral. A disarticulated speculum will be inserted into the vagina; and to observe the anterior vaginal wall prolapse, the posterior vaginal wall will be retracted. To observe the posterior vaginal wall prolapse the anterior wall will be retracted during straining or coughing or Valsalva. The descent of the cervix will be evaluated by placing Sims or disarticulated speculum in the vagina and directly observing its descent during Valsalva ([12](#_ENREF_12)).

## Study Variables

The outcome variable of this study is pelvic organ prolapse. The independent variables include: Age in years, mode of delivery, number of childbirth, level of education, body mass index, history of pelvic surgery, trauma, marital status, occupation, heavy lifting, smoking, age at first marriage, age at first delivery, place of delivery, history of instrumental delivery, history of a length of labor more than 24hrs (one day), medical conditions (chronic cough, Diabetes mellitus, hypertension), family history of POP, ethnicity, duration menopause.

## Data Source and Measurement

Data on socio-demography (age, level of education, occupation, permanent place of residence religion, and ethnicity), obstetric and gynecologic history (number of childbirth, age at first marriage and childbirth, mode of delivery) will be recorded based on participants’ self-response to each question.

To diagnose POP symptomatically, the total score of the seven items with a five-point Likert scale will be calculated by summing up all responses and the total score will range from 0 to 28. If the participants' total response score is different from zero, she will be considered as having symptoms of prolapse. The Pelvic Floor Disability Index (PFDI-20) will be measured for each component separately. The average score of each subscale will be computed and multiplied by 25 to convert each subscale score between zero and 100. The overall scale score of the three-component will range from zero to 300. The higher values indicate the worst prolapse symptoms ([144](#_ENREF_144)).

To objectively diagnose POP, there are nine measurement points in the POP-Q system. All are measured at maximum Valsalva, except Total Vaginal Length (TVL) which is measured at rest. Measurements are expressed in centimeters to the nearest 0.5cm. These measures are translated into a staging system as described below (Table 2) ([6](#_ENREF_6), [12](#_ENREF_12), [123](#_ENREF_123)). The nine measurement points in and around the vagina are: Genital Haitus (GH) measured from the middle of the external urethral meatus to the posterior aspect of the hymenal remnant, perineal body (PB) measured from the posterior aspect of the hymenal remnant to the middle of the anal opening, point C is the lowest part of cervix and point D is the insertion of uterosacral ligaments to the cervix. Both points are measured relative to the plane of hymenal remnants. A hymenal remnant is chosen as it is a clearly defined and easily identifiable structure. Total vaginal length is measured from Hymenal remnant to point D (Point C will be used in post-hysterectomy patients). Point Aa is an arbitrary fixed point on the anterior vaginal wall, which is 3cm back from the middle of the external urethral meatus in normal cases. Point Ba is the lowest part of the upper anterior vagina. This point is not a fixed point like Aa. It can be anywhere along the vaginal wall above the first 3cm (Aa). Point Ap and Bp are similar points to Aa and Ba but found on the posterior vaginal wall.

.

Table 2:Stage of Pelvic Organ Prolapse classified according to the Standardized Pelvic Organ Quantification System ([6](#_ENREF_6), [123](#_ENREF_123))

| Stage 0 | Points Aa, Ba, Ap, Bp are all at -3cm and C and D are at greater than /equal to total vaginal length (TVL) -2 cm. |
| --- | --- |
| Stage 1 | The Criteria for stage zero are not met and the leading edge of the prolapse is greater than 1 cm above the hymen. |
| Stage2 | The leading edge of the prolapse is between 1 cm above or below the hymen |
| Stage 3 | The leading edge is greater than 1cm beyond the hymen but less than total vaginal length (TVL) – 2 cm from the hymen |
| Stage 4 | The leading edge of the prolapse is greater than total vaginal length (TVL) -2 cm beyond the hymenal remnant. |

A woman is considered to have POP if she has any stage of prolapse different from stage 0.

Body mass index will be measured at health facilities at the time of pelvic examinations. Participants’ weight will be measured by the kilogram and the height will be measured in meters. Finally categorized as underweight (<18.5Kg/m^2^), normal (18.5 – 25.5Kg/m^2^), overweight (25.5 - 29.9kg/m^2^) and obesity (≥ 30Kg/m^2^) ([146](#_ENREF_146)).

Heavy lifting will be measured by the estimated average hours per day the women carry heavy objects like water, big baby, wood, stone, or types of cement.

History of Pelvic surgery includes any type of surgery that involved pelvic muscle like episiotomy, hysterectomy, or surgery for pelvic tumours.

## Data Quality Assurance

A two-day training will be given for data collectors about the study objectives, tools, and data collection techniques. The questionnaire used to collect socio- demography data was developed in English from a review of the literature and will be translated to the local language. The POP-SS tool will be translated to the local language Sidaamu Afoo and validated among women diagnosed to have POP at Adare, Bensa, and Yirgalem General Hospitals before the data collection. To ensure clarity of the tools other than the POP-SS, it will be pretested on 5% (41) of the sample at Shebadino woreda. The tool will be checked for clarity and time taken to interview a woman. Based on the result of the pre-test, unclear items will be modified to make it clear. The data collection procedures will be closely supervised (daily).

The professionals who perform pelvic examinations will be trained by urogynaecologists to avoid discrepancies across examiners. Furthermore, they will be supervised during data collection (pelvic examination). To reduce the risk of possible bias in complex sampling data, initially, the sampling procedures are clearly stated in the document and will be implemented accordingly to reduce the risk of sampling bias. Both subjective and objective data will be collected using standardized and locally validated tools. The pelvic examiners will be blinded about the POP-SS and background information of study participants.

## Data Management and Analysis

Data will be entered into Epidata version 3.1 and exported to STATA version 16 for analysis. The entered data will be cleaned and recoded before analysis. If there is any missing data, multiple imputation methods will be used to manage the missing data.

Before performing the main analysis, data will be weighted using the sample selection probability and the weighting class adjustment method to control for selection bias and non-response bias respectively ([147](#_ENREF_147), [148](#_ENREF_148)) based on the auxiliary data collected for the Demographic and Health Survey by Hawassa University.

The nature of the data and its distribution will be checked to identify any special distribution. For categorical data, its frequency will be run and checked for any unusual distribution. For continuous data, both statistical methods (mean, standard deviation, median, Skewness, and kurtosis); and graphical presentations (histogram) will be used to check its normal distribution. A mean and standard deviation will be reported for normally distributed data, while the median and interquartile ranges will be reported for non-normally distributed data.

The primary outcome in this study is the presence of anatomical pelvic organ prolapse, which is pelvic organ prolapse of stage I or more prolapse diagnosed through pelvic examination.

To identify predictors of POP, initially, data stratification will be done to identify possible confounders and effect modifiers ([149](#_ENREF_149)). Variable are considered confounding when their crude result (unadjusted odds ratio) is different from Mantel-Haenszel (M-H) (adjusted odds ratio) on stratified data ([150](#_ENREF_150)). An effect modifier is considered when the odds ratio of a variable is different across the strata.

In addition, the multicollinarity of the independent variables will be checked before the variable is fitted into multivariable logistic regression. In the multiple linear regression model, the variance inflation factor (VIF) > 1 and tolerance statistics < 0.1 show the presence of multicollinarity. The variance inflation factor (VIF) of 1-5 shows moderate multicollinarity, while VIF >5 shows high multicollinarity. In this analysis, variables with high multicollinarity will be omitted from the analysis ([151](#_ENREF_151)).

Finally, bi-variable and multivariable logistic regression analysis will be done using complex sample survey analysis to control for possible confounding and identify predictor factors ([149](#_ENREF_149)). In bi-variable logistic regression analysis, variables with a P-value < 0.25 will be fitted into multivariable logistic regression in a stepwise fashion ([152](#_ENREF_152)) using the ENTER method. In multivariable logistic regression, a variable is identified as a predictor factor for POP if the 95% confidence interval of the adjusted odds ratio does not include 1 and the P-value <0.05. The model parameter is estimated by pseudo maximum likely hood estimation that accounts for the features of a complex sample design. A Archer and Lemeshow goodness-of-fit test that accounts for the effect of sampling design and weight will be used also ([153](#_ENREF_153)).

The Principal Component Analysis (PCA) will be carried out for the computation of the wealth index. The PCA will be done in a series of steps. Initially, the continuous variables will be standardized so that each one of them contributes equally to the analysis. Then covariance matrix computation will be done to identify correlations and compute the eigenvectors and eigenvalues of the covariance matrix to identify the principal components (new variables). Then a feature vector will be created to decide which principal components to keep. Finally, recast the data along the principal component axes ([154-156](#_ENREF_154)). All the variables are dichotomized and the scores are derived using principal component analysis and ranked in three quintiles ([142](#_ENREF_142)). Lowest quintile: those who score below the 3^rd^ quintile in wealth index ranking. Middle quintile: those who score the middle 3^rd^ quintile in wealth index ranking. Highest quintile: those who score the upper 3^rd^ quintile in wealth index ranking.

# **PHASE-II PROJECT**

# **PAPER-III**

Effect of Pelvic Floor Muscle Training for reducing Pelvic Organ Prolapse Symptoms in Sidama National Regional State, Ethiopia, 2022.

## Objective

1. To evaluate the effect of Pelvic Floor Muscle Training for reducing prolapse symptoms in Sidama Region, Ethiopia.

## Study Area and Period

The study area is the same as for paper II. Study time will follow immediately after study I and continued for six months. Therefore, this study will be conducted from July – to December 2022.

## Study Design

A Cluster Randomized Control Trial Study will be conducted. The clusters represent enumeration areas in each kebele.

## Study Subjects

The study participants of this study will be women who are diagnosed to have symptoms of POP and confirmed POP stage I-III through a pelvic examination on paper II of this dissertation proposal.

## Eligibility Criteria

**Inclusion**: Women who will be diagnosed to have symptoms of POP as diagnosed by POP-SS and confirmed POP stage I-III on pelvic examination.

**Exclusion** **Criteria**: Women who were diagnosed to have symptomatic Pelvic Organ Prolapse stage I-III but planned to leave the study area within six months (study period), severely ill women, women with psychiatry disorders, women with delivered myoma, or women who want to have prolapse surgery within six months will be excluded.

## Sample Size Determination

Sample size calculations were performed using Open-Epi version 3.1, based on the following assumptions: a 99% confidence level, 80% power, an exposed-to-unexposed ratio of 1, a POP-SS mean difference of 3.16 with a standard deviation of 4.78 in the intervention group, and a mean difference of 0.12 with a standard deviation of 3.86 in the control group over six months (14). Considering a 16% (15) refusal and loss to follow-up, the effective sample size (individual-based randomization) is 113.4. The decision to adjust the confidence level to 99% was based on the adjusted level of significance to 0.0125 than the usual p-value of 0.05 to account for multiple comparisons across various outcomes (16) (POP-SS and three distinct domains of P-QoL).

The minimum number of clusters needed is calculated by multiplying the effective sample size by the Intra-Cluster Correlation Coefficient (ICC) factors for both groups (17). The estimated fairly typical value of the Intra-Cluster Correlation (ICC) factor is 0.03 (17-19). Accordingly, the minimum required cluster number is 1.67 for each group (intervention and control group). However, to ensure the adequacy of the cluster and to have good power (20, 21), eight clusters (kebeles) will be included in this study. To account for the cluster effect, the effective sample size is multiplied by a Variance Inflation Factor (VIF) of 1.35. The Variance Inflation Factor (VIF) is calculated with the assumption of an equal cluster size. Using Intra-Cluster Correlation value of 0.03, (VIF = 1+ ((n-1) ICC)), where ‘n’ is the average cluster size (17-19). Accordingly, the adjusted sample size is 155 with 19.4 participants per cluster.

## Randomization

The study site, Hawassa University Demographic and Health Survey sites, comprises eight kebeles from Dale district and four kebeles from Wonsho district. In this study, these kebeles will be stratified into four strata based on their geographical location (Dale-Urban, Dale-Rural, Wonsho-Urban, and Wonsho-Rural). Stratifying on location reduces stratum variability and helps to ensure that they are balanced across study arms (20, 21). To improve similarity across treatment and control groups, the same number of clusters from each strata will be assigned to each arm. Accordingly, four clusters/kebeles from Rural-Dale will be assigned to each arm by lottery method. Similarly, two kebeles/clusters from rural-Wonsho will be assigned to each arm. The two urban kebeles will be assigned to each arm by lottery method. To reduce the risk of information contamination, the interventional and control clusters will be separated by a buffer zone (20), at least one kebele (Figure 4).

**Dale-Wonsho DHSS = 12 kebeles**

Wonsho-Rural (3) kebele

Dale-Rural (7) () kebele

Dale-Urban 1

Wonsho-Urban 1

Select 2 kebele

Select 1kebele women

Select 1kebele women

Select 4 kebeles women

1kebele

1 kebele

2kebele

2kebele

Intervention group (cluster=4, n= 78)

Control group (cluster=4, n= 78)

Received counselling & Training (n= -)

Received Counselling (n= -)

Analyzed at six months (n= -)

Analyzed at six months (n= -)

Loss to follow-up (n= -)

Loss to follow-up (n= -)

Dale-district 8 kebeles

Wonsho district 4 kebeles

1kebele

1kebele

**Stratified**

**Stratified**

**Selection** **Probability**

**SR**S

**SRS**

Figure 1: Flow diagram for the cluster randomized control trial study on Effect of Pelvic Muscle Training on Prolapse symptom in Sidama Region, Ethiopia, 2022.

## Description of Intervention

From each selected health facility, two female Nurses or Midwives will be trained on Pelvic Floor Muscle Training and how to counsel patients. The training will be given for nurses and midwives by urogynaecologist. The training will have a theory, simulation, and video aids.

Among women diagnosed to have symptomatic stage I-III pelvic organ prolapse, 136 will be assigned to the interventional and control groups based on their pre-assigned enumeration area (cluster). Those assigned to intervention will receive pelvic floor muscle training and prolapse lifestyle advice focusing on constipation, chronic coughing, heavy lifting, and other medical conditions. The control group will receive only lifestyle modification but not trained on pelvic floor muscle exercise. The detailed counselling will be provided using a translated Sidaamu Afoo version of a leaflet (Annex-IV) (22).

In the interventional group, at the initial visit, women will be informed about their stage of POP, type of prolapse (anterior, posterior, or central), the advantage of pelvic floor muscle exercise, and how it works. The detail of the exercise and follow-up schedule will be explained in detail in a private room for 30-40minutes. They will be trained to sit or lie comfortably, train pelvic muscles with 10 times 10-second maximum holds, and 10 fast contractions. This will be repeated at least three times per day (morning, afternoon, and night) (14, 22). They will be invited to attend five visits (at weeks 0, 2, 6, 10, and 15) at a nearby health facility. Five visits are preferred because 15-weeks intensive training is recommended to get adequate muscle strength) (23).

At the initial visit, they will be given a diary where they record (tally) the duration and frequency of exercise they practiced. In case they cannot make a tally, a different color and size of buttons will be provided (larger black buttons represent a number of days per week they perform the training, medium red buttons represent the frequency of training performed per day and the white small buttons represent the number of contractions performed). They will put buttons in a given box each day they perform the training and this will be checked on each visit. To ensure participants' follow-up, the respective pelvic muscle trainer will remind them two days before the appointment day through women’s phone or their respective Health Extension workers. In addition, transportation costs and time compensation will be covered by the research team. In a case where the participant cannot attend the visit, the PFM trainer will visit her at home. The change in prolapse symptoms will be analyzed at six months.

## Variables

The outcome variable of this study is a change in Pelvic Organ Prolapse Symptom as measured by POP-SS and the intervention is Pelvic Muscle Training. Covariates include degree of POP, the severity of POP-SS, adherence to Pelvic Floor Muscle Training protocol, maternal age, location of POP, history of pelvic surgery, number of childbirth, level of education, body mass index, trauma, marital status, occupation, age at first marriage, age at first delivery, place of delivery, history of prolonged labor, medical conditions (chronic cough, diabetes mellitus), family history of POP, mode of delivery, duration since last menstrual period.

## Data Measurements

At each visit, women's adherence to pelvic floor muscle training will be evaluated by their pelvic muscle trainer. At week two, women will be assessed for adherence to pelvic floor muscle exercise in the last two weeks (three times a day, once a day, a more than once per week, once per week, a more than one days per week). At weeks six, 10, and 15, they will be assessed for adherence to pelvic floor muscle exercise in the last four weeks using similar questions used on week two (three times a day, once a day, a more than once per week, once per week, a more than one day per week). In addition, they will be assessed for prolapse symptoms at weeks 10 and 15.

At the end of six months, the overall adherence to training protocol will be evaluated from their dairy or report of pelvic muscle trainer. Women’s adherence to training protocol is considered adequate when they complete at least 80% of the training sessions (24). This will be calculated at each visit based on the training session and finally compiled.

The primary outcome of the study is a change in prolapse symptoms measured by POP-SS. The POP-SS is the same measurement with study one which measures the frequency of prolapse symptoms in the last four weeks, measured by seven items with a Likert-scale of 0 to 4 scale (0= never felt symptom, 1= occasionally, 2= sometimes, 3= most of the time and 4= all of the time). The total score will be calculated by summing up all responses for the seven-question and the total score will range from 0 to 28, the highest value indicating the severity of prolapse symptoms (25, 26). At the end of the six months, the mean change in prolapse symptoms from the initial visit will be compared between the two groups (intervention and control groups) to measure the effect of pelvic muscle training.

The secondary outcome, women’s perceived change in prolapse symptom since the start of the intervention, will be measured as improved, the same, or worsened based on Patient Global Impression of Change (PGIC) (14, 27).

## Data Collection Tools and Procedure

The Pelvic Organ Prolapse symptoms will be assessed using a translated and validated Sidaamu Afoo version of the POP-SS questionnaire. This tool will be obtained from Paper I of this dissertation project (Paper I). After the intervention, in addition to the POP-SS tool, the Patient Global Impression of Change (PGIC) tool will be used to evaluate patients’ perceived change in prolapse symptoms. The PGIC evaluates overall health status as perceived by the patient in a seven-point single item scale measured as 1= very much improved, 2= much improved, 3= minimally improved, 4 = no change, 5= minimally worse, 6= much worse or 7= very much worse. This scale can be classified into three major categories for description purposes: worsen or disease deterioration (very much worse, much worse, or minimally worse), the same (no change), and improved (minimally improved, much improved, and very much improved) (27). The PGIC will be translated into Sidaamu Afoo by native speakers and translated back to English to check its consistency.

After six months of intervention, the same data collectors of the baseline data will go to the house of the study participants and collect the data through face-to-face interviews. These data collectors are those who were recruited by Hawassa University to collect data from the HDSS site and fluent in Sidaamu Afoo.

## Data Quality Control

To ensure the quality of data, the tool will be translated to the local language and validated according to the protocol prior to this study. Data collectors will be trained for two days and supervised daily.

To reduce the risk of bias, the clusters will be stratified and allocated to each arm to balance both groups. Clusters from each strata will be allocated randomly by using a lottery method. A buffer zone, at least one kebele, will be used to separate the interventional clusters from the control clusters, to reduce information contamination (this will avoid the risk of diluting the effect of treatment). The post-intervention data collectors will remain blinded to the interventional and control groups until the end of the study. To reduce loss to follow-up, participants’ transportation costs and time compensation will be covered by the investigator. In the event that the patients cannot attend the visit, they will be visited at home. Their adherence to the training protocol will be recorded daily and checked at each visit. The completeness of the data will be checked daily by the supervisor; data entry will be started as soon as the data collection process is initiated. If any error is identified while checking for completeness and data entry, the data collector will go back to the specific house and correct the error. Data stratification and multivariable linear regression will be run to control for possible confounders (28). The hard copy will be stored in a private cabinet by the principal investigator.

## Data Management and Analysis

Data entry, cleaning, missing data management and descriptive data presentation is similar with Paper-II. In addition, in this paper, to assess the effect of pelvic floor muscle training on prolapse symptoms, an Intention-to-treat (ITT) analysis will be used at the end of six months of training. This analysis will include all women included in the trial study at the beginning and who attended at least one follow-up. A paired t-test will be used to evaluate the mean change from baseline for both groups (intervention and control) groups. Then independent t-test will be used to compare the mean change for the interventional groups against the mean change of the control group as an effect size. The level of significance will be set at a P-value < 0.0125 by using the Bonferroni correction formula for multiple outcomes (16, 29, 30). In this study, there are four outcomes of this single intervention, which included a mean change in prolapse symptoms and three components of prolapse quality of life (Paper IV). Therefore, the level of significance is obtained by dividing the usual P-value (0.05) by a number of statistics to be performed.

A summary of statistics will be computed both at the cluster-level and individual levels for both arms. The presence of cluster effects will be assessed and a multilevel analysis will be considered if a significant ICC value of > 0.5 is detected (31). As an alternative, since the outcome of this study is continuous data, both the cluster-level and individual-level analysis will produce the same result if the cluster size is equal. But if the cluster size is variable, an individual-level analysis weighted by cluster size will be done (20, 21).

This study will use stratified cluster randomization based on geographical locations. This may not balance all possible confounders, especially individual-level factors that may vary across strata. Therefore, the statistical technique of confounder control will be used to control possible confounders at the analysis stage. A Linear Mixed-Effect Model will be used to control confounders in multivariable linear regression. This analysis can increase the statistical power and preferable than the general equation estimation model (GEE) (32). Multicollinarity of the independent variables will be checked before fitting the variable into multivariable linear regression in similar fashion with Paper-II.

# **PAPER-IV**

Effect of Pelvic Floor Muscle Training to improve Prolapse-Quality of Life in Sidama National Regional State, Ethiopia, 2022.

## Objectives

1. To Evaluate the Effect of Pelvic Floor Muscle Training on Prolapse-Quality of Life in Sidama Region, Ethiopia

## Study Area and Period

The same as for paper III

## Study Design, Study Subjects, Randomization, Sampling Technique and Description of Intervention

The same as for paper III

## Sample Size Determination

This sample size was based on paper III since we were unable to determine the effect of pelvic floor muscle training (PFMT) on quality of life as measured by the Prolapse Quality of Life Questionnaire.

## Data Collection Tools and Procedures

Data about health-related quality of life among patients with POP will be measured using the disease-specific tool called Prolapse-Quality of Life (P-QoL) (33). It was initially developed in 2005 to measure the severity of prolapse symptoms, its impact on patients’ health-related quality of life, and treatment outcome among English-speaking women. It is prolapse specific, a multidimensional tool with nine domains and 20 items. The domains include general health perception (GHP), prolapse impact (PI), role (RL), physical limitation (PL), social limitation (SL), personal relationship (PR), emotional disturbance (EMO), sleep/energy disturbance (SE), and severity measures (SM). Each domain has its own items/questionnaire each containing four points (0= none/not at all, 1= slightly or little, 2= moderately, and 3= a lot). Each domain has a score of 0 to 100, a higher score indicating poor quality of life. This tool was translated to and validated in the Amharic version. The Amharic version had categorized the nine domains into three major components. These components include Physical (physical component/PC; which is composed of general health perception, prolapse impact, physical limitation, social limitation, role, and severity measure), psychological, which includes: emotional disturbance and sleep disturbance) and personal relationship (25). In the current study, the Amharic version will be used after being translated into Sidaamu Afoo and translated back to check for consistency.

In addition, after the intervention, to evaluate patients' overall satisfaction with their post-intervention conditions, Patient Global Impression-Index (PGI-I) tool (27, 34) will be used. This tool measures how patients perceive their improvement after intervention. It contains a single item of a seven-point scale (7=very much worse, 6=much worse, 5=minimally worse, 4 = no change, 3=minimally improved, 2=much improved, and 1=very much improved). This score will be re-categorized as disease deterioration (minimally worse, much worse, or very much worse), no change (the same, remain stable), and disease improved (very much improved, improved, or minimally improved). This tool will be used after translated into the local language (Sidaamu Afoo) and back to English to check for consistency.

Data collection will be conducted in two phases. The baseline data will be collected from 130 symptomatic POP stage I-III participants at the health facility immediately as soon as they are diagnosed with POP (Paper-II). The data will be collected through face-to-face interviews by the same person who perform pelvic examination from 01 June – 24 July 2022. The end-line data will be collected after six months of intervention. The data will be collected at the household level by data collectors who know the house of these participants. These data collectors will be those who collected the data at the beginning of the study (Paper-II).

## Variables

The primary outcome variable of this study is a change in Health-Related Quality of Life (HRQL). The secondary outcome is participants’ perceived change in Prolapse impact on their quality of life measured by Patient Global Impression of Improvement (PGI-I) (34). The intervention is Pelvic Floor Muscle Training and other covariates include degree of POP, the severity of POP-SS, adherence to Pelvic Floor Muscle Training protocol, maternal age, location of POP (anterior, posterior or apical), number of childbirth, level of education, history of depression, need for fertility, body mass index, trauma, marital status, occupation, age at first delivery, place of delivery, history of prolonged labor, medical problem (chronic cough, diabetes mellitus), family history of POP, mode of delivery.

## Data Measurements

Study participants’ adherence to training protocol will be evaluated at each follow-up time (week 2, 6, 10, and 15). At week two, women will be assessed for adherence to pelvic floor muscle exercise in the last two weeks (a few days per week, once per week, a few times a week, once a day, three times a day). At weeks six, 10, and 15, they will be assessed for adherence to pelvic floor muscle exercise in the last four weeks using similar questions used on week two (a few days per week, once per week, a few times a week, once a day, three times a day). In addition, they will be assessed for improvement in prolapse quality of life at 10 and 15 weeks. At the end of six months, the overall adherence to training protocol will be evaluated from their dairy or report of pelvic muscle trainer. Women’s adherence to training protocol is considered adequate when they complete at least 80% of the training sessions (24).

Prolapse quality of life will be measured for each three components of the tool separately. Each component will have a score of zero (0) to 100. The mean change of each component will be evaluated at the end of six months for both arms. The mean change will be compared between the two groups (intervention and control groups) to measure the effect of Pelvic Muscle Training. The secondary outcome, women’s perceived improvement after the intervention will be measured as improved, the same, or worsened based on Patient Global Impression of Improvement (PGI-I).

## Quality Assurance

The same as for paper III. However, the P-QoL tool will be translated but not validated in Sidaamu Afoo.

## Data Management and Analysis

The Data management and analysis procedure is similar with paper-III. In addition to that, a paired t-test will be used to evaluate the mean change of the three components of P-QoL from baseline for both groups (intervention and control) groups. Then an independent t-test will be used to compare the mean change for interventional groups against the mean change of the control group as the effect size for all the three components separately. The level of significance will be set at a P-value < 0.0125 by using the Bonferroni correction formula for multiple outcomes (16, 29, 30).

## Ethical Considerations

This study protocol will undergo review by the Institutional Review Board of Hawassa University at the College of Medicine and Health Sciences. Once it is approved, a support letter will be obtained from Hawassa University College of Medicine and Health Sciences and given to the Sidama National Regional Health Bureau for permission and co-operation. A support and permission letter obtained from Sidama National Regional Health Bureau will be submitted to Dale district and Wonsho district Health Offices. A letter from the district Health Offices will be submitted to their respective health facilities and Kebele administrative included in the study.

To reduce the risk of stigma and discrimination, the baseline data will be collected in a private space (location) at the home of the participants. The leading data collectors will be female data collectors who are fluent in the local language, Sidaamu Afoo, in which the data will be collected. These data collectors will introduce the objective of the study, the data collection process, and how to ensure the privacy and confidentiality of the participants. Finally, written consent will be obtained. Those who cannot read and write can sign their names with their index fingers, stating that the consent was read to them by data collectors. Pelvic examinations will be performed at delivery rooms or gynecology outpatient departments and the physical privacy of participants will be ensured. Similarly, both lifestyle counseling and pelvic floor muscle training will be provided in a private room.

All study information will be confidential. Upon enrolment, subjects will be assigned a code that will be used instead of their name or other personally-identifying information. The key associated with the codes and the subjects’ personally identifying information will be restricted to the principal investigator and study staff. Once the data is collected and entered into the Epidata version 3.1 software, all information will be de-identified and the data analysis will contain only the subject code. All hard study tools will be stored in a locked cabinet with restricted access to others, and it will be controlled by the study investigator. The electronic data will be stored in STATA version 16 software for five years.

The risks to participants in this study are minimal. All questionnaires and study tools are designed in the local language so that participants can easily understand them. There is a small risk of psychological distress in exposing their private organs for a pelvic examination. There is also a risk of temporary discomfort associated with the physical exam performed at each study visit. Moreover, there is a risk of inconvenience related to returning home for follow-up visits. These risks will be minimized during the consent process by allowing participants to cease enrolment at any time. The travel cost and time compensation will be covered by the research team (200 Ethiopian Birr, equivalent to four dollars on average). If the participant cannot attend follow-up visits, the trainer will visit her at home.

Participants with advanced stages of prolapse (stage-IV) will be linked to the Yirgalem Hamlin Fistula Center for further management if the participant is a volunteer. The risks associated with pelvic floor muscle training are not yet reported, so this intervention will not add risk to these subjects

## Trial Registration

Once the proposal is approved and ethical clearance is obtained, it will be registered at ClinicalTrials.gov ([Home - ClinicalTrials.gov](https://clinicaltrials.gov/)).

# **EXPECTED OUTCOME**

This study will have different outcomes. The first paper will produce a translated and validated Sidaamu Afoo version of the POP-SS tool that will be used to screen for disorder in this community. The second paper will determine and show the prevalence of POP and its risk factors in the community of the Sidama region. The effect and feasibility of pelvic floor muscle training in this specific study area will be evaluated. The training is expected to reduce the burden of prolapse symptoms and improve the quality of life among POP cases. If the training is effective in reducing prolapse symptoms and improving quality of life, it will be expanded to other areas. It is expected to reduce the cost of surgical POP management.

# **BENEFICIARIES**

The direct beneficiaries of this study are all women who have anatomical pelvic organ prolapse. Their benefits include free screening for the symptoms and anatomical defects, free and early counselling and training for mild to moderate stages, and a referral system for advanced stages. The secondary beneficiaries of this study include health facilities, the community, and the families of the participants. This intervention will be provided at the early stage of POP, which reduces the risk of surgical management, which in turn reduces the resources and cost both for the family and the health facility. Moreover, we hope that in the long term, this information will provide an evidence-based treatment option for how best to correct prolapse for women in Ethiopia at a low cost.

# **COLLABORATORS**

This project needs the collaboration of different stakeholders to be effectively accomplished. Hawassa University, the home base of research teams, will review the protocol and provide ethical approval and some financial support of 60,000 Ethiopian birr (equivalent to 1,200 dollars). Most of the budget needed will be covered by the University of Minnesota, Maternal Health and the Worldwide Fistula Fund. The gloves and spatula needed to measure prolapse stage will be provided by the Ethiopian Hamlin Fistula Center. The Yirgalem Hamlin Fistula Center and selected health facilities will allow pelvic examination rooms and health professionals needed to examine study participants and provide training on PFMT (Table 3).

Table 3: Potential collaborators and their role for the study on Pelvic Organ Prolapse in Sidama Region, Ethiopia, 2022

|  | **Collaborators** | **Responsibility** |
| --- | --- | --- |
|  | Hawassa University | Provide Ethical Clearance |
|  |  | Provide study participants basic information from the database (HDSS) |
|  |  | Provide 60,000 ETB ($1200) for data collections |
|  |  | Assign senior research advisors |
|  | Sidama region health Bureau | Provide permission and support letter |
|  | Yirgalem Hamlin Fistula Center. | Provide patient pelvic examination site |
|  |  | Provide treatment for advanced-stage Prolapses |
|  |  | Assign and allow time for urogynaecologist to train and supervise the pelvic examination process. |
|  |  | Assign two trained health professionals to perform pelvic examinations. |
|  | Respective health centers | Provide patient pelvic examination site and materials |
|  |  | Assign two female Midwives/Nurses to train and counsel women on PFMT. |
|  | Health Extension workers | Remind and took patients to follow up site (health facility) |
|  |  | Counsel and convince participants to adhere to the training protocol |
|  |  | Support women to record pelvic exercises they performed |
|  | University of Minnesota | Provide financial support |

# **TIME TABLE**

Table 4: Time table indicating activity schedules for the study conducted on Pelvic Organ Prolapse in Sidama Region, Ethiopia, 2022

| Activities | Responsible person | April, 2022 | May, 2022 | Jun, 2022 | July, 2022 | Aug, 2022 | Sept, 2022 | Oct, 2022 | Nov, 2022 | Dec 2022 | Jan 2023 | Feb 2023 |
| --- | --- | --- | --- | --- | --- | --- | --- | --- | --- | --- | --- | --- |
| Ethical clearance | Hawassa University IRB |  |  |  |  |  |  |  |  |  |  |  |
| Preparation for data collection | Research team |  |  |  |  |  |  |  |  |  |  |  |
| Tool validation | Data collectors |  |  |  |  |  |  |  |  |  |  |  |
| Data collection | Research team |  |  |  |  |  |  |  |  |  |  |  |
| Report write up | Research team |  |  |  |  |  |  |  |  |  |  |  |
| **PHASE-II** | | | | | | | | | | | | |
| Pelvic Floor Muscle Training | Women and trainer |  |  |  |  |  |  |  |  |  |  |  |
| End-line Data collection | Data collectors |  |  |  |  |  |  |  |  |  |  |  |
| Data analysis | Research team |  |  |  |  |  |  |  |  |  |  |  |
| Report write up | Research team |  |  |  |  |  |  |  |  |  |  |  |

# **BUDGET**

Table 5: Budget Breakdown for the study conducted on Pe/vic Organ Prolapse in Sidama Region, Ethiopia, 2022.

| S. no | Item | Unit | Quantity | Cost/unit (ETB) | Multiplication | Total in ETB and (American Dollar) |
| --- | --- | --- | --- | --- | --- | --- |
|  | Ethical clearance | Proposal | 01 | 300 |  | 300 ($6) |
|  | Tool translation | Person | 02 | 359 | 5days | 3590($71.8) |
|  | Questionnaire duplication | Page | 20 | 2 | 857 | 34,280($685.6) |
|  | Data collection training | Person | 20 | 359 | 05 days | 35,900 ($718) |
|  | Hall rent | Room | 01 | 1000 | 03 days | 3000 ($60) |
|  | Refreshment | Person | 20 | 150 | 03 days | 9,000 ($180) |
|  | Transportation | Person | 20 | 100 | 2 round | 4000 ($80) |
|  | Trainer perdiem | Person | 02 | 1500 | 03 days | 9000 ($180) |
|  | Data collector perdiam | Person | 16 | 359 | 10 days | 57,440 ($1,149) |
|  | Women transportation | Person | 816 | 300 |  | 244,800 ($4,896) |
|  | Pelvic examiner | Person | 2 | 1500 | 30 days | 90,000 ($1,800) |
|  | Tool validation | Person | 2 | 300 | 50patient | 30,000 ($600) |
|  | Mask and Sanitizer | Person | 15 | 100 |  | 1500 ($30) |
|  | Speculum rent | Each | 816 | 20 |  | 16,320($326.4) |
|  | Sub-total 1 |  |  |  |  | **539,130 ($10,782.6)** |
|  | **PHASE**-II | | | | | |
|  | PFMT trainer | Person | 4 | 1000 | 10 days | 40,000 ($800) |
|  | Follow-up transportation | Person | 136 | 200 | 4 round | 108,800 ($2,176) |
|  | End line assessment | Person | 8 | 359 | 5 days | 12,565 ($251.3) |
|  | Supervisor per diam | Person | 02 | 359 | 40 days | 28,720 ($574.4) |
|  | Data entry (clerk) | Person | 02 | 359 | 10 days | 7,180 ($143.6) |
|  | Mobile card | Person | 15 | 200 |  | 3000 ($60) |
|  | Glove | Pack | 18 | 800 |  | 14400 ($288) |
|  | Spatula | Pack | 09 | 500 |  | 4500 ($90) |
|  | Pen and note pad | Each | 100 | 40 |  | 4,000 ($80) |
|  | Sub-total 2 |  |  |  |  | **223,165 ($4,463.3)** |
|  | Ground total |  |  |  |  | **762, 295 ($15,245.9)** |

****** $1 = 50 Ethiopian birr

# **REFERENCES**

1. Haylen B, Maher C, Barber M. International Continence Society (ICS) joint report on the terminology for female pelvic organ prolapse (POP). Int Urogynecol J. 2016;27(4):655-84.

2. Chow D, Rodríguez LV. Epidemiology and prevalence of pelvic organ prolapse. Current opinion in urology. 2013;23(4):293-8.

3. Haylen BT, De Ridder D, Freeman RM, Swift SE, Berghmans B, Lee J, et al. An International Urogynecological Association (IUGA)/International Continence Society (ICS) joint report on the terminology for female pelvic floor dysfunction. Neurourology and Urodynamics: Official Journal of the International Continence Society. 2010;29(1):4-20.

4. Weintraub AY, Glinter H, Marcus-Braun N. Narrative review of the epidemiology, diagnosis and pathophysiology of pelvic organ prolapse. International braz j urol. 2019;46:5-14.

5. Swift SE, Barber MD. Pelvic organ prolapse: defining the disease. LWW; 2010.

6. Bump RC, Mattiasson A, Bø K, Brubaker LP, DeLancey JO, Klarskov P, et al. The standardization of terminology of female pelvic organ prolapse and pelvic floor dysfunction. American journal of obstetrics and gynecology. 1996;175(1):10-7.

7. Dietz H. The aetiology of prolapse. International Urogynecology Journal. 2008;19(10):1323-9.

8. Schaffer JI, Wai CY, Boreham MK. Etiology of pelvic organ prolapse. Clinical obstetrics and gynecology. 2005;48(3):639-47.

9. Iglesia C, Smithling KR. Pelvic organ prolapse. American family physician. 2017;96(3):179-85.

10. Kuncharapu I, Majeroni BA, Johnson DW. Pelvic organ prolapse. American family physician. 2010;81(9):1111-7.

11. Weber A, Abrams P, Brubaker L, Cundiff G, Davis G, Dmochowski R, et al. The standardization of terminology for researchers in female pelvic floor disorders. International urogynecology journal. 2001;12(3):178-86.

12. Manonai J, Mouritsen L, Palma P, Contreras-Ortiz O, Korte JE, Swift S. The inter-system association between the simplified pelvic organ prolapse quantification system (S-POP) and the standard pelvic organ prolapse quantification system (POPQ) in describing pelvic organ prolapse. International urogynecology journal. 2011;22(3):347-52.

13. Swift S, Morris S, McKinnie V, Freeman R, Petri E, Scotti RJ, et al. Validation of a simplified technique for using the POPQ pelvic organ prolapse classification system. International Urogynecology Journal. 2006;17(6):615-20.

14. Obstetricians ACo, Gynecologists. Pelvic organ prolapse. Female Pelvic Medicine & Reconstructive Surgery. 2019;25(6):397-408.

15. Barber MD, Maher C. Epidemiology and outcome assessment of pelvic organ prolapse. International urogynecology journal. 2013;24(11):1783-90.

16. Hagen S, Glazener C, Sinclair L, Stark D, Bugge C. Psychometric properties of the pelvic organ prolapse symptom score. BJOG: an International Journal of Obstetrics & Gynaecology. 2009;116(1):25-31.

17. Vergeldt TF, Weemhoff M, IntHout J, Kluivers KB. Risk factors for pelvic organ prolapse and its recurrence: a systematic review. International urogynecology journal. 2015;26(11):1559-73.

18. Mothes A, Radosa M, Altendorf-Hofmann A, Runnebaum I. Risk index for pelvic organ prolapse based on established individual risk factors. Archives of gynecology and obstetrics. 2016;293(3):617-24.

19. Bradshaw KD, Corton MM, Halvorson LM, Hoffman BL, Schaffer M, Schorge JO. Williams gynecology: McGraw-Hill Education LLC.; 2016.

20. Shah AD, Kohli N, Rajan SS, Hoyte L. The age distribution, rates, and types of surgery for pelvic organ prolapse in the USA. International urogynecology journal. 2008;19(3):421-8.

21. Walker GJ, Gunasekera P. Pelvic organ prolapse and incontinence in developing countries: review of prevalence and risk factors. International urogynecology journal. 2011;22(2):127-35.

22. Lien YS, Chen GD, Ng SC. Prevalence of and risk factors for pelvic organ prolapse and lower urinary tract symptoms among women in rural Nepal. International Journal of Gynecology & Obstetrics. 2012;119(2):185-8.

23. Lawrence JM, Lukacz ES, Nager CW, Hsu J-WY, Luber KM. Prevalence and co-occurrence of pelvic floor disorders in community-dwelling women. Obstetrics & Gynecology. 2008;111(3):678-85.

24. De Boer T, Salvatore S, Cardozo L, Chapple C, Kelleher C, Van Kerrebroeck P, et al. Pelvic organ prolapse and overactive bladder. Neurourology and Urodynamics: Official Journal of the International Continence Society. 2010;29(1):30-9.

25. Siddique M, Ingraham C, Kudish B, Iglesia CB, Polland A. Hydronephrosis associated with pelvic organ prolapse: a systematic review. Female pelvic medicine & reconstructive surgery. 2020;26(3):212-8.

26. Jokhio AH, Rizvi RM, MacArthur C. Prevalence of pelvic organ prolapse in women, associated factors and impact on quality of life in rural Pakistan: population-based study. BMC women's health. 2020;20(1):1-7.

27. Gjerde JL, Rortveit G, Muleta M, Adefris M, Blystad A. Living with pelvic organ prolapse: voices of women from Amhara region, Ethiopia. International urogynecology journal. 2017;28(3):361-6.

28. Mirskaya M, Lindgren E-C, Carlsson M. Online reported women’s experiences of symptomatic pelvic organ prolapse after vaginal birth. BMC women's health. 2019;19(1):1-8.

29. Zeleke BM, Ayele TA, Woldetsadik MA, Bisetegn TA, Adane AA. Depression among women with obstetric fistula, and pelvic organ prolapse in northwest Ethiopia. BMC psychiatry. 2013;13(1):1-5.

30. Ghetti C, Lowder JL, Ellison R, Krohn M, Moalli P. Depressive symptoms in women seeking surgery for pelvic organ prolapse. International urogynecology journal. 2010;21(7):855-60.

31. Thompson JC, Rogers RG. Surgical management for pelvic organ prolapse and its impact on sexual function. Sexual medicine reviews. 2016;4(3):213-20.

32. Caruso S, Bandiera S, Cavallaro A, Cianci S, Vitale SG, Rugolo S. Quality of life and sexual changes after double transobturator tension-free approach to treat severe cystocele. European Journal of Obstetrics & Gynecology and Reproductive Biology. 2010;151(1):106-9.

33. Kenne K, Abreha M, Hart KD, Gregory WT, Nardos R. Surgical Management of Pelvic Organ Prolapse in Ethiopian Women: What Is the Preferred Approach? Female pelvic medicine & reconstructive surgery. 2020;26(2):e7-e12.

34. Dunivan GC, Anger JT, Alas A, Wieslander C, Sevilla C, Chu S, et al. Pelvic organ prolapse: a disease of silence and shame. Female pelvic medicine & reconstructive surgery. 2014;20(6):322.

35. Borsamo A, Oumer M, Asmare Y, Worku A. Factors associated with delay in seeking treatment among women with pelvic organ prolapse at selected general and referral hospitals of Southern Ethiopia, 2020. BMC Women's Health. 2021;21(1):1-8.

36. Adefris M, Abebe SM, Terefe K, Gelagay AA, Adigo A, Amare S, et al. Reasons for delay in decision making and reaching health facility among obstetric fistula and pelvic organ prolapse patients in Gondar University hospital, Northwest Ethiopia. BMC women's health. 2017;17(1):1-7.

37. Awwad J, Sayegh R, Yeretzian J, Deeb ME. Prevalence, risk factors, and predictors of pelvic organ prolapse: a community-based study. Menopause. 2012;19(11):1235-41.

38. Gedefaw G. Burden of pelvic organ prolapse in Ethiopia: a systematic review and meta-analysis. Investigative and clinical urology. 2020;20(1):166.

39. Belayneh T, Gebeyehu A, Adefris M, Rortveit G, Awoke T. Pelvic organ prolapse in Northwest Ethiopia: a population-based study. International urogynecology journal. 2019:1-9.

40. Muche HA, Kassie FY, Biweta MA, Gelaw KA, Debele TZ. Prevalence and associated factors of pelvic organ prolapse among women attending gynecologic clinic in referral hospitals of Amhara Regional State, Ethiopia. International Urogynecology Journal. 2021;32(6):1419-26.

41. Ballard K, Ayenachew F, Wright J, Atnafu H. Prevalence of obstetric fistula and symptomatic pelvic organ prolapse in rural Ethiopia. International urogynecology journal. 2016;27(7):1063-7.

42. Hagen S, Stark D, Glazener C, Dickson S, Barry S, Elders A, et al. Individualised pelvic floor muscle training in women with pelvic organ prolapse (POPPY): a multicentre randomised controlled trial. The Lancet. 2014;383(9919):796-806.

43. Wiegersma M, Panman CM, Hesselink LC, Malmberg AG, Berger MY, Kollen BJ, et al. Predictors of success for pelvic floor muscle training in pelvic organ prolapse. Physical therapy. 2019;99(1):109-17.

44. Wiegersma M, Panman CM, Kollen BJ, Berger MY, Lisman-Van Leeuwen Y, Dekker JH. Effect of pelvic floor muscle training compared with watchful waiting in older women with symptomatic mild pelvic organ prolapse: randomised controlled trial in primary care. Bmj. 2014;349.

45. Barber MD. Pelvic organ prolapse. Bmj. 2016;354.

46. Slieker-ten Hove M, Vierhout M, Bloembergen H, Schoenmaker G. Distribution of pelvic organ prolapse (POP) in the general population; Prevalence, severity, etiology and relation with the function of the pelvic floor muscles. 2004.

47. Aytan H, Ertunç D, Tok EC, Yaşa O, Nazik H. Prevalence of pelvic organ prolapse and related factors in a general female population. Turkish journal of obstetrics and gynecology. 2014;11(3):176.

48. Quiroz LH, Muñoz A, Shippey SH, Gutman RE, Handa VL. Vaginal parity and pelvic organ prolapse. The Journal of reproductive medicine. 2010;55(3-4):93.

49. Li Z, Xu T, Li Z, Gong J, Liu Q, Zhu L. An epidemiologic study of pelvic organ prolapse in rural Chinese women: a population-based sample in China. International urogynecology journal. 2019;30(11):1925-32.

50. Li Z, Xu T, Li Z, Gong J, Liu Q, Wang Y, et al. An epidemiologic study on symptomatic pelvic organ prolapse in obese Chinese women: a population-based study in China. Diabetes, metabolic syndrome and obesity: targets and therapy. 2018;11:761.

51. Scherf C, Morison L, Fiander A, Ekpo G, Walraven G. Epidemiology of pelvic organ prolapse in rural Gambia, West Africa. BJOG: an international journal of obstetrics and gynaecology. 2002;109(4):431-6.

52. Akmel M, Segni H. Pelvic organ prolapse in Jimma University specialized hospital, Southwest Ethiopia. Ethiopian journal of health sciences. 2012;22(2).

53. Kim S, Harvey M-A, Johnston S. A review of the epidemiology and pathophysiology of pelvic floor dysfunction: do racial differences matter? Journal of Obstetrics and Gynaecology Canada. 2005;27(3):251-9.

54. Durnea C, Khashan A, Kenny L, Durnea U, Smyth M, O’Reilly B. Prevalence, etiology and risk factors of pelvic organ prolapse in premenopausal primiparous women. International urogynecology journal. 2014;25(11):1463-70.

55. Horst W, Do Valle JB, Silva JC, Gascho CLL. Pelvic organ prolapse: prevalence and risk factors in a Brazilian population. International urogynecology journal. 2017;28(8):1165-70.

56. Asresie A, Admassu E, Setegn T. Determinants of pelvic organ prolapse among gynecologic patients in Bahir Dar, North West Ethiopia: a case–control study. International journal of women's health. 2016;8:713.

57. Nygaard I, Bradley C, Brandt D. Pelvic organ prolapse in older women: prevalence and risk factors. Obstetrics & Gynecology. 2004;104(3):489-97.

58. Forner LB, Beckman EM, Smith MD. Symptoms of pelvic organ prolapse in women who lift heavy weights for exercise: a cross-sectional survey. International urogynecology journal. 2020;31(8):1551-8.

59. Middlekauff ML, Egger MJ, Nygaard IE, Shaw JM. The impact of acute and chronic strenuous exercise on pelvic floor muscle strength and support in nulliparous healthy women. American journal of obstetrics and gynecology. 2016;215(3):316. e1-. e7.

60. Ali‐Ross N, Smith A, Hosker G. The effect of physical activity on pelvic organ prolapse. BJOG: An International Journal of Obstetrics & Gynaecology. 2009;116(6):824-8.

61. Masenga GG, Shayo BC, Rasch V. Prevalence and risk factors for pelvic organ prolapse in Kilimanjaro, Tanzania: a population based study in Tanzanian rural community. PloS one. 2018;13(4):e0195910.

62. Cheung RY, Chan SS, Shek KL, Chung TK, Dietz HP. Pelvic organ prolapse in Caucasian and East Asian women: a comparative study. Ultrasound in Obstetrics & Gynecology. 2019;53(4):541-5.

63. Hendrix SL, Clark A, Nygaard I, Aragaki A, Barnabei V, McTiernan A. Pelvic organ prolapse in the Women's Health Initiative: gravity and gravidity. American journal of obstetrics and gynecology. 2002;186(6):1160-6.

64. Ford AT, Eto CU, Smith M, Northington GM. Racial differences in pelvic organ prolapse symptoms among women undergoing pelvic reconstructive surgery for prolapse. Female pelvic medicine & reconstructive surgery. 2019;25(2):130-3.

65. Abdool Z, Dietz H, Lindeque B. Ethnic differences in the levator hiatus and pelvic organ descent: a prospective observational study. Ultrasound in Obstetrics & Gynecology. 2017;50(2):242-6.

66. Shek K, Krause HG, Wong V, Goh J, Dietz HP. Is pelvic organ support different between young nulliparous African and Caucasian women? Ultrasound in Obstetrics & Gynecology. 2016;47(6):774-8.

67. Blomquist JL, Muñoz A, Carroll M, Handa VL. Association of delivery mode with pelvic floor disorders after childbirth. Jama. 2018;320(23):2438-47.

68. Leng B, Zhou Y, Du S, Liu F, Zhao L, Sun G, et al. Association between delivery mode and pelvic organ prolapse: a meta-analysis of observational studies. European Journal of Obstetrics & Gynecology and Reproductive Biology. 2019;235:19-25.

69. Leijonhufvud Å, Lundholm C, Cnattingius S, Granath F, Andolf E, Altman D. Risks of stress urinary incontinence and pelvic organ prolapse surgery in relation to mode of childbirth. American journal of obstetrics and gynecology. 2011;204(1):70. e1-. e7.

70. Cattani L, Decoene J, Page A-S, Weeg N, Deprest J, Dietz HP. Pregnancy, labour and delivery as risk factors for pelvic organ prolapse: a systematic review. International Urogynecology Journal. 2021:1-9.

71. Tegerstedt G, Maehle-Schmidt M, Nyrén O, Hammarström M. Prevalence of symptomatic pelvic organ prolapse in a Swedish population. International Urogynecology Journal. 2005;16(6):497-503.

72. Dietz HP, Rozsa D, Subramaniam N, Friedman T. Does Vaginal Parity Alter the Association Between Symptoms and Signs of Pelvic Organ Prolapse? Journal of Ultrasound in Medicine. 2021;40(4):675-9.

73. Eleje G, Udegbunam O, Ofojebe C, Adichie C. Determinants and management outcomes of pelvic organ prolapse in a low resource setting. Annals of medical and health sciences research. 2014;4(5):796-801.

74. Zenebe CB, Chanie WF, Aregawi AB, Andargie TM, Mihret MS. The effect of women’s body mass index on pelvic organ prolapse: a systematic review and meta analysis. BMC Reproductive Health. 2021;18(1):1-9.

75. Jackson S, Eckford S, Abrams P, Avery N, Tarlton J, Bailey A. Changes in metabolism of collagen in genitourinary prolapse. The Lancet. 1996;347(9016):1658-61.

76. Beketie ED, Tafese WT, Assefa ZM, Berriea FW, Tilahun GA, Shiferaw BZ, et al. Symptomatic pelvic floor disorders and its associated factors in South-Central Ethiopia. Plos one. 2021;16(7):e0254050.

77. Lim VF, Khoo JK, Wong V, Moore KH. Recent studies of genetic dysfunction in pelvic organ prolapse: the role of collagen defects. Australian and New Zealand Journal of Obstetrics and Gynaecology. 2014;54(3):198-205.

78. Giri A, Hartmann KE, Hellwege JN, Edwards DRV, Edwards TL. Obesity and pelvic organ prolapse: a systematic review and meta-analysis of observational studies. American journal of obstetrics and gynecology. 2017;217(1):11-26. e3.

79. Henok A. Prevalence and factors associated with pelvic organ prolapse among pedestrian back-loading women in Bench Maji Zone. Ethiopian journal of health sciences. 2017;27(3):263-72.

80. Shalom DF, Lin SN, St Louis S, Winkler HA. Effect of age, body mass index, and parity on Pelvic Organ Prolapse Quantification system measurements in women with symptomatic pelvic organ prolapse. Journal of Obstetrics and Gynaecology research. 2012;38(2):415-9.

81. Allen-Brady K, Norton PA, Hill AJ, Rowe K, Cannon-Albright LA. Risk of pelvic organ prolapse treatment based on extended family history. American journal of obstetrics and gynecology. 2020;223(1):105. e1-. e8.

82. Samimi P, Jones SH, Giri A. Family history and pelvic organ prolapse: a systematic review and meta-analysis. International Urogynecology Journal. 2020:1-16.

83. Levin PJ, Visco AG, Shah SH, Fulton RG, Wu JM. Characterizing the phenotype of advanced pelvic organ prolapse. Female pelvic medicine & reconstructive surgery. 2012;18(5):299.

84. McLennan MT, Harris JK, Kariuki B, Meyer S. Family history as a risk factor for pelvic organ prolapse. International Urogynecology Journal. 2008;19(8):1063-9.

85. Isık H, Aynıoglu O, Sahbaz A, Selimoglu R, Timur H, Harma M. Are hypertension and diabetes mellitus risk factors for pelvic organ prolapse? European Journal of Obstetrics & Gynecology and Reproductive Biology. 2016;197:59-62.

86. Dunivan GC, Cichowski SB, Komesu YM, Fairchild PS, Anger JT, Rogers RG. Ethnicity and variations of pelvic organ prolapse bother. International urogynecology journal. 2014;25(1):53-9.

87. Maxwell M, Berry K, Wane S, Hagen S, McClurg D, Duncan E, et al. Pelvic floor muscle training for women with pelvic organ prolapse: the PROPEL realist evaluation. Health Services and Delivery Research. 2020;8(47).

88. Vitale SG, Caruso S, Rapisarda AMC, Valenti G, Rossetti D, Cianci S, et al. Biocompatible porcine dermis graft to treat severe cystocele: impact on quality of life and sexuality. Archives of gynecology and obstetrics. 2016;293(1):125-31.

89. Hefni M, Barry JA, Koukoura O, Meredith J, Mossa M, Edmonds S. Long-term quality of life and patient satisfaction following anterior vaginal mesh repair for cystocele. Archives of gynecology and obstetrics. 2013;287(3):441-6.

90. Digesu GA, Khullar V, Cardozo L, Robinson D, Salvatore S. P-QOL: a validated questionnaire to assess the symptoms and quality of life of women with urogenital prolapse. International Urogynecology Journal. 2005;16(3):176-81.

91. Panman CM, Wiegersma M, Kollen BJ, Berger MY, Lisman‐Van Leeuwen Y, Vermeulen K, et al. Two‐year effects and cost‐effectiveness of pelvic floor muscle training in mild pelvic organ prolapse: a randomised controlled trial in primary care. BJOG: An International Journal of Obstetrics & Gynaecology. 2017;124(3):511-20.

92. Fritel X, Varnoux N, Zins M, Breart G, Ringa V. Symptomatic pelvic organ prolapse at midlife, quality of life, and risk factors. Obstetrics and gynecology. 2009;113(3):609.

93. Barber MD, Brubaker L, Nygaard I, Wheeler TL. Defining success after surgery for pelvic organ prolapse. Obstetrics and gynecology. 2009;114(3):600.

94. Hagen S, Stark D. Conservative prevention and management of pelvic organ prolapse in women. Cochrane Database of Systematic Reviews. 2011(12).

95. Culligan PJ. Nonsurgical management of pelvic organ prolapse. Obstetrics & Gynecology. 2012;119(4):852-60.

96. Laganà AS, La Rosa VL, Rapisarda AMC, Vitale SG. Pelvic organ prolapse: the impact on quality of life and psychological well-being. Journal of Psychosomatic Obstetrics & Gynecology. 2018;39(2):164-6.

97. Rantell A. Vaginal pessaries for pelvic organ prolapse and their impact on sexual function. Sexual medicine reviews. 2019;7(4):597-603.

98. Li C, Gong Y, Wang B. The efficacy of pelvic floor muscle training for pelvic organ prolapse: a systematic review and meta-analysis. International urogynecology journal. 2016;27(7):981-92.

99. Brækken IH, Majida M, Engh ME, Bø K. Can pelvic floor muscle training improve sexual function in women with pelvic organ prolapse? A randomized controlled trial. The journal of sexual medicine. 2015;12(2):470-80.

100. Brækken IH, Majida M, Engh ME, Bø K. Can pelvic floor muscle training reverse pelvic organ prolapse and reduce prolapse symptoms? An assessor-blinded, randomized, controlled trial. American journal of obstetrics and gynecology. 2010;203(2):170. e1-. e7.

101. Bø K, Nygaard IE. Is physical activity good or bad for the female pelvic floor? A narrative review. Sports Medicine. 2020;50(3):471-84.

102. Nygaard IE, Shaw JM. Physical activity and the pelvic floor. American journal of obstetrics and gynecology. 2016;214(2):164-71.

103. Ge J, Wei X, Zhang H, Fang G. Pelvic floor muscle training in the treatment of pelvic organ prolapse: A meta-analysis of randomized controlled trials. Actas Urológicas Españolas (English Edition). 2021;45(1):73-82.

104. Stüpp L, Resende APM, Oliveira E, Castro RA, Girão MJBC, Sartori MGF. Pelvic floor muscle training for treatment of pelvic organ prolapse: an assessor-blinded randomized controlled trial. International urogynecology journal. 2011;22(10):1233-9.

105. Ouchi M, Kato K, Gotoh M, Suzuki S. Physical activity and pelvic floor muscle training in patients with pelvic organ prolapse: a pilot study. International urogynecology journal. 2017;28(12):1807-15.

106. Doaee M, Moradi-Lakeh M, Nourmohammadi A, Razavi-Ratki SK, Nojomi M. Management of pelvic organ prolapse and quality of life: a systematic review and meta-analysis. International urogynecology journal. 2014;25(2):153-63.

107. Due U, Brostrøm S, Lose G. The 12‐month effects of structured lifestyle advice and pelvic floor muscle training for pelvic organ prolapse. Acta Obstetricia et Gynecologica Scandinavica. 2016;95(7):811-9.

108. Due U, Brostrøm S, Lose G. Lifestyle advice with or without pelvic floor muscle training for pelvic organ prolapse: a randomized controlled trial. International urogynecology journal. 2016;27(4):555-63.

109. Giarenis I, Robinson D. Prevention and management of pelvic organ prolapse. F1000prime reports. 2014;6.

110. Patel DA, Xu X, Thomason AD, Ransom SB, Ivy JS, DeLancey JO. Childbirth and pelvic floor dysfunction: an epidemiologic approach to the assessment of prevention opportunities at delivery. American journal of obstetrics and gynecology. 2006;195(1):23-8.

111. Wilkins MF, Wu JM. Epidemiology of pelvic organ prolapse. Current Obstetrics and Gynecology Reports. 2016;5(2):119-23.

112. Mattsson NK, Karjalainen PK, Tolppanen A-M, Heikkinen A-M, Sintonen H, Härkki P, et al. Pelvic organ prolapse surgery and quality of life—a nationwide cohort study. American journal of obstetrics and gynecology. 2020;222(6):588. e1-. e10.

113. Ma Y, Xu T, Zhang Y, Kang J, Ma C, Zhu L. Validation of the Chinese version of the Pelvic Organ Prolapse Symptom Score (POP-SS). Menopause. 2020;27(9):1053-9.

114. Belayneh T, Gebeyehu A, Adefris M, Rortveit G, Genet T. Validation of the Amharic version of the pelvic organ prolapse symptom score (POP-SS). International urogynecology journal. 2019;30(1):149-56.

115. Özengin N, Kaya S, Orhan C, Bakar Y, Duran B, Ankaralı H, et al. Turkish adaptation of the Pelvic Organ Prolapse Symptom Score and its validity and reliability. International urogynecology journal. 2017;28(8):1217-22.

116. Gjersing L, Caplehorn JR, Clausen T. Cross-cultural adaptation of research instruments: language, setting, time and statistical considerations. BMC medical research methodology. 2010;10(1):1-10.

117. Beaton DE, Bombardier C, Guillemin F, Ferraz MB. Guidelines for the process of cross-cultural adaptation of self-report measures. Spine. 2000;25(24):3186-91.

118. Bland JM, Altman DG. Validating scales and indexes. Bmj. 2002;324(7337):606-7.

119. Polit DF, Beck CT. The content validity index: are you sure you know what's being reported? Critique and recommendations. Research in nursing & health. 2006;29(5):489-97.

120. Terwee CB, Bot SD, de Boer MR, van der Windt DA, Knol DL, Dekker J, et al. Quality criteria were proposed for measurement properties of health status questionnaires. Journal of clinical epidemiology. 2007;60(1):34-42.

121. Kline P. The handbook of psychological testing: Psychology Press; 2000.

122. Rortveit G, Brown JS, Thom DH, Van Den Eeden SK, Creasman JM, Subak LL. Symptomatic pelvic organ prolapse: prevalence and risk factors in a population-based, racially diverse cohort. Obstetrics & Gynecology. 2007;109(6):1396-403.

123. Fiona R. Assessment of Pelvic Organ Prolapse: a practical guide to the pelvic Organ Prolapse Quantification Obstetrics, Gynecology and Reproductive Health. 2014;24(6).

124. Burns KE, Duffett M, Kho ME, Meade MO, Adhikari NK, Sinuff T, et al. A guide for the design and conduct of self-administered surveys of clinicians. Cmaj. 2008;179(3):245-52.

125. Davis LL. Instrument review: Getting the most from a panel of experts. Applied nursing research. 1992;5(4):194-7.

126. Lynn MR. Determination and quantification of content validity. Nursing research. 1986.

127. DeVon HA, Block ME, Moyle‐Wright P, Ernst DM, Hayden SJ, Lazzara DJ, et al. A psychometric toolbox for testing validity and reliability. Journal of Nursing scholarship. 2007;39(2):155-64.

128. Kaiser HF. The application of electronic computers to factor analysis. Educational and psychological measurement. 1960;20(1):141-51.

129. Bartlett MS. Tests of significance in factor analysis. British Journal of statistical psychology. 1950;3(2):77-85.

130. Ledesma RD, Valero-Mora P, Macbeth G. The scree test and the number of factors: a dynamic graphics approach. The Spanish journal of psychology. 2015;18.

131. Furr RM. Psychometrics: an introduction: SAGE publications; 2021.

132. Streiner DL, Norman GR, Cairney J. Health measurement scales: a practical guide to their development and use: Oxford University Press, USA; 2015.

133. Clark LA, Watson D. Constructing validity: Basic issues in objective scale development. 2016.

134. Sidama Region Health Bureau S. Sidama Region Health Bureau, Health facility data with Catchment Population (Updated Meskerem 2014 E.C) Sidama Region Health Bureau, 2021.

135. Central Statistics Agency C. Population Projection of Ethiopia for All Regions At Wereda Level from 2014 – 2017. In: Agency CS, editor. Addis Ababa2013.

136. Areru HA, Dangisso MH, Lindtjørn B. Births and deaths in Sidama in southern Ethiopia: findings from the 2018 Dale-Wonsho Health and Demographic Surveillance System (HDSS). Global health action. 2020;13(1):1833511.

137. Olsen C, St George D. Cross-sectional study design and data analysis. College entrance examination board. 2004;26(03):2006.

138. Skinner C, Wakefield J. Introduction to the design and analysis of complex survey data. Statistical Science. 2017;32(2):165-75.

139. Potthoff RF, Woodbury MA, Manton KG. “Equivalent sample size” and “equivalent degrees of freedom” refinements for inference using survey weights under superpopulation models. Journal of the American Statistical Association. 1992;87(418):383-96.

140. Hahs‐Vaughn DL. Analysis of data from complex samples. International Journal of Research & Method in Education. 2006;29(2):165-83.

141. Muthén LK, Muthén BO. 1998–2010 Mplus user‘s guide. Muthén and Muthén. 2010:39-49.

142. ICF CSACEa. Ethiopia Demographic and Health Survey 2016. Addis Ababa, Ethiopia, and Rockville, Maryland, USA: 2016.

143. Gotoh M. Scored ICIQ-SF (International Consultation on Incontinence Questionnaire-Short Form) for symptoms and QOL assessment in patients with urinary incontinence. JNBS 2001. 2001;12:227-31.

144. Barber MD, Kuchibhatla MN, Pieper CF, Bump RC. Psychometric evaluation of 2 comprehensive condition-specific quality of life instruments for women with pelvic floor disorders. American journal of obstetrics and gynecology. 2001;185(6):1388-95.

145. Belayneh T, Gebeyehu A, Adefris M, Rortveit G, Genet T. Translation, transcultural adaptation, reliability and validation of the pelvic organ prolapse quality of life (P-QoL) in Amharic. Health and quality of life outcomes. 2019;17(1):1-11.

146. Organization WH. Physical status: The use of and interpretation of anthropometry, Report of a WHO Expert Committee: World Health Organization; 1995.

147. Lepkowski JM. Statistical methodologies for analyzing a complex sample survey: US Department of Health and Human Services, Public Health Service, Centers …; 1988.

148. Heeringa SG, West BT, Berglund PA. Applied survey data analysis: chapman and hall/CRC; 2010.

149. Pannucci CJ, Wilkins EG. Identifying and avoiding bias in research. Plastic and reconstructive surgery. 2010;126(2):619.

150. Pourhoseingholi MA, Baghestani AR, Vahedi M. How to control confounding effects by statistical analysis. Gastroenterology and hepatology from bed to bench. 2012;5(2):79.

151. Daoud JI, editor Multicollinearity and regression analysis. Journal of Physics: Conference Series; 2017: IOP Publishing.

152. Hosmer DW, Lemeshow S, Sturdivant RX. Applied logistic regression: Wiley New York; 2000.

153. Archer KJ, Lemeshow S, Hosmer DW. Goodness-of-fit tests for logistic regression models when data are collected using a complex sampling design. Computational Statistics & Data Analysis. 2007;51(9):4450-64.

154. Jaadi Z. A step-by-step explanation of principal component analysis (PCA). Retrieved June. 2021;7:2021.

155. Castaño A, Fernández-Navarro F, Hervás-Martínez C. PCA-ELM: a robust and pruned extreme learning machine approach based on principal component analysis. Neural processing letters. 2013;37(3):377-92.

156. Hubert M, Rousseeuw PJ, Vanden Branden K. ROBPCA: a new approach to robust principal component analysis. Technometrics. 2005;47(1):64-79.

157. Hemming K, Eldridge S, Forbes G, Weijer C, Taljaard M. How to design efficient cluster randomised trials. bmj. 2017;358.

158. Donner A, Birkett N, Buck C. Randomization by cluster: sample size requirements and analysis. American journal of epidemiology. 1981;114(6):906-14.

159. Killip S, Mahfoud Z, Pearce K. What is an intracluster correlation coefficient? Crucial concepts for primary care researchers. The Annals of Family Medicine. 2004;2(3):204-8.

160. Hayes RJ, Moulton LH. Cluster randomised trials: Chapman and Hall/CRC; 2017.

161. Rutterford C, Copas A, Eldridge S. Methods for sample size determination in cluster randomized trials. International journal of epidemiology. 2015;44(3):1051-67.

162. Bø K. Pelvic floor muscle exercise for the treatment of stress urinary incontinence: an exercise physiology perspective. International Urogynecology Journal. 1995;6(5):282-91.

163. Brækken IH, Majida M, Engh ME, Bø K. Morphological changes after pelvic floor muscle training measured by 3-dimensional ultrasonography: a randomized controlled trial. Obstetrics & Gynecology. 2010;115(2):317-24.

164. Yalcin I, Bump RC. Validation of two global impression questionnaires for incontinence. American journal of obstetrics and gynecology. 2003;189(1):98-101.

165. Bland JM, Altman DG. Multiple significance tests: the Bonferroni method. Bmj. 1995;310(6973):170.

166. Sedgwick P. Multiple significance tests: the Bonferroni correction. Bmj. 2012;344.

167. Hsu J. Multiple comparisons: theory and methods: CRC Press; 1996.

168. Tabachnick BG, Fidell LS, Ullman JB. Using multivariate statistics: Pearson Boston, MA; 2007.

169. Barber M, Walters M, Bump R. Short forms of two condition-specific quality-of-life questionnaires for women with pelvic floor disorders (PFDI-20 and PFIQ-7). American journal of obstetrics and gynecology. 2005;193(1):103-13.

170. Srikrishna S, Robinson D, Cardozo L. Validation of the Patient Global Impression of Improvement (PGI-I) for urogenital prolapse. International urogynecology journal. 2010;21(5):523-8.

# **ANNEX I: Information Sheet**

**Hawassa University**

**College of Medicine and Health Science**

**School of Public Health**

**Title of the research:** Epidemiology of Pelvic Organ Prolapse in Sidama National Regional State, Ethiopia, 2022.

**Principal investigator: Melese Siyoum**

**Greetings!** My name is _________. I am here on behalf of Mr. **Melese Siyoum** a PhD student at Hawassa University. He is conducting a research for the partial fulfilment of PhD degree in Public Health on the title mentioned above. You are chosen to participate in this study because you are resident of this kebele. The choice is made randomly. Before you decide whether or not to participate, I would like to explain about the study and what is expected from you.

**The Objective of the study:** The study will assess prevalence of and risk factors for Pelvic Organ Prolapse in Sidama National Regional State, Ethiopia. In addition, volunteer women who have a symptom of pelvic organ prolapse will be threated either conservatively or referred to Hospital for further management.

**Procedure:** The data collection procedure involves interviewer-administered questionnaire and Pelvic examination to quantify the degree of pelvic organ prolapse. Now, I will ask you a set of questions using a structured questionnaire. After signing the consent form, I will then ask you the relevant questions and your responses will be written on the questionnaire. The interview will take about 40-50 minutes. The content of the questionnaire includes sociodemographic characteristics, wealth index, obstetric history, issues related to symptoms of the prolapse, and how it affected your quality of life. Finally, you will be given an appointment and will be examined by trained health care providers at a nearby health facility. A trained health care provider will confirm whether you have a prolapse or not. If you have a prolapse, you will be included in the trial study of two different treatment categories. In the trial study, there will be a five time visit at weeks 0, 2, 6, 10 and 15. The transportation cost will be covered by the research team.

**The Benefit of the study:** This study has direct and indirect benefits for women and the health care system. All women who participate in this study will be screened for the prolapse free of charge (without payment). In addition, the transportation cost and time compensation will be covered by the research team. Women who have mild to moderate prolapse (stage I-III) will be managed according to the trial protocol. Advanced cases will be referred to Yirgalem Hamline fistula center for treatment if you are volunteer. Beyond direct individual benefit, the finding of this study will identify the burden of the disease and help the regional health bureau to plan appropriate intervention. The result of the trial will be used by the concerned health offices and organization concerned to improve women health.

**The Risk of the study:** The intervention (procedure) going to be provided has no identified risks except the dedication of certain minutes for responding and repeated appointment to health facility (five times visit). In addition, you may face some discomfort from pelvic examination and some distress while exposing your private organ (genitalia). The interview and pelvic examination will be conducted at a private room and all information will be kept confidential.

**The Rights of participants:** You have a right to refuse participation totally or partially and even you can withdraw at any time. There is no risk you will face if you decline to participate.

**Confidentiality:** All information you give will be strictly confidential and will be kept safe and secure. Your role in the success of the research is important and I appreciate your contribution to the research.

Do you agree to participate in the research?

1- No (say thank you) 2- Yes (take informed consent)

**Informed consent**

The objective, benefits, harms, procedures and confidentiality of the study has been explained to me in the language I comprehend. I further understand that, taking part in this study and withdraw from participating at any time without having reason is purely voluntary. I agree to participate in this study.

Participant:

Sign.............................. Date........................

Data collector:

Sign................................. Date..................................

# **ANNEX II: English Version Questionnaire**

**Hawassa University**

**College of Medicine and Health Sciences**

**School of Public Health**

**Part One: Respondent’s socio-demographic characteristics**

**Directions**

Ask the following questions one at a time and write the response on the space provided or encircle the appropriate response from the options listed. Allow the participant to take time to remember the correct answer when needed.

**Woreda_________ kebele________ Enumeration Area____________ Code_____ Mobile______________________**

| **Sr. No.** | **Questions** | **Response category** | **Skip to** |
| --- | --- | --- | --- |
|  | What is your current Age? | __________years completed |  |
|  | Where is your permanent place of residence? | 1. Urban 2. Rural |  |
|  | What is your current marital status? | 1. Never married 2. Married 3. Widowed 4. Divorced 5. Separated |  |
|  | What is your Religion? | 1. Ethiopian Orthodox 2. Protestant 3. Muslim 4. Catholic 5. Adventist 6. Other(specify)____________ |  |
|  | What is your main Occupation? | 1. Housewife 2. Farmer 3. Government employee 4. Nongovernmental employee 5. Private Business 6. Student 7. Others Specify)____________ |  |
|  | What is your Ethnicity? | 1. Sidama 2. Wolayta 3. Amhara 4. Gurage 5. Oromo 6. Other(specify)___________ |  |
|  | What is your level of education? | 1. Unable to read and write 2. Able to read and write without formal education 3. Primary Education 4. Secondary Education 5. Above secondary |  |
|  | What is your Husbands’ level of education?  Ask only if she is married (refer back to Q-103). | 1. Unable to read and write 2. Able to read and write without formal education 3. Primary Education 4. Secondary Education 5. Above secondary |  |
| **Part-II: Lifestyle Questions**  Ask the following questions one at a time and write the response on the space provided or encircle the appropriate response from the options listed. | | | |
|  | On average, for how long do you carry heavy objects/day (water, big baby, wood, stone, cereal, or grain) | -------- hrs |  |
|  | Have you ever smoked cigarettes? | 1. No 2. Yes |  |
|  | Do you have a family member who had Pelvic organ prolapse? | 1. No 2. Yes |  |
|  | Did you ever have a chronic cough (>3weeks) | 1. No 2. Yes |  |
|  | Did you ever have constipation (difficulty of defecation? | 1. No 2. Yes |  |
|  | Did you ever have a confirmed diabetes mellitus? | 1. No 2. Yes |  |

**Part III: Respondents’ Obstetrics and Gynecologic related factors**

| **Sr. No.** | **Questions** | **Choices of response** | **Skip to** |
| --- | --- | --- | --- |
|  | What was your age when you married for the first time? | --------------- years completed |  |
|  | Did you have a history of pregnancy terminated before seven months? | 1. No 2. Yes |  |
|  | Did you give childbirth after seven months of pregnancy? | 1. No 2. Yes | If no, go to Q312 |
|  | If yes, what was your age at first delivery? | --------------- in years  999……… don’t know |  |
|  | How many children you delivered? | ___________no. of delivery |  |
|  | What was your age at last delivery? | ---------- years |  |
|  | Which mode of delivery have you ever faced?  More than one option is possible | 1. Spontaneous vaginal delivery 2. Cesarean delivery 3. Assisted instrumental delivery |  |
|  | Have you ever experienced labor that lasted more than one day and night (>24hrs)? | 1. No 2. Yes |  |
|  | Have you ever given birth at home (previously)? | 1. No 2. Yes |  |
|  | Where did you deliver your last child? | 1. At home 2. Health facility |  |
|  | When did you start household work after delivery of your last baby? | ----------- days  ……….. weeks after delivery |  |
|  | Do you have a history of pelvic surgery (episiotomy, hysterectomy, trauma, tumor removal) | 1. No 2. Yes |  |
|  | Is your menstrual cycle stopped permanently (more than 3 cycles)? Without contraceptive use? | 1. No 2. Yes |  |
|  | If yes for Q-313, for how long? | ------------- |  |

| **Part-IV: Study participants wealth Index**  Ask (and observe when possible) the following questions one at a time and write the response on the space provided or encircle the appropriate response from the options listed. | | | | | |
| --- | --- | --- | --- | --- | --- |
|  | What is the main source of drinking water for members of your household? | Piped water:   - 1. Piped into dwelling   2. Piped to yard/plot   3. Public tab/standpipe   4. Borehole | | |  |
|  |  | Dug well:   1. Protected well 2. Unprotected well | | |  |
|  |  | Water from spring:   1. Protected spring 2. Unprotected spring | | |  |
|  |  | Surface water:   - - - 1. River/lake/pond/stream/dam | | |  |
|  | What is the main source of water used by your household for other purposes such as cooking and handwashing? | Piped water:   1. Piped into dwelling 2. Piped to yard/plot 3. Public tab/standpipe 4. Borehole | | |  |
|  |  | Dug well:   1. Protected well 2. Unprotected well | | |  |
|  |  | Water from spring:   1. Protected spring 2. Unprotected spring | | |  |
|  |  | Surface water:   1. River/lake/pond/stream/dam | | |  |
|  | Where is that water source located? | 1. In own dwelling  2. In own yard/plot  3. Elsewhere | | |  |
|  | How long does it take to go there, get water, and come back? | 1. Minutes __________ 2. Don’t know | | |  |
|  | In the past two weeks, was the water from this source not available for at least one full day? | 1. N0 2. Yes 3. Don’t know | | |  |
|  | Do you do anything to the water to make it safer to drink? | 1. No ------------------------------------- 2. Yes | | | 4.8 |
|  | What do you usually do to make the water safer to Drink?  Record all mentioned | 1. Boil 2. Add bleach/chlorine 3. Strain through a cloth 4. Use water filter (Sand/composite/etc.) 5. Solar disinfection 6. Let it stand and settle | | |  |
|  | What kind of toilet facility do members of your household usually use? | **FLUSH OR POUR-FLUSH TOILET**   1. Flush to a piped sewer system 2. Flush to septic tank 3. flush to a pit latrine 4. Flush to somewhere else 5. Flush, don't know where   **PIT LATRINE**   1. Ventilated improved pit latrine 2. Pit latrine with slab 3. Pit latrine without slab/open pit 4. composting toilet 5. bucket toilet 6. Hanging toilet/hanging latrine 7. No facility/bush/field | | |  |
|  | Do you share this toilet facility with other households? | 1. No 2. Yes | | |  |
|  | Including your own household, how many households use this toilet facility? | 1. Less than 10 2. 10 or more 3. Don't know | | |  |
|  | Where is this toilet facility located? | 1. In own dwelling 2. In own yard/plot 3. Elsewhere | | |  |
|  | What type of fuel does your household mainly use for cooking? | 1. Electricity 2. Liquid petroleum gas 3. Natural gas 4. Biogas 5. Kerosene 6. Charcoal 7. Wood 8. Straw/grass 9. Agricultural crop 10. Animal dung 11. No food cooked in house | | |  |
|  | Is the cooking usually done in the house, in a separate building, or outdoors? | 1. In the house 2. In separate building 3. Outdoors 4. Other | | |  |
|  | Do you have a separate room which is used as a kitchen? | 1. Yes 2. No | | |  |
|  | Who is the owner of the house? | 1. Me 2. Rental 3. Family 4. Relative 5. Others (specify)_________________ | | |  |
|  | How many rooms in this household are used for sleeping? | --------Rooms | | |  |
|  | Main material of the roof of the house? | 1. Natural roofing (no roof, mud, and sod) 2. Rudimentary roofing (rustic mat/plastic sheet, reed/bamboo, wood planks, and cardboard) 3. Finished roofing (metal/corrugated iron, wood, calamine/cement, ceramic tiles, roofing shingles) | | |  |
|  | Main material of the floor of the house? | 1. Natural floor (Earth/sand, dung) 2. Rudimentary floor (wood planks, and palm/bamboo) 3. Finished floor (parquet or polished wood, vinyl or asphalt strips/ plastic tiles, cement, ceramic tiles, carpet) | | |  |
|  | Does this household own any livestock, herds, other farm animals, or poultry? | 1. No 2. Yes | | |  |
| - - 1. 4 | How many of the following animals does this household own?  IF NONE, RECORD '00'.  IF 95 OR MORE, RECORD '95'.  IF UNKNOWN, RECORD '98'. | 1. Cows bulls_______________ 2. Other cattle______________ 3. Horses/Donkeys/Mules ____ 4. Camels________ 5. Goats _________ 6. Sheep _________ 7. Chickens/poultry ______ 8. Beehives ___________ | | |  |
| - - 1. 421 | Do you have separate rooms for cattle? | 1. Yes 2. No | | |  |
| - - 1. 422 | Does any member of this household own any agricultural land? | 1. No --------------------------------------- 2. Yes | | | 4.24 |
| - - 1. 423 | How many hectares of agricultural land do members of this household own? | ___________hectares | | |  |
| - - 1. 424 | Which of the following does your household have? (Multiple answers possible) |  | Yes (1) | No (0) |  |
|  |  | 1. Electricity---------- 2. Radio--------------- 3. Television --------- 4. Non-mobile telephone---------- 5. Computer ---------- 6. Refrigerator ------- 7. Table---------------- 8. Chair --------------- 9. Bed with spring matters------------ 10. Electric mitad ----- 11. Kerosene lamp/pressure------ 12. Lamp--------------- |  |  |  |
|  | Does any member of this household own: |  | Yes (1) | No(0) |  |
|  |  | 1. Watch -------------- 2. Mobile phone------ 3. Bicycle------------- 4. Motorcycle/scooter- 5. Animal-drawn cart- 6. Car/truck ------------ 7. Boat with motor----- 8. Baggage ------------ |  |  |  |
| - - 1. 426 | Does any member of this household have a bank account? | 1. No 2. Yes | | |  |
| - - 1. 427 | Does any member of this household have a micro finance account? | 1. No 2. Yes | | |  |
|  | How often does anyone smoke inside your house?  Would you say daily, weekly, monthly, less often than once a month, or never? | 1. Daily 2. Weekly 3. Monthly 4. Less often than once a month 5. Never | | |  |

**PART-V: POP-SS**

**Direction**

The following seven questionnaires are standard tools used to measure prolapse symptoms. Each item/question has 0 to 4 possible response scales. Inform the participant how to give a rate for each item based on how often she felt the prolapse symptom in the last four weeks (Put ‘X’ in the right box under each response scale).

Dear participant, please tell me how often did you feel the following POP symptoms in the last four weeks? (Please give the rating scale as follows: 0= never felt symptom in the last four weeks, 1= occasionally, 2= sometimes, 3= most of the time and 4= all of the time)

| **S.no** | **POP-Symptoms** | **0**  never felt symptom in the last four weeks | **1**  occasionally | **2**  sometimes | | **3**  most of the time | **4**  all of the time |
| --- | --- | --- | --- | --- | --- | --- | --- |
|  | A feeling of something coming down from or in your vagina? |  |  |  | |  |  |
|  | An uncomfortable feeling or pain in your vagina which is worse when standing? |  |  |  | |  |  |
|  | A heaviness or dragging feeling in your lower abdomen/tummy? |  |  |  | |  |  |
|  | A heaviness or dragging feeling in your lower back? |  |  |  | |  |  |
|  | A need to strain (push) to empty your bladder? |  |  |  | |  |  |
|  | A feeling that your bladder has not emptied completely? |  |  |  | |  |  |
|  | A feeling that your bowel has not emptied completely? |  |  |  | |  |  |
|  | Which of the symptoms above (questions 1–7) causes you most bother? | ……………. | | | | | |
|  | For how long have you felt one of the most chronic symptoms? | ……… | | | | | |
| **Patient Global Impression of Change questionnaire**  Ask the following questions only at end-line assessment (after intervention). Both the control and interventional group participants will be asked at the end of six months. | | | | | | | |
|  | How do you Perceive the change in prolapse since the start of the intervention? | | | | 1= Very much improved  2= Much improved  3= Minimally improved  4 = No change  5= Minimally worse  6= Much worse  7= Very much worse | | |

**Part-VI: Pelvic Floor Disability Index (PFDI-20)**

**Instructions:** Please answer all of the questions in the following survey. These questions will ask you if you have certain bowel, bladder, or pelvic symptoms and, if you do, **how much they bother you**. While answering these questions, please consider your symptoms over the last 3 months.

**Pelvic Organ prolapse Distress Inventory 6 (POPDI-6)**

| ***S.No*** | ***Do You…*** | **NO** | **Yes** | | | |
| --- | --- | --- | --- | --- | --- | --- |
|  |  | 0  **not present** | 1  **not at all** | 2 **somewhat** | 3 **moderately** | 4 **quite a bit** |
|  | Usually experience pressure in the lower abdomen? |  |  |  |  |  |
|  | Usually experience heaviness or dullness in the pelvic area? |  |  |  |  |  |
|  | Usually have a bulge or something falling out that you can see or feel in your vaginal area? |  |  |  |  |  |
|  | Ever have to push on the vagina or around the rectum to have or complete a bowel movement? |  |  |  |  |  |
|  | Usually experience a feeling of incomplete bladder emptying? |  |  |  |  |  |
|  | Ever have to push up on a bulge in the vaginal area with your fingers to start or complete urination? |  |  |  |  |  |

**Colorectal-Anal distress Inventory 8 (CRAD-8)**

|  | ***Do You…*** |  |  |  |  |  |
| --- | --- | --- | --- | --- | --- | --- |
|  | Feel you need to strain too hard to have a bowel movement? |  |  |  |  |  |
|  | Feel you have not completely emptied your bowels at the end of a bowel movement? |  |  |  |  |  |
|  | Usually lose stool beyond your control if your stool is well-formed? |  |  |  |  |  |
|  | Usually lose stool beyond your control if your stool is loose? |  |  |  |  |  |
|  | Usually lose gas from the rectum beyond your control? |  |  |  |  |  |
|  | Usually have pain when you pass your stool? |  |  |  |  |  |
|  | Experience a strong sense of urgency and have to rush to the bathroom to have a bowel movement? |  |  |  |  |  |
|  | Does part of your bowel ever pass through the rectum and bulge outside during or after a bowel movement? |  |  |  |  |  |

**Urinary distress Inventory 6 (UDI-6)**

|  | ***Do You…*** |  |  |  |  |  |
| --- | --- | --- | --- | --- | --- | --- |
|  | Usually experience frequent urination? |  |  |  |  |  |
|  | Usually experience urine leakage associated with a feeling of urgency, that is, a strong sensation of needing to go to the bathroom? |  |  |  |  |  |
|  | Usually experience urine leakage related to coughing, sneezing, or laughing? |  |  |  |  |  |
|  | Usually experience small amounts of urine leakage (that is, drops)? |  |  |  |  |  |
|  | Usually experience difficulty emptying your bladder? |  |  |  |  |  |
|  | Usually experience pain or discomfort in the lower abdomen or genital region? |  |  |  |  |  |

**Part-VII: The International Consultation on Incontinence Questionnaire**

**Instructions:**

Many people leak urine some of the time. This study is aimed to find out how many people leak urine and how much this bothers them. Please answer the following question, thinking about how you have been, on average, in the last four weeks.

| S.No | Questions | Responses |
| --- | --- | --- |
|  | How often do you leak urine? | 1. = never 2. = once a week 3. = 2-3 times a week 4. = once a day 5. = several times a day 6. = all the time |
|  | How much urine do you usually leak? | 0= none  2 = a small amount  4 = a moderate amount  6 = a large amount |
|  | Overall, how much does leaking urine interfere with your everyday life? (give a value between 0 (not at all) and 10 (a great deal) | 0 1 2 3 4 5 6 7 8 9 10 (encircle the appropriate number) |
|  | When does urine leak? Select all that apply to you | 0 = never leak  1 = before getting to the toilet  2 = leaks when coughing or sneezing  3 = leaks when sleeping  4 = leaks when exercising  5 = leaks when finished urinating and dressing  6 = leaks for no reason  7 = leaks all the time |

**Part VIII: Obstetric Fistula Screening Tool**

| Sr. No | Questions | Responses |
| --- | --- | --- |
|  | Have you ever given birth vaginally? | 0 = No 1= YES |
|  | Have you faced labor that lasted more than one day and night? | 0 =No 1= Yes |
|  | Do you have continuous leakage of urine? | 0 =No 1 =Yes |

**Part-IX: Pelvic Organ Prolapse Quantification (POP-Q)** (to be used by pelvic examiners)

**Instructions**: Measure the descent of all the nine measurement points in the POP-Q system. Measure all points at a maximum Valsalva, except Total Vaginal Length (TVL) which is measured at rest. Record measurements in centimeters to the nearest 0.5cm. TVL= is measured from Hymenal remnant to point D, Aa is an arbitrary fixed point on the anterior vaginal wall, which is 3cm back from the middle of the external urethral meatus in normal cases. Point Ba is the lowest part of the upper anterior vagina. This point is not a fixed point like Aa. It can be anywhere along the vaginal wall above the first 3cm (Aa). Point Ap and Bp are similar points to Aa and Ba but found on the posterior vaginal wall. GH is measured from external urethral meatus to hymenal remnant, PB from the hymenal remnant to the anal opening, C is the lowest part of cervix and point D is the insertion of uterosacral ligaments to the cervix. Both points are measured relative to the plane of hymenal remnants.

Height: ____________________ cm

Weight: ____________________ kg

Abdominal Striae: ­­­­­­­­­­­­­­ □ yes □ No

**POP-Q:**

| Aa = | Ba = | C = |
| --- | --- | --- |
| Gh = | Pb = | TVL = |
| Ap = | Bp = | D = |

...

Circumference of prolapse (cm): __________________________

Standing Cough Stress Test: □ positive □ negative Volume (mL):______

**PART-X: PROLAPSE RELATED QUALITY OF LIFE (FOR CONFIRMED POP-PATIENTS ONLY**)

**Instruction**: Below are some daily activities affected by prolapse. Please tell me how much your prolapse problem affected you (tick the correct column).

| **Domain** | **Items** | **0 Very good** | **1 Good** | **2 Fair** | **3 Poor** | **4 Very poor** |
| --- | --- | --- | --- | --- | --- | --- |
| Present general health (GHP | How do you describe your health at present |  |  |  |  |  |
|  |  | **0 Not at all** | **1 Slightly** | **2 Moderately** | **3 A lot** |  |
|  | Prolapse impact on life (PI) |  |  |  |  |  |
| Role limitations | To what extent does your prolapse affect your household task? |  |  |  |  |  |
|  | Does your prolapse affect your job/daily activity outside the home? |  |  |  |  |  |
| Physical/social limitations | Does your prolapse affect your physical activities? |  |  |  |  |  |
|  | Does your prolapse affect your ability to travel? |  |  |  |  |  |
|  | Does your prolapse affect your social life? |  |  |  |  |  |
|  | Does your prolapse affect your ability to see/visit friends? |  |  |  |  |  |
| Personal relationship | Does your prolapse affect your relationship with your partner? |  |  |  |  |  |
|  | Does your prolapse affect your sex life? |  |  |  |  |  |
|  | Does your prolapse affect your family life? |  |  |  |  |  |
| Emotions | Does your prolapse make you feel depressed? |  |  |  |  |  |
|  | Does your prolapse make you nervous/anxious? |  |  |  |  |  |
|  | Does your prolapse make you feel bad about yourself? |  |  |  |  |  |
| Sleep/Energy | Does your prolapse affect your sleep? |  |  |  |  |  |
|  | Does your prolapse make you tired? |  |  |  |  |  |
| Actions taken | Use pad to protect prolapse |  |  |  |  |  |
|  | Do you push up the prolapse |  |  |  |  |  |
| Severity measures | Pain/discomfort due to the prolapse? |  |  |  |  |  |
|  | Does the prolapse Prevent you from standing? |  |  |  |  |  |
| **Patient Global Impression of Improvement**  Ask the following questions only for end-line assessment (after intervention). Both the control and interventional group participants will be asked at the end of six months | | | | | | |
| Patient Global Impression of Change | How do you feel about your health after this intervention? | 1= Very much improved  2= Much improved  3= Minimally improved  4 = No change  5= Minimally worse  6= Much worse  7= Very much worse | | | | |

Thank you for your participation

# **ANNEX-III: Sidaamu Afoo Version Questionnaire**

**QA’MISO I: MASHALAQQETE QOOLA**

**HAWAASI YUNIVERSITE**

**HIKKIMINNUNNA FAYYIMMATE SAYINSE KOLLEEJE**

**Dagoomu Fayyimmate Rosi Mine**

**Xiinxallote Umo: Otootto Gobba Higge Fultino Amuwi Mitiimma Sidaamu Dagoomu Qoqqowi Mootimma, Itophiya, 2014 M.D**

**Xiinxallote Anni: Melese Siyumiho**

AYIIDDE KEEREHO!! Su’ma’ya _________________ yinannie; ani xiinxallote taje gamba asseemmohu Kalaa Melese Siyumihu qara xiinxallote aana ikkanna, iso riqiwe shiqoommo. Atera kaayyo iilliteennae atewa shiqoommo. Xiinxallote umi otootto gobba higge fultino amuwi aana xiinxallinanni. Doorroonnihehuno kaayyoteeti.

**Xiinxallote Goofimarchu Mixonke**

Xiinxallo illachishshannohuno: Amuwaho otootto gobba higge fultanno gede assannorichi maatiro bade afatenna noo mitiimma tirranni gedeeti, Sidaamu qoqqowi mootimmaraati. Ledoteno, ottootto gobba hige fultino amuwi sumuu yiiha ikkiro, woyyaabbino hospitaalla sonkanni hattono qarra anfe agadhinanni gedeeti.

**Xiinxallote Amuraati:**  Borri-xa’motenninna Fayyimmate ogeessinniiti mixote garinni hattono qara badateeti. Borri-xa’mote dawarokki borresseemmo. Tenne borri-xa’mo dawarate 40-50 xiqqeessaati. Tini borri-xa’mo amaddinohu ayimma, akata, miinja hattono ilate gadenna hattonno sirote bisi kifilla gobba higge fulturo leeltanno malaatta heeshshote aana abbitanno mitiimmaati. Goofimarchohono, dinye uyneennahe qajeeltino ogeeyyenni buuxo assinannihe mulekki no fayyimmate uurrinshara. Buuxote yannara mitiimma nooha ikkiro, lame gaamora beenkanni xaginsanni baycho keeshshatta.Tenne gaamora 5(onte) hinge buuxisiisi’nanni kaimunni (lamalate (0) layinki lamalanni leyikkitera(6) tonnikkitera(10) tonaa-ontikki lamalara(15), hodhishshu baatooshshe xiinxallasine baattanno.

**Xiinxallote Horo**

Xiinxallo amuwaho uytanno horo umikkihunni otootto gobba higge fultino amuwira baatooshshiweelo fayyimmate ogeeyyenni bunxanni. Ledoteno, hodhishshahono hatto yannakkira xiinxallote gaamonni baantannihe.

**Xiinxallote Beeqqo Lainohunni Baatooshshu Dino**

Xiinxallo abbitanno qarra lainohunni: onte (5) hinge buuxote ha'rate gobbaanni mittu qarri illishannohu dino. Ledoteno, buuxo asssinanni yannara teeda diwa hoogatenni injo macciishshama hoogate gobbaanni mittu qarri dino.Baxxitino kifilera teeda lowo geeshsha daheessine buuxo assinannita duu’ne kulleemmo; hattono fojo wolu mannira mittoreno dikullanni!

**Fojo Lainohunni**

Wo’manti taje kulattaeti, lowo geeshsha fojo wolu mannira dikuleemmo. Qole ate su’ma horontanni diborreeseemmo. Horontanni dawarootta dawaro ayeno diafanno.

**Xiinxallote Beeqqo Assattohu Sumuu Yiittaronna Mereeroho Xa’mo Xa’me Gudummokkino Agurate Qoossokki Agarantinote!**

Xiinxallote beeqqo assa hoogakki mittoreno amuwu afi’ranno owaante hooltannohetano di"ikitino/mereeroho dawarte gudittakkinni agurte ha'ra dandaatta.

Xa’mo heedhuhero woy xawinnohekkiri nooro, Melese Siyyumiho. Hawaasi yuniversitera loosaasincho xa tenne yannara xiinxallote aana xa’ma dandiitinanni

Bilbilu Kiirosi: 0912780577

**Mashalaqqete Qoola**

**Xinxxaloote horonna fojjo hitoonni agaraniro lowo geeshsha xawinsoonie dafira nna hattonno wodanchoomma .konni dafira ane summudinni sumuu yooma gede malaatiseemma!**

**Sumuu Yaattae Ballo!**

Dawaraancho/beeqqaanchu:

Malaate.............................. Barra........................

Taje gamba assanohu:

Malaate................................ Barra..................................

**QA’MISO II: SIDAAMU AFII GAANO**

**HAWAASI YUNIVERISITE HIKKIMINNUNNA FAYYIMMATE KOLEEJE**

Dagoomu Fayyimmate Rosi Mine

**Gafa Mite: Dawaraanchote Dagoomittete Haja Xa’mi’nanni Borri-xa’mo**

Worada_________ Ollaa________ Dargu Kiiro____________ Koodde_____ Mobayle______________________

| **A.K.** | | **Xa’mo** | **Dawaro** | **Sai** |
| --- | --- | --- | --- | --- |
|  | | Dirikki me’’eho ? | __________wo’mu dirrinni wori |  |
|  | | Teessokki hiikkooti? | - - - 1. Quchumaho       2. Baadiyye |  |
|  | | Adhammete Gara | 1. Leexa 2. Adhantinota 3. shiidhinota 4. tidhantinota 5. baxxitinote |  |
|  | | Ammanokki hiitteeti?  (dawarokki qoqqowi? | 1. Ortodokise 2. Pirotestaante 3. Musiliime 4. Kaatolike 5. Wole xawisi____________ |  |
|  | | Loosu Qeecha? | 1.. Minaama (mini ama )  2. Baatto loossi’re galinoha   1. Mootimmate Loosaasincho 2. Mootimmannita ikkitinokki uurrinshara 3. Halanyaho 4. Rosaanchoho 5. Wole xawisi____________ |  |
|  | | Hiikko ayiddeeti?/ Hiitte gosaati? | 1. Sidaama 2. Wolayitta 3. Amaara 4. Guraage 5. Oromo 6. Wole xawisi___________ |  |
|  | | Rosikki deerri hiikkonneeti?? | 1. Nabbawanna borreessa didandeemma 2. Nabbawanna borreessa calla dandeemma 3. Umi dirimi rosaanchooti 4. 2^ki^ dirimi rosaanchooti 5. 5. 2^ki^ dirimi aleenni rosinote |  |
|  | | Gashshaniki rosi deerri hikkonneeti?  Adhantinota ikkituro,(xa’mo 103 buuxxi) | - - - 1. Nabbawanna borreessa didandaanno  1. Nabbawanna borreessa calla dandaanno 2. Umi dirimi rosaanchooti 3. 2^ki^ dirimi rosaanchooti 4. 2^ki^ dirimi aleenni rosinoho |  |
| **Gaamo-II: Heeshshote Gade Xa’minanni Borri-xa’mo**  Konni woroonni noo borri-xa’mo , xa’mitto/a gedensaanni taaltino dawaro qoqqowi | | | | |
|  | Mereerimunni, ayyirrinore me’’e saate bayiratta barrunni (waa, lowo qaqqo , haqqe, kinchonna gide | | -------- sate |  |
|  | Sigaara wiliishshite egennootta? | | 1. Dee’ni 2. Ee |  |
|  | Maatekki giddo otoottote ayyara gobbara higge fultinohu no ? | | 1. Dee’ni 2. Ee |  |
|  | Keeshshitino busano buuse egennota sase lamallara aleenni ? | | 1. Dee’ni 2. Ee |  |
|  | Jawa shumara ofolata woyte Fula giwanohenni? (fulate qarrissanno) | | 1. Dee’ni 2. Ee |  |
|  | Mundeekki giddo sukkaare noohero buuxi’rootta? | | 1. Dee’ni 2. Ee |  |

**Gaamo III: Amuwu Ilaranna Otoottote Fayyimma Ledo Xaadinohunni Xa’minanni Borri-xa’mo**

| **A.k.** | **Xa’muuwa** | **Dawaaro** | **Sa’i** |
| --- | --- | --- | --- |
|  | Umikki yannara mine assi’roottahu meyikki dirikkiraati? | --------------- dirooti |  |
|  | Lamala agana ikkinokkiha, umo ka’e egennootanni? | 1. Dee’ni 2. Ee |  |
|  | Lamalu aganni gedensanni ilite eggenotanni? | 1. Dee’ni ___________ 2. Ee | sa’i 312 |
|  | Xa’mo303, ee. ikkiro umo ilitta woyite meyikki deerikkiraati? | --------------- dirooti  999……… diafoomma |  |
|  | Me’’e qaaqquulle ilitta? | ___________hige iloomma |  |
|  | Gedeniidi qaaqqo ilitta yannara meyikki dirikkiraati? | ---------- dirooti |  |
|  | Hitoonni ilte egennootta?  (Mittu aleenni doora dandiinanni ) | 1. Qarru noyikkiha iloomma 2. Godowa dadhatenni 3. Udiinnichu kaa’lonni iloomma |  |
|  | Mitto barra sae/(24) saate sae gamaa’mite egennoottanni? | 1. Dee’ni 2. Ee |  |
|  | Sa’u yanna giddo mine ilte egennootanni? | 1. Dee’ni 2. Ee |  |
|  | Gedensiidikki qaaqqo hiikko ilootta? | 1. Mine 2. fayyimmate uurinshara. |  |
|  | Gedensiidi qaaqqo ilittahu gedensaanni, meyikkita mini looso loosa hanafootta? | ----------- barrinni  ……….. lamalate gedensaanni |  |
|  | Godowa dadhite egennootanni? (qaaqqu fula giweennae shiima darga darroonnie, otoottote darroonnie ,danotenni darroonnie, godowi giddo darshinore holaara darroonnie) | 1. Dee’ni 2. Ee |  |
|  | Aganu mundeekki horontanni uurritinoni? (sasu aganni aleenni)? Maatete damboowishsha horoonsi’rittakkinni | 1. Dee’ni 2. Ee |  |
|  | Xa’mo 313, ‘’ee’’ yiittaro, mageeshshi yanna? | ------------- |  |

| **Gaamo -IV: Jajju woy Jirote Amadooshshe**  Konni worooninoo xa’mubba xa’mateni (dandaamiro la’atenni) gara ikkitino dawaro dooni. | | | | | |
| --- | --- | --- | --- | --- | --- |
|  | Maatekki agganno waa afidhannohu hiikkiinniiti? | Baambu waa :   1. Mini giddo baambu waa 2. Hoowete afamanno baambu waa 3. Dagoomu tuqi horoonsi’ranno baambu waa 4. Umme fushshinoonni waa | |  | |
|  |  | Agarooshshe:   1. Garunni agaraminoha 2. Garunni agaraminokkiha | |  | |
|  |  | Buete waa:   1. Garunni agarroonnita 2. Garunni agarroonnikkita | |  | |
|  |  | Daadanno Waa:  1.Lagu/garbu/kofaminoha/xashshuwa/ waa | |  | |
|  | Maatekki sagale loosi’ratenna anga hayishshi’rate horoonsidhanno waa maminni afidhanno? | Baambu waa:   1. Mini giddo baambu waa 2. Hoowete afamanno baambu waa 3. Dagoomu tuqi horoonsi’ranno baambu waa 4. Umme fushshinoonni waa | |  | |
|  |  | Agarooshshe:   1. Garunni agaraminoha 2. Garunni agaraminokkiha | |  | |
|  |  | Buete waa:   1. Garunni agarroonnita 2. Garunni agarroonnikkita | |  | |
|  |  | Daadanno Waa:   1. 1.Lagu/garbu/kofaminoha/xashshu/ waa | |  | |
|  | Way afamanno dargi hiikkooti? | 1. Meessi mini giddo  2.Meessi hoowe giddo  3. Wolu darginni | |  | |
|  | Waa dirri’ne hinganni yanna mageeshsha adhanno ? | 1. Daqiiqa/xu’eessa __________ 2. Dibuuxoommo/a | |  | |
|  | Sa’uta lame lamala giddo way bae barra wo’ma keeshshino? | 1. Dee’ni 2. Eewa 3. Diqaagamannoe | |  | |
|  | Way co’ichimma agarsiisate atewayinni agarrannire assoottori/tari no yite hedatta/o? | 1. Dee’ni ------------------------------------- 2. Eewa | | 4.8 | |
|  | Anganni way co’ichimma agara maa assa noohe?  Wo’manta hedo maareekki | 1. Gafe agatenni 2. Xagga woratenni 3. Hoccootunni ximbiiwaetnni 4. Waa ximbiimbanni meemonni (shaafa/shiimmaadda lubbuwa/etc.) 5. Arrishshote xawaabbinni xagisatenni 6. Mittowa kofatenni/ kuusatenni | |  | |
|  | Maatekki duucha yannara hiittooha shumate mine horoonsidhanno? | WAYINNI XAADINSOONNITA/ XAADINSOONNIKKITA   1. Baamba fanne horoonsi’nannita 2. Baambaho xaaddino way maashine 3. Ummooni balera waa horoonsi’ra 4. Base baalate waa horoonsi’ratenni 5. Shumate mini waa hiissine horoonsi’nanniro diafoommo/a?   BALE UMME QIXXEESSINOONNI SHUMATE MINE   1. Ayyare e’anno shumate mine 2. Bale umme suudinsoonni kinchinni 3. Bale umme afoo tu’noonnikkita 4. Mu’roote horonsi’nanni shumate mine. 5. Waa baaldete worreenna 6. Gottiima shumate mine   7.Injiinowi dino/dubbu giddo/xawoho | |  | |
|  | Tenne owaante woloota maate ledo horoonsidhinanni? | 1. Dee’ni 2. Eewa | |  | |
|  | Ate maate ledo mageeshshi mannaati konne shumate mine horoonsidhinannihu? | 1. 10 nni ajanno 2. 10 woy hakkuy ali 3. Afoommori/mari dino | |  | |
|  | Hakku shumate mini afmannohu hiikkooti? | 1. Mini giddo 2. Meessi hoowe giddo 3. Wole dargaati | |  | |
|  | Maatekki sagale loosi’rate hiittee gaaze horoonsidhanno? | 1. Korreentete wolqa 2. Du’nantanno gaaze 3. Kalaqamu gaaze 4. Addi addi keemikaalla 5. Laamba 6. Qitiissinoonni haqqe (kasale) 7. Haqqe 8. Kashee/hayissotenni 9. Gidu damatenni 10. Saadate obbinni 11. Sagale diloosi’neemmo | |  | |
|  | Sagale loosi’nannihu gallaniwaati, baxxitino kifileeti/ mini gobbaanniti? | 1. Mini giddooti 2. Baxxino mineeti 3. Barandahooti 4. Wolewaati | |  | |
|  | Sagale loosi’rate horoonsidhinanni baxxino mini noo’ne? | 1. Dinonke 2. Eewa | |  | |
|  | Minu galtinannihu ayeho? | 1. Ko meessiho 2. Baante gallanniho 3. Fiixunniho 4. Elunniho 5. Wole hee’riro xawisi_________________ | |  | |
|  | Konni mini giddo gonxanni kifilla me’’e no? | --------Kifilla no | |  | |
|  | Minu giddoydo iimiidi raga(korniise) mayinni biifinsoonniho? | 1. Kalaqamu haqqinni (biinfori dino, sabbunni,nna kandoonni sabbinni) 2. Calla anfi gede assinnoonniha (xu’minsoonnikki saattinni/plastic shara, shomboqqotenni/leemmichunni, haqqu xaawulinni, and komborsaatunni) 3. Xu’minsoonnikorniise (siwiilunni/culku siwiilinni, haqqunni,alumineemetnni/simintotenni | |  | |
|  | Mini uulliidi loosaminohu mayinniiti? | 1. Kalaqamunni noo gedeenni nooho (baatto/ bushshaho) 2. Safo tunge doogo hunnoonniho (haqqu xaawula, nna shomboqqotenni/ leemmichunni) 3. Xu’minsoonni mine (shakilunni) (haqqu xaawulinni xu’minsoonniha, uullano iimano shakila, simintotenni, alumineemetenni, uulla karranni shara(minxaafe) | |  | |
|  | Minu anni / ati saadate hoshsho, baatto hawuurranni saadanna lukkuwa ceo noosiho? | 1. Dee’ni 2. Eewa | |  | |
| - - 1. 4 | Aliidi saada giddonni minu annihu mageeshshaati?  Mitturi nokki ikkiro(00) wori.  95 tu woroonna aleenni ikkiro (95) wori.  Kiiro anfonikkiha ikkiro(98) wori | 1. Saadanna bootta_______________ 2. Wole saada______________ 3. Farado/harre/gaangootta ____ 4. Gaala________ 5. Me’’e _________ 6. Ge’reewo _________ 7. Caacurre/ lukkuwa ______ 8. Diishshote koshsha | |  | |
| - - 1. 421 | Saada galtannowi baxxino dargi noo’ne? | 1. Dee’ni 2. Eewa | |  | |
| - - 1. 422 | Maate’ne giddo loosi’nanni baatto afi’nohu no? | 1. Dee’ni ------------------------------------- 2. Eewa | | 4.24 | |
| - - 1. 423 | Minu annira me’’e hekitaare ikkitannoti loossi’nanni baatto noosi? | ___________Hekitaare | |  | |
| - - 1. 424 | Konni worooni noori giddo mini’ne giddo afamanohu hiiko neeti? (mittete aleeni doora dandiinanni) |  | Eewa(1) | Dino (0) |  |
|  |  | 1. Korreente---------- 2. Raadoone ----------- 3. Telewishiine -------- 4. Mine silke---------- 5. Kompiitere ---------- 6. Mitore qiissanno udiinnichi --- 7. Xarapheezzu -------- 8. Barcimu ------------- 9. Shiwote daallasi---- 10. Korreentete wolqanni sagale raisi’nanni mixashsho ----- 11. Laambunni/shaamu/ ------ 12. Faanoose------------- |  |  |  |
|  | Maatekki giddo togoo udiinni no: |  | Ee  (1) | Dino  (0) |  |
|  |  | 1. Tolobishiine -------- 2. Bilbilu------ 3. Shalleette------------ 4. Xexxerrisu------ 5. Saadate gaare- 6. Kaameelu/ hogowi kaameeli --------- 7. Yowolo motor nooti----- 8. Sasu goommi baajaaje ------------ |  |  |  |
| - - 1. 426 | Maatekkira baankete akkowaante kiiro noonsa? | 1. Dinonsa 2. Eewa | |  | |
| - - 1. 427 | Maatekki giddo shiimmaadda daddalu uurrinsha akkowaante kiiro noonsa? | 1. Dee’ni 2. Eewa | |  | |
|  | Maate’ne giddo sigaara wiliishshaahu barrunni me’’e higeeti?  Barrunni/lamalatenni/ aganunni/ dirunni / takkonta? | 1. Barrunni 2. Lamalatenni 3. Aganunni 4. Aganunni mitteege 5. Takkonta | |  | |

**Gaamo-V: Otootto Gobbara Higge Fulturo Leellanno Malaatta Buunxanni Borri-Xa’mo**

**Hajajo:** Konni woroonni shiqqino lamala borri-xa’mo illachishshannohu otootto gobbara higge fultino amuwira xa’minanninna bikkinanni xa’muuwaati. Doorshu noohu ( 0) kayse 4 geeshshaati. Muxxe tenne xiinxallo beeqqaancho, sa’u shoole lamallara me’’e hige malaatu macciishshamihero ballo eeggatena kulie? (keennanni gari : 0= horontanni dileellinoe malaatu sa’u shoole lamallara,, 1= keeshshe, 2= sae sae 3=roore yannanna 4= wo’ma woyte)

| **A.k** | **Leeltanno malaatta** | | **0**  Horontanni dileellanno | **1**  Keeshshe keeshshe | **2**  Sae sae | **3**  Roore yanna | 4  Wo’ma yanna |
| --- | --- | --- | --- | --- | --- | --- | --- |
|  | Ikkinorichi otoottote widoonni hige daynoha lawe macciishshaminoheni? | |  |  |  |  |  |
|  | Otootto xissitannohenni roorenkanni uurrutta yannara ? | |  |  |  |  |  |
|  | Mudukku woroonni ayirrannoherichi macciishshamannoheni? | |  |  |  |  |  |
|  | Hallikkira wori adda duunaminori woy ayirrinori macciishshame egenninoheni? | |  |  |  |  |  |
|  | Way shuma daggukkinni niixissannoheni? | |  |  |  |  |  |
|  | Way shuma gooffinokkiha lawe macciishshamannoheni? | |  |  |  |  |  |
|  | Jawiidi shuma gooffinokkiha lawe macciishshamannoheni? | |  |  |  |  |  |
|  | Aleenni xawinsiri giddo roorenkanni mittinsino qarri hiikkonneeti? (xa’mo 1–7) geeshsha | | ……………. | | | | |
|  | Mageshshi yanna mittu malaati macciishshamihe roorenkanni seeda yannara? | | ……… | | | | |
| **Kalqete Dhiwamaasinetenni Afi’nanni Babbaxxitino Borri-Xa’mo**  Konni woroonni no borri-xa’mo xa’minannihu goofimarchohooti. Xa’minannihu dhibbu malaati nooriranna nookkihuraati. Xa’minanni yanna lewu(6) agani gedensaanniiti | | | | | | | |
|  | Xagisi’ra hanafittankunni may soorro leeltinoha lawannohe? | 1= lowo geeshsha huroomma  2= woyyate  3= dhibbu ajanni ha’rinoe  4 = mitte soorro dino  5= shiima geeshsha xissanni nooe  6= xissanni nooe  7= lowo geeshsha xissanni nooe | | | | | |

**Gaamo-VI: mudukkunna sirote bisi qarqari bisu xe’ne daafira xa’manni borri-xa’mo (PFDI-20)**

**Hajajo: Konni woroonni noo xa’mora dawaro dawari; ha’runsote/buuxote xa’mo gedensaanni**. Xa’minannihu helleete ,ufuuffaate, sirote bisi qarqari malaattanna mageeshshi geeshshi mittimmansa, buunxanni borri-xa’mo. Dawarratta woyte sasu(3) agani aleenni ikkinoheri qaagi!. **0 = dino malaatu, 1= wo’manka woyte dikkino,** 2**=shiima yanna 3 =mereerimunni nna 4 = horontanni shiima yanna**

**Mudukkunna sirote bisi qarqari bisu xe’ne daafira xa’minanni borri-xa’mo (PFDI-20)**

| 1. ***K*** |  | **Dee’ni** | **Ee** | | | |
| --- | --- | --- | --- | --- | --- | --- |
|  |  | 0 dino malaatu | 1 wo’manka woyte dikkino | 2 shiima woyte | 3 mereerimunni | 4 horontanni shish  woyte |
|  | Godowikkira woriidi qarqarira woroo qole xiwannori hee’ranno? |  |  |  |  |  |
|  | Siimu qarqarira ayirre macciishshame egenninohe? |  |  |  |  |  |
|  | Otoottote widoonni ikkinorichi fulanni nooha lawannohe gede ikkannohe? |  |  |  |  |  |
|  | Otoote woy jawa shuma fultanno qoxxeessa xiwannori no gede macciishshamannohe? |  |  |  |  |  |
|  | Way shuma gooffinokkiha lawe macciishshamannohe? |  |  |  |  |  |
|  | Shumaa’ratta yannara xiwannoheri nooha lawannohe otoottokkira/qubbichikkinni kisse egennoottanni? |  |  |  |  |  |

**Helleetenna Xuuxxote Xiwani Daafira Xa’minanni Borri-Xa’mo (CRAD-8)**

|  |  |  |  |  |  |  |
| --- | --- | --- | --- | --- | --- | --- |
|  | Jawa shumara ofolatta woyte niixisannoheni? |  |  |  |  |  |
|  | Jawa shumara ofolte ka’eennano gooffinokkiha lawe macciishshamannoheni? |  |  |  |  |  |
|  | Jawa shuma ate hajajo gobbaanni fultannoheni? |  |  |  |  |  |
|  | Deeo ikkiro, ate hajajo gobbaanni fulannohe? |  |  |  |  |  |
|  | Fooleessu /fuuto ate hajajo gobbaanni fultannoheni? |  |  |  |  |  |
|  | Jawa shumara ofollatta woyte xissannoheni? |  |  |  |  |  |
|  | Jawa shuma mudditannoheni lowo geeshsha ? |  |  |  |  |  |
|  | Hellee gobbara higge fultannoheni deeu gedensaanni? |  |  |  |  |  |

**Way Shuma Mitiimma Daafira(UDI-6)**

|  |  |  |  |  |  |  |
| --- | --- | --- | --- | --- | --- | --- |
|  | Wayi shuma shumaa’ratta yannara macciishshamannoheri nooni? |  |  |  |  |  |
|  | Shuma ciphi-ciphi yitannohe muddite? |  |  |  |  |  |
|  | Shuma ciphi-ciphi yitanno, buusatta, hanxishshi’rattanna woy oso’litanna? |  |  |  |  |  |
|  | Shiima shuma ciphi-ciphi yitanno)? |  |  |  |  |  |
|  | Way shuma shumaadhe kaittarono gooffinokkiti macciishshantannohe? |  |  |  |  |  |
|  | Mudukku woridinna siimu qarqarira fayyimma dimacciishantannohe? |  |  |  |  |  |

**Gaamo-VII: Kalqete Shuma Cophi Yitanno Gara Xiinxallinanni Gade Borri-xa’mo**

**Hajajo:**

Lowo manni way shuma ciphi-ciphi assanno. Tenne xiinxallo umi mageeshshi manni shumansa ciphi- ciphi assannonna mittii’manno yaateeti. Ballo, sa’u shoole lamallara me’’e hige ciphi -ciphi assittaro hedi mereerimunni?

| A.K | Xa’mo | Dawaro |
| --- | --- | --- |
|  | Me’’e higge way shuma ciphi yaatta? | 1. =horontanni 2. = mitte hige lamalate gido. 3. = 2-3 geeshsha mitte lamela gido. 4. = mitte hige barrunni. 5. = Duucha hige mittu barri giddo 6. =wo’ma woyte. |
|  | Mageeshshi shuma ciphi assatta? | 0 = horontanni  2 = shiima  4 = Mereerima.  6 = lowota |
|  | Xaphoomunni, mageeshshi shuma barrunni ciphi assatta? (0 nna 10 mereero qoqqowi | 0 1 2 3 4 5 6 7 8 9 10 (taltino dawaaro qoqqowi.) |
|  | Mamoote way shuma ciphi yitannohehu? (taltino dawaaro dori) | 0 =horontanni diyitanno  1 = shumate minira ha’rammara albaanni.  2=buuseemma/hanxishshi’reemma yannara  3 = goxe he’reenna.   1. = looso looseemma woyte.   5 Shumaa’re kaeemma woytenna uddi’re kaeemma woyte  6 =Korkaatiweello.  7 = Wo’ma woyte. |

**GaamoVIII: Fesitullu Qarri Noo Amuwira Xa’minanni Borri Xa’mo**

| A.K | Xa’mo | Dawaro |
| --- | --- | --- |
|  | Keeraancho ila ilte egennootta? | 0 = Dee’ni 1= Ee |
|  | Mitto barranna hashsha sa’e gama’mite egennootanni? | 0 =Dee’ni 1= Ee |
|  | Duucha woyte ciphi yitannohenni? | 0 =Dee’ni 1 =Ee |

**Gafa-IX: Otootto Gobba Higge Fultinotta Bikkinanni Gade (POP-Q)** (horoonsi’nannihu hikkimannaahooti)

**Hajajo:**:Honsu gari bikkote gade noose (POP-Q). mannimmate guunte/biso milli assinanni loossinsanni bikkinanni. Bikkinannihu seentimetiretenniiti

Hojja: ____________________ cm

Qelpheepho/ayirrinye: ____________________ kg

Godowaho malaatu leella: ­­­­­­­­­­­­­­ □ Ee □ Dee’ni

**POP-Q:**

| Aa = | Ba = | C = |
| --- | --- | --- |
| Gh = | Pb = | TVL = |
| Ap = | Bp = | D = |

Otoottote doycho sentimetirenni wori! __________________________

Uurrite bussanni hedhenanni bikkinonni bikko: □ nose □ dinose qelpheepho(mL):______

**Gafa-X: Otootto Gobbara Fulturo Hiittoo Heshshote Gade Nooro Bunxanni Borri- Xa’mo (Otootto Gobbara Fultino Amuwira Calla)**

**Hajajo**: Konni woroonni noo malaati leellannohu otootto gobbara higge fultino amuwira leellanno malaateeti. Taaltino dawarora malaatisi.

| **Amado** | **Xa’muwa** | 0  Lowo geeshsha danchaho | 1  danchaho | 2  duushshanno | 3  diduushshanno | 4 lowo geeshsha didushshanno |
| --- | --- | --- | --- | --- | --- | --- |
| XAPHOOMU FAYYIMMA(GHP) | Fayyimmakki hiittoonni xawisatta? |  |  |  |  |  |
|  |  | **0 wo’ma woyte dikkino** | **1 shiima yanna** | **2 mereerimunni** | **3 lowo geeshsha** |  |
|  | Otootto gobbara higge fulate mitiimma heeshshokkira. |  |  |  |  |  |
| MINI LOOSO LOOSA HOOGA HOGGAolhlimitations | Otootto gobba higge fultinohe dafira, mini loosi hittoo mitiimma abbinohe? |  |  |  |  |  |
|  | Otootto gobba higge fula ,mini gobbayidi loosikkira mitiimma abbinohe? |  |  |  |  |  |
| Mannu noowa ha’ra hoogate qarra | Otootto gobba higge fulte milli yaattata mitiinsinoheni? |  |  |  |  |  |
|  | Otootto gobbara higge fultannohe baychunni baycho hodhate mitii’mitanni? |  |  |  |  |  |
|  | Otootto gobba higge fultino daafira,dagoomu ledo xaadate mitii’mitta? |  |  |  |  |  |
|  | Otootto gobba higge fultinohe daafira miillaki ledo xaadate mitii’mitta? |  |  |  |  |  |
| Mannu ledo gade ledo | Otootto gobba higge fultinohe daafira,maatekkira mitiimma abbinoheni? |  |  |  |  |  |
|  | Otootto gobba higge fultino daafira siimu xaadooshshira mitiimma abbinohe? |  |  |  |  |  |
|  | Otootto gobba higge fultino daafira,maatekkira mitiimma abbinohe? |  |  |  |  |  |
| Akata | Otootto gobba higge fultino daafira,dadillu macciishshamannoheni? |  |  |  |  |  |
|  | Otootto gobbara higge fultino daafira caaccawaatani? |  |  |  |  |  |
|  | Otootto gobba higge fultino daafira ate bunshe macciishshantannoheni? |  |  |  |  |  |
| Goxichu/wolqa | Otootto gobba higge fultino daafira goxatta woyte mitii’mitta? |  |  |  |  |  |
|  | Otootto gobba higge fultinohe daafira bunshe calla macciishshantannohe? |  |  |  |  |  |
| Marichi qaafo adhita?taken | Hocoota/pade horonsiraata. |  |  |  |  |  |
|  | Otootto gobba higge fultanno yannara xiibbe egennootta? |  |  |  |  |  |
|  | Otootto gobba higge fultino daafira xisso/injaa hooga maciishshantanoheni? |  |  |  |  |  |
|  | Otootto gobba higge fultinohe daafira uurrate mitii’mattani? |  |  |  |  |  |
| **Kalqete Dhiwamaasinetenni Afi’nanni Babbaxxitino Borri-Xa’mo**  Konni woroonni no borri-xa’mo xa’minannihu goofimarchohooti. Xa’minannihu dhibbu malaati nooriranna nookkihuraati. Xa’minanni yanna lewu(6) agani gedensaanniiti | | | | | |  |
| **Kalqete Dhiwamaasinetenni Afi’nanni Babbaxxitino Borri-Xa’mo** | Mamoote way shuma ciphi yitannohehu? (taltino dawaaro dori) | 1= lowo geeshsha huroomma  2= woyyate  3= dhibbu ajanni ha’rinoe  4 = mitte soorro dino  5= shiima geeshsha xissanni nooe  6= xissanni nooe  7= lowo geeshsha xissanni nooe | | | |  |

# **ANNEX-IV: Lifestyle Counselling and Pelvic Floor Muscle Training Protocol**

**What is a pelvic organ prolapse and what are the symptoms?**

Pelvic organ prolapse, often known as 'prolapse,' is a vaginal alteration in which a pelvic organ, such as the bladder, bowel, rectum, or uterus, slips downwards in the vagina, creating the sensation of "something coming down" or "vaginal heaviness." The protrusion might be felt on either the inside or outside of the vaginal wall. Bladder, bowel, and sexual issues can all be caused by prolapse. The pelvic organs - the bladder, vagina, uterus, and rectum - are held and maintained inside the bony pelvis by ligaments and muscles, which are collectively known as the "pelvic floor.”

To prevent incontinence, the pelvic floor muscles support the pelvic organs and manage the bladder and bowel. The pelvic organs can bulge into the vagina, forcing the vaginal walls to slip downwards, resulting in a prolapse, if either the pelvic floor muscles or the supporting structures fail. A prolapse might be minor, providing little or no discomfort, or severe, causing several issues and negatively impacting your quality of life. Whether the downward bulge is felt or visible within the vagina or can be felt or seen outside the vaginal entrance may affect how much the prolapse interferes with your life. Prolapse might vary from day to day and even within a single day. On some days, you may be completely unaware of your symptoms, while on others, you may be acutely conscious of them; it may be alright in the morning but very uncomfortable later in the day. The sensation of’ something coming down' or of vaginal heaviness/bulge is the most typical symptom. Other common bladder and bowel symptoms can be caused by a prolapse, but they may also be unrelated.

**What causes pelvic organ prolapse?**

Prolapse is caused by a number of events that disrupt the vaginal support system. The factors linked to pelvic organ prolapse are mentioned below, along with a brief explanation of why they are linked.

**Pregnancy and childbirth**: This is the most prevalent cause, especially if the delivery was vaginal. The growing baby puts pressure on the pelvic organs and pelvic floor muscles, while hormonal changes through pregnancy lead the supporting ligaments to relax in preparation for birth. An episiotomy, a vaginal tear, or a forceps assisted delivery can weaken the supporting system, resulting in complications at the moment of delivery or later in life. Pelvic floor muscle workouts can help you maintain the strength of your pelvic floor muscles while you're pregnant.

**Obesity and being overweight**: the more weight your body has to manage, the more pressure is exerted on the pelvic organs and through the pelvis. A significant weight gain might place additional strain on the supporting structures, increasing the risk of prolapse.

**Heavy lifting**: If your day-to-day activities include heavy lifting or frequent bending, the increased abdominal pressure pushing downwards may raise the risk of a prolapse, especially if your pelvic floor supports are in sufficient.

**Family history**: A strong family history of pelvic organ prolapse, such as your mother or sister, may enhance your chances of developing a prolapse. This is assumed to be owing to a weak collagen type.

**Age**: As you get older, you're more likely to get a prolapse. Muscle strength may deteriorate as a result of aging, along with other changes.

**Menopausal changes**: As your levels of vaginal estrogen drop, some of the hormonal change that accompany menopause might exacerbate prolapse symptoms. The reduced amount of vaginal estrogen after menopause may impact prolapse symptoms.

**Constipation**: constipation and straining to empty your bowels might increase the risk of prolapse or worsen the existing prolapse. Increased intra-abdominal pressure is directed down onto the pelvic floor, causing strain and tension.

**Chronic cough:** If you have asthma or other illnesses that cause you to cough frequently, you may be at risk for a prolapse. This is caused to the pelvic floor being strained by recurrent increases in intra-abdominal pressure.

**Previous pelvic surgery**: If you've already had a prolapse repair or a hysterectomy, your chance of getting another prolapse are higher.

**What can you do to help a pelvic organ prolapse?**

Pelvic organ prolapse is not a life-threatening disorder, and not all prolapses deteriorate; in fact, some may improve. If you have been told you have a pelvic organ prolapse, you might want to consider the following options:

• Do nothing and wait for your symptoms to improve.

• Develop healthy bladder and bowel habits

• Change your lifestyle to lessen downward pressures in your pelvis, especially if any of your current activities aggravate your symptoms, such as weight reduction or avoiding or reducing the amount of lifting you do.

• Increase the vaginal support by strengthening your pelvic floor muscles.

• Consult a gynecologist about surgery as a possibility (or choice).

If your prolapse is not bothersome, you may decide to do nothing. However, research suggests that completing pelvic floor muscle exercises and following the recommendations below can help prevent your prolapse from worsening.

**Bowels**

- It's critical to avoid constipation since it puts additional strain on the pelvic floor muscles, which can exacerbate prolapse symptoms.
- Eating a healthy diet rich in fruits, vegetables, and fiber can assist.
- Make sure you're getting enough water as well (between 1.5 to 2 liters of fluid per day).

**When it's time to leave, try to adopt appropriate toilet habits**:

- - Don't overwork yourself
  - Sit fully on the toilet, rather than hovering; this will aid in the relaxation of the correct muscles.
  - Stand with your feet apart and your arms comfortably resting on your legs, supported by a seat or support.
  - Don't tighten your abdominals; maintain your tummy relaxed.
  - Avoid holding your breath; instead, aim to maintain a calm breathing pattern.
  - Sit fully on the toilet, rather than hovering; this will aid in the relaxation of the correct muscles.
  - Applying mild pressure vaginally on the bulging wall toward the rear canal may assist in more thoroughly and successfully emptying the bowels.

**Bladder**:

- Instead of going to the bathroom 'just in case,' go when your bladder needs to be emptied
- Sit fully on the toilet, rather than hovering; this will aid in the relaxation of the correct muscles.
- You might find it beneficial to lean forward and back numerous times after passing urine to help make sure that all the urine comes out
- do not push or strain to empty your bladder; you might increase the prolapse
- Try not to lower your daily fluids (1.5 – 2 liters per day is recommended); you may aggravate the prolapse if you push or strain to empty your bladder.
- The number of times you need to empty your bladder may not always be reduced by drinking less.

**Intercourse**

- Having sex with a prolapse is perfectly safe and will not harm or exacerbate the bulge.
- To aid with vaginal dryness or discomfort with penetration, consider alternative positions for sexual intercourse and use a suitable lubricant.

**Weight**:

- Being overweight causes the pelvic floor muscles to be overworked.
- If you reduce weight, your symptoms may improve.

**Taking steps to lower your intra-abdominal pressure**

**Reduce your cough:** If you have a chronic cough, make sure you take medications as directed and drinking plenty of water.

**Lifting**: It is not just the weight of the object you are lifting that can aggravate your symptoms. Bending and lifting on a regular or recurrent basis might also be a concern.

• Develop a good lifting habit for all chores, including light ones. Before each lift, tighten your   pelvic floor muscles.

• Splitting weights for carrying, making more frequent journeys, and lifting correctly when taking something from low down are all good ideas.

**Exercise and activities**: Avoid activities that aggravate symptoms, like prolonged standing.

Whenever feasible, try to divide your day into shorter intervals of standing with moments of sitting in between. Jumping, jogging, or aerobics (any activity that requires both feet to be off the ground at the same might aggravate your symptoms, especially if your pelvic muscles are weak.  Weight-bearing activities might sometimes put too much downward pressure on the body. Low-impact exercise like modified aerobics, cycling, a cross trainer, quick walking can be substituted.

**INSTRUCTIONS: THE FOLLOWING PIECE OF COUNSELING IS ONLY FOR WOMEN WHO ARE PART OF THE INTERVENTIONAL CLUSTERS.**

**Increasing the strength of your pelvic floor muscles**

To support the pelvic organs, the pelvic floor muscles operate as a hammock. PFM exercise will strengthen and retain the muscles to be more effective at supporting the pelvic organs, reducing prolapse symptoms. Pelvic floor muscle exercises (should comprise both long, held squeezes and brief, rapid squeeze, with each squeeze ending with complete relaxation of muscles. To assist the muscles get stronger and more effective, you should work them until they tire and repeat the exercise on a regular basis.

These exercises can be done in any position, however if your prolapse extends beyond the vaginal opening, you may find it easier to do them lying down.  If your prolapse isn't too bad, you might be able to complete these exercises while sitting. Inside, you should feel a squeeze and lift. Avoid holding your breath or squeezing other muscles like your legs or buttocks.

**Pelvic Floor Muscle Exercises**

Aim for 10 long squeezes, each held for 10 seconds, followed by 10 short squeezes.

- - If you can only hold the squeeze for a short period, you may need to start with ‘little and frequently’.
  - Perform pelvic floor muscle exercises at least three times per day.
  - When you are seated or lying down, you may find it easier to begin your routine.
  - Gradually increase your fitness program throughout the weeks and months.
  - After 3 to 5 months, you should notice a difference and should continue to practice your pelvic floor exercise once a day to keep the progress.
  - As your muscles strengthen, try doing your exercises in different positions, such as standing.
  - Eventually, you will be able to practice using these muscles while walking and bending.
  - Exercises for the pelvic floor muscles must be performed correctly.

# **ANNEX-IV: Sidaamu Afii Version of Lifestyle Counselling and Pelvic Floor Muscle Training Protocol**

**HALLU WORI BISI KIFILERA UYNANNI QAJEELSHINNA HEESHSHOTE GADE AMADO**

**Otootto gobbara higge fultanno yaa mayyaate? Leellishshanno malaatta hiikkuriiti?**

Otootto gobbara higge fula yaa:Amuwaho sirote bisi kifilera ufuuffa,otootto,hellee gobbaanni leelturo ,gobba higge fultino yine woshshinanni.Hattono sirote bisi kifilera ayirrino gede macciishshamirooti. Aleenni xawinsiri bisu kifillanni gobba higge woy giddoonni hee'renni malaate leellishsha/macciishshamme heedhurooti.

**Marichooti otootto, helleenna ufuuffa sirote bisi kifilla widoonni gobba higge fultanno gede assannori?**

**Lowo korkaati no kuriuu abbannori,Sirote bisi kifilera lowo maali (xunchi) biso irkise amadannohu mimmituwiinni baxxa hanafirooti roorenkanni togoori ikkannohu.**

Godowa (godowu gata) nna qaaqqo ila: Roorenkanni godowanna qaaqqo sirote kifile widoonni ila abbitanno,qaaqqu godowamiro godowu giddo lophanni ha'ranno.Lophanni ha’ranno yannara sirote bissa aana lowo xiiwo kalaqanno.Hattono muje (hormone) godowu gatanno yannara cingiitanno gari soorramannohuraati ilate bisu kifilla qixxeessate kalaqamu ortenni. .

**Danna saino maqee lexxanna du’ma:** danna saino du’milli halli wori bisu kifiilla irkisanno maalira xiiwo ledirooti. .

**Ayirrinoricho bayi'ra/duha**:Ayirrinoricho barru barrunni bayi'ra bisinke aana qoldissanno(godowu maalira) xiiwo leddanno daafira konne qarra abbanno.

**Fiixaho kuni qarri hee'riha ikkiro:** lawishshaho muli fiixira (amakkira/rodookkira) tini mitiimma heedhuha ikkiro heedhara dandaanno.

**Diro**: diru lexxanni ha'ri kiiro tini mitiimma lexxitanno..

**Ilate yanna sa’'a** : ilate yanna sa'’a tenne mitiimmarano qarra abbitanno.

**Godowu moolle**: jawa shumara ofollinanni yannara godowu mooleenna niixa konni qarrira xaadisanno..

Keeshshitino buusano : Asimete dhibbi woy buusiisanno dhibbi konni qarrira tuganno.

Mudukku woroonni godowa dadhiniro:mudukku woroonni godowa dadhinirono konne qarra abbitanno(otootto dadha qarra leddanno.):

**Maricho assa hasiissanno bisu kifilla gobba higge sirote bisi kifilenni fultuha ikkiro?.**

:

- Godowu moolannokki gede assi'ra.
- Haanxe afidhino sagale saga’la,gummanna laalo ita.
- Xalala waa aga (1.5-2) litire aga..

**Jawa shuma ofollinanni yannara qoropho assa.**:

- - Umikki fajjo assittoti shumatta ofolatta yanna:
  - Shumate ofolatta yanna ofoolla hasiisanno,qupphi ya gawajjo abba danditanno.
  - Ofollatanna uurrata yanna ragunni ofolla hasiissanno..
  - Godowakki usudha dihasiissanno.
  - Foolatta woyte foolekki giddo qolte amada hooga

**UFUUFFATE KEERAANCHIMMA AGADHA**:

- Way shuma daganno yannara calla ha'ra.
- Shumaa'ratta woyte ofolte shumaa'ra.
- Shumaa'ratta yanna niixa hooga.
- Ikkado waa barrunni aga (1.5 – 2) litire aga
- Keeraanchimma agadhate ikkado/ajishate waa aga.

**SIIMU XAADOOSHSHI YANNARA.**

- Otootto fultanno yannarano siimu xaadooshshi assa qarra diabbitanno woy qarra dileddanno.
- Bisu moolannokki gede qufisannoricho horoonsi'ra(sirote bisi kifilera)

**MAQEE LEXXA HOOGA**

- Danna sa’ino du’ma hooga.
- Maqee lexxa hooga konni qarrira ditugganno..

**Godowikkira xiiwo ledannore agura.**

**Buusano ajisha:** buusanote hikkiminna assi'ranna buunsanni yannara co'icha waa aga..

**AYIRRINORE BAYI'RA HOOGA**: shotare calla kaysa qarraho ditugganno.

• Bayi'rattote yannara umo sekke agadha hasiissanno.

**Mannimate guuntenna loosu yannara umo agadha:lowo yannna uurra hooga hattono loonsanni looso saatetenni beenke loosa.**

HAJAJO**: konni woroonni noo amaale uyinannihu mitiimma leeltino amuwira xaginsanni gaamoraati**

**Halli wori mannimaate guunte loosa.**

Bisokki 10 higge seeda duu'ra, xaano harancho yanna 10 hige duu'ra.

- - Bisokki harancho yanna calla duu'rattaha calla ikkiro,mule mule manninnaate guunte loosa hasiissanno.
  - Halli wori mannimmate guunte barrunni ajaanni ajeenna sase hige loosa hasiissanno.
  - Ofolatta yannanna haawaatta yannara shotunni ka'’a hasiissanno.
  - Suutunni mannimmate guunte jawaante leda hasiissanno(lamalatenni agannate giddo).
  - Sasunni ontu agani geeshshi gedenssanni may badooshshi nooro bade afanna barrunni mitte hige loosa hasiissanno hurre lexxitanno gede.
  - Mannimate guuntenni maalikki jawaatinoha ikkiro,mannimate guunte addi addi danita loosa lawishshu gede(uurra).
  - Goofimarchohono,ha'ramatenninna bisokki qoldisatenni mannimmate guunte loosa

Halli wori mannimmate guunte ragunni loosa hasiissanno, maala(xuncha) kaajjishi'ratenni.
