# Supplementary material for: Effect of midwife-led pelvic floor muscle training on prolapse symptoms and quality of life in women with pelvic organ prolapse in Ethiopia: A Cluster-randomized controlled trial
Source: PLoS Med. 2025 Mar 31;22(3):e1004468. doi: 10.1371/journal.pmed.1004468 (PMC11977982; doi:10.1371/journal.pmed.1004468)
Supplement: S4 File — (DOCX) [file pmed.1004468.s004.docx]

**CONSORT for Abstract**

**Checklist: items to include when reporting a randomized trial in a journal**

**or conference abstract**

| **Item** | **Description** | **Sections** |
| --- | --- | --- |
| Title | Identification of the study as randomized | Title section |
| Authors^a^ | Contact details for the corresponding author | Title page |
| Trial design | Description of the trial design (e.g., parallel, cluster, noninferiority) | Abstract paragraph 2 |
| **Methods** |  |  |
| Participants | Eligibility criteria for participants and the settings where the data were collected | Abstract paragraph 2 |
| Interventions | Interventions intended for each group | Abstract paragraph 2 |
| Objective | Specific objective or hypothesis | Abstract paragraph 1 |
| Outcome | Clearly defined primary outcome for this report | Abstract paragraph 2 |
| Randomization | How participants were allocated to interventions | Abstract paragraph 2 |
| Blinding (masking) | Whether participants, care givers, and those assessing the outcomes were blinded to group assignment | Abstract paragraph 2 |
| **Results** |  |  |
| Numbers randomized | Number of participants randomized to each group | Abstract paragraph 3 |
| Recruitment | Trial status | Abstract paragraph 3 |
| Numbers analysed | Number of participants analysed in each group | Abstract paragraph 3 |
| Outcome | For the primary outcome, a result for each group and the estimated effect size and its precision | Abstract paragraph 3 |
| Harms | Important adverse events or side effects | Abstract paragraph 3 |
| Conclusions | General interpretation of the results | Abstract paragraph 4 |
| Trial registration | Registration number and name of trial register | Abstract paragraph 5 |
| Funding | Source of funding | Title page last sentence |
