# Supplementary material for: Effect of midwife-led pelvic floor muscle training on prolapse symptoms and quality of life in women with pelvic organ prolapse in Ethiopia: A Cluster-randomized controlled trial
Source: PLoS Med. 2025 Mar 31;22(3):e1004468. doi: 10.1371/journal.pmed.1004468 (PMC11977982; doi:10.1371/journal.pmed.1004468)
Supplement: S5 File — (DOCX) [file pmed.1004468.s005.docx]

Supplementary File 5: Mann-Whitney U test for cluster-level analysis of the effects of midwife-led pelvic floor muscle training on prolapse symptoms and prolapse related quality of life.

| **Outcome** | | **After 6months** | | **Mean difference** | **P-value)** |
| --- | --- | --- | --- | --- | --- |
|  |  | **Intervention (n=4)** | **Control (n=4)** |  |  |
| POP-SS (mean ±SD) | | 10.63 (± 0) | 11.62 (± 0) | 0.98 | P < 0.001 |
|  | **Prolapse-Quality of life** | | | | |
| Physical function (mean ±SD) | | 34.44 (± 0) | 38.67 (± 0) | 4.34 | P < 0.001 |
| Personal relationship (mean ±SD) | | 36.49 (± 0) | 40.2 (± 0) | 3.72 | P < 0.001 |
| Psychological domain (mean ±SD) | | 33.9 (± 0) | 38.6 (± 0) | 4.72 | P < 0.001 |

*n = number of clusters, SD= standard deviation*

*** Mean difference= Mean (control)-mean (intervention)*

**Note:**

This table presents supplementary findings from the cluster-level analysis; our primary intention was to conduct an individual-level analysis.
