# Supplementary material for: Effect of midwife-led pelvic floor muscle training on prolapse symptoms and quality of life in women with pelvic organ prolapse in Ethiopia: A Cluster-randomized controlled trial
Source: PLoS Med. 2025 Mar 31;22(3):e1004468. doi: 10.1371/journal.pmed.1004468 (PMC11977982; doi:10.1371/journal.pmed.1004468)
Supplement: Attachment — Submitted filename: PLOS Medicine D2402632 comments on revision.docx [file pmed.1004468.s008.docx]

**PMEDICINE-D-24-02632**

I still have some residual concerns as follows:

- Table 1 – numbers in the clusters don’t add up to the number of participants in the 2 districts in the rows above
- Reference 34 relates to the PROPEL study not the POPPY trial – line 272
- Don’t really understand why they translated the POP-SS from English to Sidaamu Afoo when an Amharic version exists and they translated the P-Qol from Amharic to Sidaamu Afoo. The 2 different approaches are hard to justify.
- Should add “n”s to table 2 as well as mean and SD.
- I am still not sure why they used a Bonferroni correction, and why for the primary outcome measure analyses but not others.
- Re interactions and subgroups – text still not clear about whether interaction terms were included in the models to look for subgroups effects. If yes, then why the need for a stratified analysis? Text says: To control for effect modifiers, we employed statistical tests to assess interactions between PFMT and potential effect modifiers. This approach allowed us to determine whether the effect of PFMT varies across different subgroups. Additionally, we conducted stratified analyses to examine outcomes within specific strata, such as age and severity of prolapse.
