# Supplementary material for: Effect of midwife-led pelvic floor muscle training on prolapse symptoms and quality of life in women with pelvic organ prolapse in Ethiopia: A Cluster-randomized controlled trial
Source: PLoS Med. 2025 Mar 31;22(3):e1004468. doi: 10.1371/journal.pmed.1004468 (PMC11977982; doi:10.1371/journal.pmed.1004468)
Supplement: Attachment — Submitted filename: A Response Letter to Reviewers 3rd round.docx [file pmed.1004468.s011.docx]

**A point-by-Point Response for Reviewers 3^rd^ Round**

Title: Effect of Midwife-Led Pelvic Floor Muscle Training on Prolapse Symptoms and Quality of Life in Women with Pelvic Organ Prolapse in Ethiopia: A Cluster-Randomized Controlled Trial

Dear Editor,

We sincerely appreciate PLOS Medicine for the opportunity to receive expert feedback and enhance our manuscript. We are especially grateful for your readiness to accept our work, and we extend our heartfelt thanks to you and the diligent reviewers who dedicated their time to assess our research. For a point-by-point response and reviewer comments, please see the table below.

| **Editors and Reviewer’s Comments** | **Author’s Response** | **Amendment section** |
| --- | --- | --- |
| **Requests from Editors:** |  |  |
| GENERAL EDITORIAL REQUESTS * At this stage, we ask that you include a short, non-technical Author Summary of your research to make findings accessible to a wide audience that includes both scientists and non-scientists. The Author Summary should immediately follow the Abstract in your revised manuscript. This text is subject to editorial change and should be distinct from the scientific abstract. Ideally each sub-heading should contain 2-3 single sentence, concise bullet points containing the most salient points from your study. In the final bullet point of ‘What Do These Findings Mean?’ Please include the main limitations of the study in non-technical language. | Thank you very much for your feedback regarding the inclusion of a non-technical Author Summary.  We appreciate the importance of making our findings accessible to a wider audience.  We would like to clarify that this section has already been included in the manuscript. The Author Summary follows the Abstract and is structured with clear sub-headings, containing concise bullet points that highlight the key aspects of our study. | Page 4 |
| * Please confirm that your title complies with to PLOS Medicine's style. Your title must be nondeclarative and not a question. It should begin with main concept if possible. "Effect of" should be used only if causality can be inferred, i.e., for an RCT. Please place the study design ("A randomized controlled trial," "A retrospective study," "A modelling study," etc.) in the subtitle (ie, after a colon). | Thank you once again for your feedback.  We would like to confirm that our title is neither declarative nor a question. The term “Effect” has been included because our study design is a cluster-randomized trial, which allows for causal inference. We have indicated this design in the title following a colon | Line 1  Line 2 |
| * Please confirm that your abstract complies with our requirements, including providing all the information relevant to this study type | Thank you for your feedback.  We have carefully reviewed our abstract and confirm that it complies with PLOS Medicine’s style guidelines, specifically following the CONSORT criteria for abstract | Page 2 |
| * Please ensure that the Introduction ends with a clear description of the study question or hypothesis. | Thank you for your feedback.  We have ensured that the Introduction concludes with a clear hypothesis, which highlights that the intervention will improve symptoms. This statement effectively sets the stage for the subsequent sections of the manuscript | Line 137-139 |
| * Please ensure that all abbreviations are defined at first use throughout the text. | Thank you for your feedback.  We rechecked the manuscript and found that all abbreviations have been defined at first use throughout the text. This ensures clarity and accessibility for all readers. |  |
| **GENERAL** |  |  |
| FUNDING STATEMENT * The funding statement should include: specific grant numbers, initials of authors who received each award, URLs to sponsors’ websites. Also, please state whether any sponsors or funders (other than the named authors) played any role in study design, data collection and analysis, the decision to publish, or preparation of the manuscript. If they had no role in the research, include this sentence: “The funders had no role in study design, data collection and analysis, decision to publish, or preparation of the manuscript.” | Thank you for your feedback.  We have included the URLs to the sponsors’ websites, specified their roles, and identified the author who received the support. This support was specifically allocated for the per diem of data collectors and supervisors. However, we would like to note that there is no specific grant number assigned to this support. | Line 649-53 |
| COMPETING INTERESTS STATEMENT * All authors must declare their relevant competing interests per the PLOS policy | Thank you  All authors declare that they have no competing of interest | Line 656 |
| DATA AVAILABILITY * It appears that you have included data that may breach patient confidentiality including phone numbers and significant dates for the participants. Please edit your data set in accordance with patient consent, and remove any identifying data. | Thank you for your feedback.  We have removed the phone numbers, names of data collectors, and significant dates from the dataset to ensure compliance with patient confidentiality and consent requirements. | Supplementary file 2 |
| **Reviewer #1:** |  |  |
| Thank you for the updates. My only outstanding comment relates to the cluster-level analysis that I requested. I could see from the ChatGPT output that you shared that you were not quite sure about how to go about this. The best approach is to keep it simple. You were correct to use the collapse command to get cluster level summaries for each outcome. The next step in the analysis just involves comparing these results (i.e. a 4 versus 4 data point comparison) using a t-test. There is no need to adjust for other covariates (doing so actually messes up the cluster-level comparison) or to allow for the survey design for example. So you could just generate the cluster-level means and then use a command such as "ttest outcome, by(trialarm)" to do the analysis. I had a quick look at doing this using the figures that you now include in table 2 (which was a very helpful addition). I found that there was still evidence for each of these outcomes of a difference between trial arms, which was reassuring.  Therefore, my only outstanding suggestion is to update the new supplementary table 5 using results from the commands suggested above. Related to this table, I also suggest removing the 4 bullet points underneath, as the cluster-level analysis is widely considered to be a robust approach (more robust than individual level analysis when there are only 8 clusters) - there are many counter-criticisms of individual level analysis methods for 8 clusters that you do not mention (and some of the bullet points included, e.g. number 3, are not really relevant as you are still finding evidence of a difference between trial arms, so clearly lack of power isn't a problem). Ultimately, it is reassuring that both approaches give consistent results. | Thank you very much for your feedback and support regarding the cluster-level analysis.  We have included the results of the Mann-Whitney U test in Supplementary Table 5. This version presents the means, mean differences, and significance levels for the outcomes. We chose the Mann-Whitney U test over the t-test because the collapsed data violates the assumptions required for a t-test.  We appreciate your guidance on this analysis and look forward to any further suggestions you may have. | Supplementary file 5 |
