# Supplementary material for: Effect of midwife-led pelvic floor muscle training on prolapse symptoms and quality of life in women with pelvic organ prolapse in Ethiopia: A Cluster-randomized controlled trial
Source: PLoS Med. 2025 Mar 31;22(3):e1004468. doi: 10.1371/journal.pmed.1004468 (PMC11977982; doi:10.1371/journal.pmed.1004468)
Supplement: Attachment — Submitted filename: A Response Letter to Reviewers.docx [file pmed.1004468.s009.docx]

**A point-by-Point Response for Reviewers**

Title: Effect of Midwife-Led Pelvic Floor Muscle Training on Prolapse Symptoms and Quality of Life in Women with Pelvic Organ Prolapse in Ethiopia: A Cluster-Randomized Controlled Trial

Dear Editor,

We sincerely appreciate the Journal of PloS Medicine for granting us the opportunity to receive expert feedback and enhance our manuscript. Our heartfelt thanks go to the editor and the diligent reviewers who dedicated their time to assess our work. For a point-by-point response and reviewer comments, please see the table below.

| **Reviewer Comments** | **Author Responses** | **Amendment** |
| --- | --- | --- |
| **Reviewer#1** |  |  |
| 1.      The authors state that the size of the kebeles (in terms of population) can be very variable. Was this taken into account at all in the randomisation/selection of clusters procedures? i.e. how did they ensure that there were roughly equal numbers in each trial arm? Perhaps they could include information on the size of the selected kebeles in the manuscript? More generally, it would be helpful to include cluster-level data in table 1, in addition to the individual-level data. | Thank you very much.  In response to your suggestion, we have incorporated summary of cluster-level data into Table 1. We used cluster randomization simply **to minimize spillover of the intervention to the control arm (i.e. we were not concerned about more cluster-level variables).**  Regarding sample selection process, for this study, we expected roughly equal numbers in each trial arm because we screened equal numbers (102) of participants from each cluster.  To explain how equal number of participants were screened for the baseline study, we descried it in the previous baseline study **[1]**. To account for this unequal probability of selection, we used a complex survey data analysis that account for unequal probability of selection, cluster effect and stratification effect at analysis stage but not at design effect [2]. Complex survey analysis is a statistical approach specifically designed to handle data collected from surveys with complex sampling designs. Moreover, in this study, we included all eligible cases for the reasons we indicated in the sample size determination. | Table 1 |
| 2.      The intervention was implemented at health centres. I found the sentence "Each cluster in the study area is served by its own health posts, staffed by health extension workers, while five or more clusters are supported by a single health center." unclear. Does that mean that a group of 5 of the kebeles included in the study all were covered by the same health centre? If so, then it seems this could lead to issues of contamination, i.e. if women from the intervention arm and the control arm are all receiving care at the same health centre, how did the study team ensure that each woman received the allocated care - how were they identified and how did they ensure there was no contact between women receiving the intervention and those who didn't when they were visiting the health centres? But I think I may be misunderstanding what is meant by "five or more clusters are supported by a single health centre". | Thank you again.  We appreciate your insights and have made revisions to clarify this important point and indicated that these eight clusters are from eight different health centers. In Ethiopia, each kebele is served by its own health post and one health center will serve five kebeles (i.e. it is the structure of a health system) in general. | Line 227 |
| 3.      The authors state "At three months and six months of intervention, four first degree nurses went to the house of the study participants and collected the data through face-to-face interviews. The data collection tool included the POP-SS and P-QoL questionnaires used at baseline, PGI-I change and questions used to assess their adherence to training protocol".  How could this last point (adherence to training protocol) be done while keeping the assessors masked to the intervention allocation for each woman, as was earlier stated to be the case in the "masking" section of the methods? | Thank you for your insightful questions.  We assigned different data collectors for the intervention and control sites, with two data collectors dedicated to each arm. This separation was intended to minimize any potential bias or contamination during data collection.  We acknowledge your concern that there is possibility of communication between data collectors, as they may know each other. We recognize that this could pose a limitation to our study. Although the risk of contamination is minimal due to the distinct assignment of data collectors, we did not fully account for this potential issue in our initial design. |  |
| 4.      The POP-Q measure was used to assess eligibility for inclusion in the study. Did the authors consider assessing POP-Q again at 6 months to provide a more objective endpoint? | Thank you again  We did not perform the POP-Q assessment at the six-month follow-up due to budget limitations. The costs associated with this procedure included hiring experts to do the POP-Q, transportation for both the assessors and study participants, and compensation for participants' time. Given these financial constraints, we prioritized the initial assessments and other essential components of the study.  We acknowledge that conducting a follow-up POP-Q assessment at six months would have provided valuable objective data regarding changes in pelvic organ prolapse status over time. This additional information could have strengthened our findings and offered deeper insights into the intervention's effectiveness |  |
| 5.      In the statistical methods section the authors state that "All data analyses accounted for the complex sampling design, which included stratification by district (Dale and Wonsho), clustering by kebele, and weighting for unequal probability of selection. The weighting variable was the product of cluster and household selection weights…" As far as I can see this is the first mention of any household selection, please could this be clarified? | Thank you again  We briefly introduced this description that we selected households randomly from the selected clusters (for the baseline data), and all eligible women were recruited for this study). The detailed methodology regarding household selection and the overall sampling design was described in our previously published baseline data **[1].** | Line 199-207 |
| 6.      The authors use an individual-level analysis, while accounting for the clustered design. However, this approach could be unreliable with a small number of clusters (as there are in this study). For reassurance, the authors should include an additional analysis which allows for the small number of clusters, for example the cluster-level analysis described in the Hayes and Moulton textbook referenced. It seems that the ICC is fairly low (and indeed, the authors find that there is no improvement to the model fit by allowing for the clustering) so this may be overkill, but it would be reassuring if the same or similar results were found. | Thank you for your insightful feedback regarding our statistical methods.  We have now included a two-stage analysis as recommended by Hayes and Moulton [3]. In the first stage, we summarized key outcome measures, the mean change in POP-SS and quality of life (QoL) scores for each cluster (Table 2). In the second stage, we conducted comparisons between the intervention and control arms. Table 3 presents the mean change differences without adjusting for other factors, while Table 4 provides the adjusted results, accounting for covariates.  When the clustering variable is retained in the model, there is some decimal change to coefficients (effect size) and confidence interval with some deterioration to the value of Pseudo Log-Likely hood. | Table 2-4 |
| 7. In the statistical methods the authors state "The model included fixed effects for treatment group, time, and other relevant covariates." What are the other relevant covariates and how was their relevance determined? | Thank you for your question.  In our analysis, we considered the following covariates: stage of prolapse, site of prolapse, duration of prolapse symptoms, parity, age, level of education, history of prolonged labor, constipation, and chronic cough.  The relevance of these covariates was determined based on prior research, clinical aspect, and their potential impact on the prolapse symptoms and quality of life.  Our selection process for covariates involved several systematic steps [4]:  We first examined whether the covariates were significantly different between the intervention and control arms. This step ensured that any baseline differences were accounted for in our analysis.  Next, we identified covariates that were associated with the outcome variable, the Pelvic Organ Prolapse Symptom Score (POP-SS) and each component of quality of life. This was done through preliminary analyses to assess their relationships with the outcome.  Each significant covariate was then fitted to the model using a stepwise approach.  Finally, we retained variables that demonstrated significance through the Wald test and improved overall model fit. This ensured that only relevant covariates were included in the final multilevel mixed-effects generalized linear model (MEGLM). |  |
| 8.      The authors describe a rather complicated strategy of analysis approach based on determining the best fit of models containing possible confounders and effect modifiers. I would question whether they need to do this since this is a randomised trial. Were there any potential confounders that were imbalanced in the randomisation and/or that were highly predictive of the outcome(s)? Were the possible effect modifiers pre-specified? Relating to effect modification, please include explanation of how effect modification was tested and provide the results (rather than just saying that there were no significant interactions). | Thank you for your thoughtful questions  This study was conducted as a cluster randomized controlled trial (RCT). To enhance the robustness of our findings, we employed stratified cluster sampling based on geographical location, which aimed to balance characteristics across both arms of the trial. However, we acknowledge that this method does not guarantee perfect balance of individual-level covariates and there were variables that affected the effect of the intervention. We pre-specified potential confounders in the protocol to ensure transparency and rigor in our analysis.  Regarding effect modifiers, we used an interaction term to assess whether the effect of PFMT differs by the third variable. However, since we didn’t get significant interaction and even presence of such variables distort the model fitness, we removed them from the model based on the recommendation given by Hosmer and colleagues [4]. Moreover, to avoid confusions, we removed the sentence that state “there were no significant interactions” from the revised manuscript. |  |
| 9.      "All prolapse symptoms were significantly improved from the baseline for the intervention group but in the control group, "a feeling of vaginal discomfort/pain and something passing through vagina remained persistent"." This wording is not very clear, as all the symptoms remained persistent in both intervention and control groups, although the proportion was reduced in the intervention group. The wording could be improved. | Thank you again  Based on the issues raised by reviewer #3, we have removed that section to maintain reader’s focus on the between-group comparisons rather than pre-post comparisons. |  |
| 10.     There are some sizeable differences in some characteristics between trial arms, for example age, parity. What happens when these are controlled for in the analysis? The sentence "Overall, the intervention and control arms were balanced in terms of most characteristics at baseline" should be modified to reflect that there are some characteristics (which may be important) which are very imbalanced. | Thank you for your insightful comments  We acknowledge that there are notable differences in certain characteristics, such as age and parity, and family history of POP between the intervention and control groups. We controlled for these variables in our analysis and found no significant effect on the outcomes. However, in the multivariable model, we retained only variables that are found to be important based on Wald-test and model fitness. It is recommended not to retain variables that are neither significant nor improve model fitness [4].  In light of your feedback, we have revised the statement to more accurately reflect the situation: "Overall, while the intervention and control arms were balanced in terms of most characteristics at baseline, there were some important characteristics that showed significant imbalances ". | Table 1  Line 414-416 |
| 11.     The Table 3 title does not accurately reflect what is shown in Table 3. The title suggests that this table will show effect modifiers in some way, but what is actually shown is fixed effect models (i.e. no interaction terms). The title should be changed to reflect what is actually shown. Also in Table 3, it would be good to be clear that it is POP-SS score at baseline that is shown as a covariate in the model results for the quality-of-life outcomes. | Thank you for your constructive feedback  We have changed the title to “Adjusted Effect of Pelvic Floor Muscle Training, Prolapse Stage, and Other Predictors on Prolapse Symptoms and Quality of Life in Sidama Region, Ethiopia, August 2024” to indicate what is actually shown.  Furthermore, we have clarified that the POP-SS score presented as a covariate is the baseline result. | Table 4 |
| 12.     The interpretation in the discussion "The effects of PFMT were more noticeable in women with prolapse stage-I with a mean change difference of -5.08 and stage II with a mean change difference of -3.06 as compared to stage III in reducing POP-SS." is not correct. The way this sentence is worded implies that it is the effect of the intervention that differs by stage (i.e. effect modification) but actually what these figures represent are the effect of stage on the improvement (i.e. regardless of trial arm, since that is controlled for as a fixed effect in the model). This needs to be corrected. The sentence "In the present study, women with stage I and II prolapses reported more noticeable improvements compared to those with stage III prolapses indicating that the earlier stage of prolapse are more responsive to PFMT. " Suffers from the same issue and should also be re-worded. | Thank you for your important feedback.  We revised the interpretation to show that the stage has its own effect on POP-SS as a covariate. We stated that “The study indicates that while women with Stage I and II prolapse experienced more pronounced improvements in symptoms and quality of life after PFMT, women with Stage III also benefited from the intervention. This suggest that PFMT can still provide symptom relief and enhance quality of life for women at this stage”. | Line 498 and 534-536 |
| 13.     One potential limitation that should be mentioned in the discussion is that the follow-up is fairly short and so we cannot tell whether the effects are likely to be sustained. This could be commented on. | Thank you for your insightful suggestion.  We have acknowledged this potential limitation in the discussion section of the manuscript. | Line 573 |
| **Minor comments** |  |  |
| 1.      In the abstract, results, the authors should specify the number of clusters randomized to each arm (since this is the unit of randomization) as well as the number of women who are in each arm. | Thank you for your valuable feedback.  We have indicated that 4 clusters were assigned to each arm with 101 women to the control arm and 86 to the intervention arm. | Line 41-42 |
| 2.      "Our hypothesis was that providing midwife-led PFMT monthly followed by three times per-day home-based exercise by participants for six months" Should this be "Our hypothesis was that providing midwife-led PFMT monthly alongside three times per-day home-based exercise by participants for six months". Also the midwife-led PFMT is approximately monthly as the first appointments are spaced more closely together. | Thank you for your insightful suggestion.  We have revised the statement to read: "Our hypothesis was that providing midwife-led PFMT approximately monthly alongside three times per-day home-based exercise by participants for six months significantly reduce POP-SS and improve prolapse quality of life”. | Line 130-132 |
| 3.      In Table 2, difficulty to empty bowel appears twice, with different data. | Thank you for your feedback.  We have corrected that the first one was “difficulty to empty bladder “. | Table 1 and 3 |
| 4.      There is some inconsistency in numbers of decimal places/significant figures presented in tables - it would be good to make everything consistent. | Thank you for your observation.  We have corrected this issue and ensured that all decimals were adjusted to two digits. | Table 1-4 |
| **Reviewer#2** |  |  |
| I have read the first 33 pages of the PDF. It seems that there is a copy in the second half of the document. | Thank you for your comment.  This manuscript has only 27 pages followed by 8 pages of references. We are unsure what happened to the version you received. |  |
| This is a very valuable paper as there is only limited information on prolapse treatment low resource countries. Creative manner of blinding that may work well for the purpose of this study. Is there any additional information on the social interaction between the clusters? And this alternative blinding may be listed in the limitations. | Thank you for your comments.  Women from one cluster may interact with those in other clusters during events like funerals or through shared relatives. However, given the sensitivity of prolapse, we believe that such discussions are unlikely to occur during these occasions. Furthermore, we intentionally established a buffer zone to ensure that the clusters are sufficiently distanced from one another. |  |
| Is 'an abundance of midwifes' the correct wording? (There are still major problems with reaching all women in Ethiopia for midwifery care, as far as I know. But maybe not due to numbers of midwifes but only poor spread over the country?) | Thank you for your feedback.  We have rephrased that section to clarify that midwives are generally more accessible than specialists, rather than implying an abundance of midwives overall |  |
| POP-Q may need a reference. | Thank you again  It has been cited reference #25 | Line 149 |
| Page 8: typo in 'participants of for intervention' | Thank you very much  it has been corrected as “'..participants for intervention”. | Line 215 |
| Lifestyle intervention might be better explained. This is also an interesting cohort for the effect of lifestyle modification in the control group, but to be useful this needs more explanation and discussion then. | Thank you  We have now included a brief explanation of the lifestyle intervention. | Line 280-305 |
| There are 4 conditions mentioned but not e.g. what would have been the measures taken in case of constipation, chronic cough etc. or was it only information given, and if yes what type of information. | Thank you for your inquiry. The basic counseling on lifestyle modifications was designed to address and prevent conditions such as constipation and chronic cough. In cases where our counseling did not yield results (including prolapse stage 4), we advised them to seek further treatment. Additionally, after noticing that the control group was not benefiting equally from the intervention, we collaborated with training health centers and Hamlin Fistula centers to facilitate appropriate referrals for those in need of additional care. | Line 280-305 and declaration section paragraph 1 |
| Is it known whether the women completed the questionnaires by themselves in their house (fully self-reported)? Were there analphabets included? Or was this done through these face-to-face interviews? We observers/nurses instructed explicitly not to influence the answers? | Thank you for your questions.  The interviews were conducted entirely through face-to-face interactions as many participants were uneducated. In this study, "self-report" means that participants reported their symptoms rather than completing the questionnaires independently. To minimize interviewer bias, we trained the interviewers to remain non-judgmental and provided timely supervision. Furthermore, they were blinded to the allocation groups to reduce the risk of interviewer bias. | Line 334-42 |
| This is what is mentioned: 'At baseline, a slightly higher number of women in menopause, family history of prolapse, and history of home birth were observed in the intervention group.' But how do you explain the difference in Age at base line between groups which is unexpected in an RCT? The median in the control group is lower than the lower bound of interquartile range in intervention group. | Thank you for your question.  During randomization, we aimed to balance the two groups by allocating similar clusters using stratified cluster sampling. However, this method cannot account for individual-level variables, which is an inherent limitation of cluster sampling. Regarding the age discrepancy, it may stem from inaccuracies in measuring age, as many individuals in rural Ethiopia has no birth certificates. We indicated this point as a limitation of the current study | Line 574-80 |
| Why was age not considered as effect modifier? | Thank you for your question.  We did examine age as a potential effect modifier but it was not significant. Its presence in the model distorted the model fitness so that we removed it from the final model [4]. This could be due to in accurate age estimation. We have detailed this issue in the previously published baseline data of this manuscript [1] |  |
| Cloud you try to explain that 26% of women in the control group became worse in 6 months? | Thank you  We clearly stated it in the revised version indicating that 26% of women in the control group became worse in 6 months. | Line 461 |
| **Reviewer #3:** |  |  |
| Re: "The detail of the counselling was provided using a Sidaamu 249 Afoo (local language) version of a the PROPEL realist evaluation guideline [33]", I am not sure what this means as the PROPEL study was an implementation study relating to a previous trial - POPPY. Did the authors use the Lifestyle Advice Leaflet from the POPPY trial? | Thank you for your question.  We have clarified this in our revised manuscript. We translated the Lifestyle Advice Leaflet from the POPPY trial into Sidaamu Afoo, the local language, and used it for counseling purposes. | Line 269-71 |
| I wondered by the Amharic version of the POP-SS was not used (Belayneh, T., Gebeyehu, A., Adefris, M. et al. Validation of the Amharic version of the Pelvic Organ Prolapse Symptom Score (POP-SS). Int Urogynecol J 30, 149-156 (2019). <https://doi.org/10.1007/s00192-018-3825-x>) since the Amharic version of the P-QoL was used. | Thank you for your question.  While Amharic is the official language of Ethiopia, there are communities, particularly in rural areas of the southern part of the country that speak their own languages and do not understand Amharic. To accurately assess pelvic organ prolapse symptoms in these communities, we translated the POP-SS into Sidaamu Afoo, the local language, and validated its psychometric properties (validity and reliability).  However, we did not conduct a formal validation for the P-QoL questionnaire. Instead, we translated it from Amharic to Sidaamu Afoo without testing its psychometric properties, as we assumed that the Amharic version would be culturally relevant to our study area than the English version. |  |
| Sample size calculated based on 1% significance level to account for multiple comparisons, however many of the comparisons made were unnecessary. So perhaps 5% level of significance was appropriate. It is not good stats practice to do statistical tests on baseline variables (Table 1). Also I question why do univariate tests (Table 2) when the primary analysis is a multivariable model? | Thank you for your comments. We appreciate your feedback  We lately acknowledged that performing statistical tests on baseline variables may not be the best practice, particularly in the context of cluster-randomized trials (CRTs). In light of this, we revised Table 1 to focus solely on descriptive statistics.  In this study, the significance level was adjusted only for the primary outcomes (POP-SS and three domains of QoL). While we conducted additional comparisons for secondary outcomes, we did not adjust the significance level for these. For the primary outcomes, we followed the Bonferroni correction to account for multiple comparisons, as we were concerned that failing to do so could lead to a Type I error [5].  Regarding univariate analysis, we recognize that usually it may not be necessary to do univariate analysis if the primary focus of the study is on multivariable models. However, when the number of cluster is limited, it is recommended to use a two-stage analysis that first present a summary results per each arm and followed by a comparison across different arms [3]. Moreover, univariate analysis can provide insights into individual variables. Therefore, we prefer to maintain both tables. |  |
| Paragraph from line 392-396: does this relate to Table 2? - add (Table 2) to indicate this. Says "All prolapse symptoms were significantly improved from the baseline for the intervention group but in the control group, "a feeling of vaginal discomfort/pain" and "something passing through vagina" remained persistent" - (extra quotation marks needed) but the reader cannot tell this from Table 2 as there are no pre-posttests presented. In any case I think the pre-post testing is a distraction from the main analysis which is a between groups analysis, and adds to the number of comparisons done. | Thank you for your feedback. We appreciate your insight on this matter  We have removed that section from the manuscript. As you pointed out, it detracted from the main analysis, which focuses on between-group comparisons. By omitting this content, we aim to maintain clarity and ensure that readers can easily follow the primary analysis without being distracted by pre-post testing results. |  |
| **Modelling methods** Were other baseline variables (eg. age) included in the models? Why are there different moderator variables for different outcomes? What was the process for fitting/retaining/excluding variables from models? | Thank you for your questions.  In our models, we included selected baseline variables that improved model performance, were significant based on the Wald test, and were supported by existing literature.  The relevance of these covariates was determined through a combination of prior research, clinical expertise, and their potential impact on the outcome variable. Our selection process for covariates involved several systematic steps:   1. **Initial Comparison**: We first examined whether covariates were significantly different between the intervention and control groups to account for any baseline differences in our analysis. 2. **Association with Outcome Variable**: Next, we identified covariates associated with the outcome variable, the Pelvic Organ Prolapse Symptom Score (POP-SS), through preliminary analyses assessing their relationships with the outcome. 3. **Stepwise Model Fitting**: Each significant covariate was fitted to the model using a stepwise approach, allowing us to evaluate the contribution of each variable incrementally. 4. **Wald Test and Model Fit**: Finally, we retained variables that demonstrated significance through the Wald test and improved overall model fit, ensuring that only relevant covariates were included in the final multilevel mixed-effects generalized linear model (MEGLM).   Specific to age variable, we included it in the model but it was not significant but affected model fitness. As explained in our response to Reviewer #2, many participants in our study lacked birth certificates, making it difficult to obtain accurate age information. We have discussed this issue in detail in the previously published baseline data for this study **[1]**. |  |
| In the POP-SS model in Table 3 symptom duration and prolonged labour are included although not significant.  Baseline variables are not balanced eg. age, family history. This is not really discussed. It is more important then to include baseline variables in the models to adjust for this | Thank you for your feedback.  When selecting variables to retain in the model, we focused on those deemed important based on the Wald test and those that improved model fit. In the POP-SS model presented in Table 3, while symptom duration and prolonged labor were not significantly associated with the outcome, their inclusion was justified as they contributed to a better overall model fit according to the Wald test.  We acknowledge that baseline variables such as age and family history were not balanced between groups. We discussed this issue in the revised manuscript. | Line 574-80 |
| Line 337: "The random intercept was insignificant and the model without the cluster variable provided a better fit". I query whether this variable should actually be retained in the model regardless as it correctly reflects the cluster trial design. | Thank you for your valuable feedback.  We have now included the random intercept in the model to accurately reflect the cluster trial design, regardless of its significance. |  |
| Paragraph beginning line 400: were interaction terms fitted? This was not mentioned in the methods. If no interactions terms with intervention were included then there was no subgroup analysis. Therefore the statements about the intervention effect being different for different subgroups, eg. stage I and II prolapse, are incorrect. For example the following is not accurate: "The effects of PFMT were more noticeable in women with prolapse stage-I with a mean change difference of -5.08 and stage II with a mean change difference of -3.06 as compared to stage III in reducing POP-SS." All women with stage I and II had a greater improvement in POP-SS than stage III women (once the effect of the intervention had been accounted for) not just the women in the PFMT arm. In order to test that an interaction term (Stage*Intervention) would need to be included in the model. | Thank you for your important feedback regarding the interpretation of our findings.  Now we corrected the way it was interpreted. We stated that “while women with Stage I and II prolapse experienced more pronounced improvements in symptoms and quality of life after PFMT, women with Stage III also benefited from the intervention. This suggest that PFMT can still provide symptom relief and enhance quality of life for women at this stage”.  We tested for the interaction but it was not significant | Line 534-36 |
| Was it baseline POP-SS that was included in the P-QoL models (Table 3). What it the rationale for including an outcome as a predictor variable? | Thank you  We included the baseline POP-SS in to the model to control for individual differences in symptom severity. Now we clearly stated that variable as a baseline POP-SS | Table 4 |
| Wording Typo in Table 1 and 2 - "Difficulty to empty bowel" appears in 2 lines. One should be "a feeling that your bowel has not emptied completely". | Thank you very much.  We corrected it now, the first one was to ask about difficulty to empty bladder, not a bowel. | Table 1 and 3 |
| Line 330: "along with the outcome variables" - the intervention variable is fitted to the outcome variable not along with | Thank you for your feedback  We have revised it to clarify that the intervention variable is fitted to the outcome variable. | Line 382 |
| **General editorial requests: (Note: not all will apply to your paper, but please check each item carefully)** | | |
| We ask every co-author listed on the manuscript to fill in a contributing author statement, making sure to declare all competing interests…… | Thank you  We had already done it |  |
| Please upload any figures associated with your paper as individual TIF or EPS files with 300dpi resolution at resubmission; please read our figure guidelines for more information on our requirements: <http://journals.plos.org/plosmedicine/s/figures>. While revising your submission, please upload your figure files to the PACE digital diagnostic tool, <https://pacev2.apexcovantage.com/>. PACE helps ensure that figures meet PLOS requirements. To use PACE, you must first register as a user. Then, login and navigate to the UPLOAD tab, where you will find detailed instructions on how to use the tool. If you encounter any issues or have any questions when using PACE, please email us at [PLOSMedicine@plos.org](mailto:PLOSMedicine@plos.org) | Thank you  We did it accordingly | Figure 1 |
| Please ensure that the paper adheres to the PLOS Data Availability Policy (see <http://journals.plos.org/plosmedicine/s/data-availability>), which requires that all data underlying the study's findings be provided in a repository or as Supporting Information. For data residing with a third party, authors are required to provide instructions with contact information (web or email address) for obtaining the data. Please note that a study author cannot be the contact person for the data. PLOS journals do not allow statements supported by "data not shown" or "unpublished results." For such statements, authors must provide supporting data or cite public sources that include it. | Thank you  We provided the Stata data file as Supplementary file 2 |  |
| *       We expect all researchers with submissions to PLOS in which author-generated code underpins the findings in the manuscript to make all author-generated code available without restrictions upon publication of the work. In cases where code is central to the manuscript, we may require the code to be made available as a condition of publication. Authors are responsible for ensuring that the code is reusable and well documented. Please make any custom code available, either as part of your data deposition or as a supplementary file. Please add a sentence to your data availability statement regarding any code used in the study, e.g. "The code used in the analysis is available from Github [URL] and archived in Zenodo [DOI link]" Please review our guidelines at <https://journals.plos.org/plosmedicine/s/materials-software-and-code-sharing> and ensure that your code is shared in a way that follows best practice and facilitates reproducibility and reuse. Because Github depositions can be readily changed or deleted, we encourage you to make a permanent DOI'd copy (e.g. in Zenodo) and provide the URL. | Thank you  We have no personal codes to declare |  |
| FORMATTING - GENERAL *       Abstract: Please structure your abstract using the PLOS Medicine headings (Background, Methods and Findings, Conclusions). Please combine the Methods and Findings sections into one section. | Thank you  We restructured it | Abstract |
| At this stage, we ask that you include a short, non-technical Author Summary of your research to make findings accessible to a wide audience that includes both scientists and non-scientists. The Author Summary should immediately follow the Abstract in your revised manuscript. This text is subject to editorial change and should be distinct from the scientific abstract. Ideally each sub-heading should contain 2-3 single sentence, concise bullet points containing the most salient points from your study. In the final bullet point of 'What Do These Findings Mean?', please include the main limitations of the study in non-technical language. Please see our author guidelines for more information: <https://journals.plos.org/plosmedicine/s/revising-your-manuscript#loc-author-summary> | Thank you  We provide the Author summary next to the abstract section |  |
| Please express the main results with 95% CIs as well as p values. When reporting p values please report as p < 0.001 and where higher as the exact p value p=0.002, for example. Throughout, suggest reporting statistical information as follows to improve clarity for the reader "22% (95% CI [13%,28%]; p</=)". Please be sure to define all numerical values at first use. | Thank you  We did it with 99% CI because we adjusted the significance level for multiple comparison since we are measuring four outcomes from a single study. Failure to adjust this may introduce type I error [5]. |  |
| Please include page numbers and line numbers in the manuscript file. Use continuous line numbers (do not restart the numbering on each page). | Thank you  We did it |  |
| Please cite the reference numbers in square brackets. Citations should precede punctuation. | Thank you  We did it |  |
| FIGURES AND TABLES *       Please provide titles and legends for all figures and tables (including those in Supporting Information files). | Thank you  We did it |  |
| Please define all abbreviations used in each figure/table (including those in Supporting Information files). | Thank you  We did it |  |
| Please consider avoiding the use of red and green in order to make your figure more accessible to those with color blindness. | Thank you  We have no such colors in one Figure 1. |  |
| SUPPLEMENTARY MATERIAL *       Please note that supplementary material will be posted as supplied by the authors. Therefore, please amend it according to the relevant comments outlined here.  *       Please cite your Supporting Information as outlined here: <https://journals.plos.org/plosmedicine/s/supporting-information> | Thank you  We did it |  |
| REFERENCES *       PLOS uses the numbered citation (citation-sequence) method and first six authors, et al. | Thank you  We did it |  |
| Please ensure that journal name abbreviations match those found in the National Center for Biotechnology Information (NCBI) databases (<http://www.ncbi.nlm.nih.gov/nlmcatalog/journals>), and are appropriately formatted and capitalised. | Thank you  We checked it |  |
| Where website addresses are cited, please include the complete URL and specify the date of access (e.g. [accessed: 12/06/2023]). | Thank you  We checked for it |  |
| Please also see <https://journals.plos.org/plosmedicine/s/submission-guidelines#loc-references> for further details on reference formatting. | Thank you  We did it |  |
| RCTs *       PLOS Medicine requires that all trials be prospectively registered in one of registries recognized by WHO. Please ensure that study registration details are included in the Methods section. | Thank you  We indicated that the trial was registered at the Pan African Clinical Trial Registry ([https://pactr.samrc.ac.za](https://pactr.samrc.ac.za/)) database, with the registration number PACTR202302505126575 |  |
| *       Please structure the Methods section using the following sub-headings: Study design and participants, Randomization and masking, Procedures, Outcomes, Statistical analysis. | Thank you  We have restructured it |  |
| Exclusion criteria not detailed in manuscript (from protocol: Women who were diagnosed to have symptomatic Pelvic Organ Prolapse stage I-III but planned to leave the study area within six months (study period), severely ill women, women with psychiatry disorders, women with delivered myoma, or women who want to have prolapse surgery within six months will be excluded.) Please clarify and explain all discrepancies between the paper and protocol. If the outcomes were not prespecified in the protocol, please define them in the Methods (Outcomes section) as post hoc and explain why they were added. Post-hoc comparisons should be presented as hypothesis generating rather than conclusive. | Thank you  Now we detailed the exclusion criteria. However, we did not get women with these criteria practically, that was why we didn’t listed these previously.  The outcomes were prespecified in the protocol under subsection “Measurement” for POP-SS and P-QoL separately (the plan was to produce two different manuscripts but now we merged it). | Line 186-89 |
| *       Please ensure that all prespecified outcomes (primary, secondary, and exploratory) are listed in the Methods/Outcomes section and indicate whether there are outcomes that are not presented in the current report. | Thank you  We presented all the prespecified outcomes. |  |
| Please specify the dates (Month Day, Year) during which study enrollment and follow up occurred. | Thank you  The enrollment period was open for eight months from 13 March to 28 October 2023. Each participant was followed for six months after enrollment. The last participants were enrolled on October 28, 2023 so the final follow-up visit was on April 27, 2024. | Line 208-10 |
| *       Please include absolute numbers wherever you report percentages; eg, n/N (%) | Thank you  We included absolute numbers for all percentage indicated. |  |
| Please present the safety data for the study including numbers of specific events and whether or not adverse events are thought to be related to treatment. AEs should be reported in the abstract, per CONSORT and CONSORT-Harms. | Thank you  We reported it in the CONSORT and main document |  |
| *       Please complete the CONSORT checklist (<https://www.equator-network.org/reporting-guidelines/consort/>) and ensure that all components of CONSORT are present in the manuscript, including how randomization was performed, allocation concealment, blinding of intervention, definition of lost to follow-up, power statement. When completing the checklist, please use section and paragraph numbers, rather than page numbers. | Thank you  We did it and attached it as a supplementary file 1 |  |
| *       Please report your abstract according to CONSORT for abstracts, following the PLOS Medicine abstract structure (Background, Methods and Findings, Conclusions) <https://www.equator-network.org/reporting-guidelines/consort-abstracts/> | Thank you  We did it as supplementary file 4 |  |
| *       If your trial had to undergo important modifications in response to extenuating circumstances, please complete the CONSERVE-CONSORT checklist and provide in your Supporting Information; (<https://www.equator-network.org/reporting-guidelines/guidelines-for-reporting-trial-protocols-and-completed-trials-modified-due-to-the-covid-19-pandemic-and-other-extenuating-circumstances-the-conserve-2021-statement/>). When completing the checklist, please use section and paragraph numbers, rather than page numbers. | Thank you  Our trial has nothing to undergo important modifications in response to extenuating circumstances. |  |
| *       In keeping with our commitment to Open Science, please include the study protocol document and analysis plan (including any amendments) as Supporting Information to be published with the manuscript if accepted. | Thank you  We uploaded it as Supplementary file 3 |  |
| *       Please note that PLOS Medicine requires prospective, public registration of a data sharing plan (as part of mandatory clinical trials registration) for all clinical trials that began enrollment on or after January 1, 2019, in accordance with ICMJE requirements. | Thank you  We indicated that the trial was registered at the Pan African Clinical Trial Registry ([https://pactr.samrc.ac.za](https://pactr.samrc.ac.za/)) database, with the registration number PACTR202302505126575.  Again we have attached the Stata data file with this manuscript as supplementary file 2. |  |

1.	Siyoum M, Nardos R, Teklesilasie W, Astatkie A. Prevalence and risk factors of pelvic organ prolapse among women in Sidama region, Ethiopia: A community-based survey. Women's Health. 2024;20:17455057241265078.

2.	Heeringa SG, West BT, Berglund PA. Applied survey data analysis: chapman and hall/CRC; 2017.

3.	Hayes RJ, Moulton LH. Cluster randomised trials: Chapman and Hall/CRC; 2017.

4.	Hosmer Jr DW, Lemeshow S, Sturdivant RX. Applied logistic regression: John Wiley & Sons; 2013.

5.	Bland JM, Altman DG. Multiple significance tests: the Bonferroni method. Bmj. 1995;310(6973):170.
