# Supplementary material for: Effect of midwife-led pelvic floor muscle training on prolapse symptoms and quality of life in women with pelvic organ prolapse in Ethiopia: A Cluster-randomized controlled trial
Source: PLoS Med. 2025 Mar 31;22(3):e1004468. doi: 10.1371/journal.pmed.1004468 (PMC11977982; doi:10.1371/journal.pmed.1004468)
Supplement: Attachment — Submitted filename: A Response Letter to Reviewers 2nd round.docx [file pmed.1004468.s010.docx]

**A point-by-Point Response for Reviewers 2^nd^ Round**

Title: Effect of Midwife-Led Pelvic Floor Muscle Training on Prolapse Symptoms and Quality of Life in Women with Pelvic Organ Prolapse in Ethiopia: A Cluster-Randomized Controlled Trial

Dear Editor,

We sincerely appreciate PloS Medicine for granting us the opportunity to receive expert feedback and enhance our manuscript. Our heartfelt thanks go to the editor and the diligent reviewers who dedicated their time to assess our work. For a point-by-point response and reviewer comments, please see the table below.

| **Reviewer Comments** | **Author Responses** | **Amendment** |
| --- | --- | --- |
| **Reviewer#1** |  |  |
| The authors have done a reasonable job of responding to the reviewers' comments, but there are still some areas that are not very clear. Specific comments follow: 1.      The information that the authors have added to the abstract is useful, but I'm not sure it really needs to be in the abstract (it's a bit distracting). Personally, I think it would be ok to just have it in the main text | Dear reviewer, thank you very much for your critical review and comments.  The editor recommended to organize the abstract according to CONSORT for abstract (CONSORT for Abstracts: PLoS Med and Lancet, January 2008) [1]. As a result, the current abstract section is more detailed than the original version. |  |
| 2. In the author summary, I suggest including the number of clusters in the first bullet point of the "What did the researchers do and find" section. | Thank you again.  We added this information that eight cluster (four per arm) were included in the study | Line 68 |
| 3.      I still find the paragraph "Each cluster in the study area is served by its own health posts, staffed by health extension workers, while five or more clusters are supported by a single health center. These health centers are staffed by formally trained healthcare professionals, including midwives. The current intervention was implemented at eight different health centers due to their capacity to deliver the training and their geographical accessibility to participants, especially when compared to hospitals." Confusing. It reads as if five of the clusters share the same health centre, but I wonder if it is supposed to mean that there are five cluster (or more?) that are each supported by a health centre, i.e. with different health centres for each of these clusters? I think that what might be causing the confusion is using the term "cluster" (specific to the trial) when actually they mean "kebele" (more general term)? | Thank you very much  Now we described it in short to avoid confusion. In Ethiopia, kebele (a small administrative unit) is served by its own health post, staffed by health extension workers. Groups of five or more kebeles are supported by a single health center, staffed by formally trained healthcare professionals, including midwives. For this study, the intervention was implemented at eight distinct health centers. The training and counselling were provided at health center b/c health centers can do it and are geographically accessible than hospitals. | Sampling procedure  Line 233-37 |
| 4.      In their response to one of my previous comments, the authors state that "We assigned different data collectors for the intervention and control sites, with two data collectors dedicated to each arm. This separation was intended to minimize any potential bias or contamination during data collection." It is true that this should minimise contamination, but then it could introduce more bias if the data collectors in one arm do things systematically differently to data collectors in the other arm - the authors could comment on this. | Thank you for pointing out the potential risk of data collector bias.  To minimize this concern, we ensured that all data collectors were trained, and used a standardized protocol to collect data in a uniform manner. The training emphasized adherence to the study procedures, the use of the same data collection tools, and unbiased interaction with participants. They were also masked about the participant allocation. Additionally, regular supervision and cross-checking of data were conducted.  These efforts were done to minimize the risk but can not totally avoid it. |  |
| 5.      In their response letter, the authors state that they have used the cluster-level analysis approach (i.e. described in Hayes and Moulton) but there does not seem to be a change to the statistical methods section to reflect this? Also I do not see these results in the tables? (It would be fine to just have them as supplementary tables). | Thank you very much for this insightful comment    Now we have supplemented it with cluster-level analysis (Supplementary file 5).    Dear reviewer, our primary aim was to do individual level analysis by considering the clustered nature of the data. We retained the cluster variable in our mixed-effects generalized linear model and sample selection weight were considered in all analyses. After receiving reviewer’s comments that emphasized on the limited number of clusters, we supplemented the analysis with cluster-level analysis (Supplementary file 5). We stated this approach in the method section as follows:  An individual-level analysis supplemented with cluster-level analysis were done to evaluate the effect of pelvic floor muscle training (PFMT) on prolapse symptoms. Individual-level analysis was performed using a mixed-effects generalized linear model (MEGLM), incorporating random effects to account for clustering by health centers and survey weights to adjust for unequal selection probabilities.  To complement the primary analysis, a cluster-level analysis was conducted following Hayes and Moulton’s framework. Cluster-level means were calculated for key outcomes (mean change in POP-SS, physical domain, psychological domain and personal relationship) and analyzed using linear regression to compare intervention and control clusters. | Statistical Analysis line 368-74 |
| 6.      With the newly edited sentence "Fortunately, midwives are generally more accessible than specialists, who are potentially capable of learning how to deliver PFMT service" it sounds like it's the specialists who are potentially capable. Suggest re-word to make it clear that the second part of the sentence is talking about the midwives. | Thank you for pointing out this.  We have revised the sentence for clarity as " Fortunately, midwives, who are generally more accessible than specialists, are capable of learning how to deliver PFMT services and are well-positioned to provide this support to women in their communities” | Background  line  125-27 |
| 7.      In the last sentence of the background, suggest the authors replace the word "significantly" by "will". | Thank you  We replaced it | Line 133 |
| 8.      The argument in the discussion that "The age discrepancy may stem from inaccuracies in measuring age, as many individuals in rural Ethiopia has no birth certificates. " isn't very clear - as it would also require that measurement error around age was different in one arm compared to the other trial arm? | Thank you very much again  The underlining cause is most probably lack of birth certificate that affect accurate measurement. Secondly, in estimating their age, different factors may affect accurate estimation based on their level of education, culture, and probing differently for age information. |  |
| **Reviewer#2** |  |  |
| My suggestions have been addressed well and I agree with the revised version. I like the publication as a significant enhancement of scientific knowledge on POP in Ethiopia. | Thank you very much  Your comments and suggestions were very important |  |
| **Reviewer#2** |  |  |
| I still have some residual concerns as follows:   1. Table 1 – numbers in the clusters don’t add up to the number of participants in the 2 districts in the rows above | Thank you for your feedback.    We apologize for the discrepancy. *Aposto* cluster actually contained 21 participants, not 26 as previously reported | Table 1 |
| 2. Reference 34 relates to the PROPEL study not the POPPY trial – line 272 | Thank you very much for this critical observation  We updated the citation in the revised version | Line 279 |
| 3. Don’t really understand why they translated the POP-SS from English to Sidaamu Afoo when an Amharic version exists and they translated the P-Qol from Amharic to Sidaamu Afoo. The 2 different approaches are hard to justify. | Thank you for the valuable question and we appreciate the opportunity to clarify our approach.  We opted to translate the POP-SS directly from the original English version to ensure that we captured the nuances and specific meanings intended by the developers of the questionnaire. This approach minimizes potential discrepancies that could arise from intermediary translations, such as those from English to Amharic and then from Amharic to Sidaamu Afoo. Prior to our study, we conducted a validation process for the translated POP-SS into Sidaamu Afo, which involved linguistic, cultural adaptation and psychometric measurements [2]. This validation ensures that the translated version is not only linguistically correct but also culturally relevant and comprehensible to our target population.  In contrast, due to time and resource constraints, we were unable to translate and validate the HRQoL tool from its original version into Sidaamu Afoo. Given these limitations, we opted to use the existing Amharic version. We assumed that the Amharic version would be relatively similar in context to our study population, allowing us to use it as a practical alternative. While this decision may not have undergone the same rigorous validation process as the POP-SS translation, it was made with consideration of available resources and timelines |  |
| Should add “n”s to table 2 as well as mean and SD. | Thank you again  We included the cluster size; and mean with SD | Table 2 |
| I am still not sure why they used a Bonferroni correction, and why for the primary outcome  measure analyses but not others. | Thank you for your question  Dear Reviewer, The Bonferroni correction was specifically applied to the primary outcome measure analyses to control for the risk of Type I errors across multiple comparisons. In clinical trials, the primary outcome is typically of most importance, as it drives the main conclusions of the study. By using the Bonferroni correction, we aimed to maintain a stringent significance level across these tests, thereby reducing the likelihood of false positives, which is crucial for accurately interpreting the primary outcomes.  In contrast, we chose not to apply the Bonferroni correction to secondary outcomes. These outcomes are generally intended to generate hypotheses rather than confirm causal relationships. By not applying Bonferroni in these cases, we preserved greater statistical power and recognized that the findings are not definitive. This approach minimizes the risk of overly stringent criteria that could lead to missing significant results [3]. |  |
| Re interactions and subgroups – text still not clear about whether interaction terms were  included in the models to look for subgroups effects. If yes, then why the need for a  stratified analysis? Text says: To control for effect modifiers, we employed statistical tests to assess interactions between PFMT and potential effect modifiers. This approach allowed us to determine whether the effect of PFMT varies across different subgroups. Additionally, we conducted stratified analyses to examine outcomes within specific strata, such as age and severity of prolapse. | Thank you for your insightful comment regarding the clarity of our analysis.  To clarify, we did include interaction terms in our models to assess potential subgroup effects of PFMT. However, the interaction terms did not yield significant results, indicating that the effect of PFMT did not vary meaningfully across these subgroups. As a result, we decided to exclude these interaction terms from the final model to maintain simplicity and focus on the main effects.  To avoid confusion in our manuscript, we have removed the second sentence from the revised version to clarify our methodology. We appreciate your feedback and believe these adjustments will enhance the clarity of our findings. |  |
| **General editorial requests: (Note: not all will apply to your paper, but please check each item carefully)** | | |
| We ask every co-author listed on the manuscript to fill in a contributing author statement, making sure to declare all competing interests…… | Thank you  We had already done it |  |
| Please upload any figures associated with your paper as individual TIF or EPS files with 300dpi resolution at resubmission; please read our figure guidelines for more information on our requirements: <http://journals.plos.org/plosmedicine/s/figures>. While revising your submission, please upload your figure files to the PACE digital diagnostic tool, <https://pacev2.apexcovantage.com/>. PACE helps ensure that figures meet PLOS requirements. To use PACE, you must first register as a user. Then, login and navigate to the UPLOAD tab, where you will find detailed instructions on how to use the tool. If you encounter any issues or have any questions when using PACE, please email us at [PLOSMedicine@plos.org](mailto:PLOSMedicine@plos.org) | Thank you  We did it accordingly | Figure 1 |
| Please ensure that the paper adheres to the PLOS Data Availability Policy (see <http://journals.plos.org/plosmedicine/s/data-availability>), which requires that all data underlying the study's findings be provided in a repository or as Supporting Information. For data residing with a third party, authors are required to provide instructions with contact information (web or email address) for obtaining the data. Please note that a study author cannot be the contact person for the data. PLOS journals do not allow statements supported by "data not shown" or "unpublished results." For such statements, authors must provide supporting data or cite public sources that include it. | Thank you  We provided the Stata data file as Supplementary file 2 |  |
| *       We expect all researchers with submissions to PLOS in which author-generated code underpins the findings in the manuscript to make all author-generated code available without restrictions upon publication of the work. In cases where code is central to the manuscript, we may require the code to be made available as a condition of publication. Authors are responsible for ensuring that the code is reusable and well documented. Please make any custom code available, either as part of your data deposition or as a supplementary file. Please add a sentence to your data availability statement regarding any code used in the study, e.g. "The code used in the analysis is available from Github [4] and archived in Zenodo [DOI link]" Please review our guidelines at <https://journals.plos.org/plosmedicine/s/materials-software-and-code-sharing> and ensure that your code is shared in a way that follows best practice and facilitates reproducibility and reuse. Because Github depositions can be readily changed or deleted, we encourage you to make a permanent DOI'd copy (e.g. in Zenodo) and provide the URL. | Thank you  We have no personal codes to declare |  |
| FORMATTING - GENERAL *       Abstract: Please structure your abstract using the PLOS Medicine headings (Background, Methods and Findings, Conclusions). Please combine the Methods and Findings sections into one section. | Thank you  We restructured it | Abstract |
| At this stage, we ask that you include a short, non-technical Author Summary of your research to make findings accessible to a wide audience that includes both scientists and non-scientists. The Author Summary should immediately follow the Abstract in your revised manuscript. This text is subject to editorial change and should be distinct from the scientific abstract. Ideally each sub-heading should contain 2-3 single sentence, concise bullet points containing the most salient points from your study. In the final bullet point of 'What Do These Findings Mean?', please include the main limitations of the study in non-technical language. Please see our author guidelines for more information: <https://journals.plos.org/plosmedicine/s/revising-your-manuscript#loc-author-summary> | Thank you  We provide the Author summary next to the abstract section |  |
| Please express the main results with 95% CIs as well as p values. When reporting p values please report as p < 0.001 and where higher as the exact p value p=0.002, for example. Throughout, suggest reporting statistical information as follows to improve clarity for the reader "22% (95% CI [13%,28%]; p</=)". Please be sure to define all numerical values at first use. | Thank you  We did it with 99% CI because we adjusted the significance level for multiple comparison since we are measuring four outcomes from a single study. Failure to adjust this may introduce type I error [3]. |  |
| Please include page numbers and line numbers in the manuscript file. Use continuous line numbers (do not restart the numbering on each page). | Thank you  We did it |  |
| Please cite the reference numbers in square brackets. Citations should precede punctuation. | Thank you  We did it |  |
| FIGURES AND TABLES *       Please provide titles and legends for all figures and tables (including those in Supporting Information files). | Thank you  We did it |  |
| Please define all abbreviations used in each figure/table (including those in Supporting Information files). | Thank you  We did it |  |
| Please consider avoiding the use of red and green in order to make your figure more accessible to those with color blindness. | Thank you  We have no such colors in one Figure 1. |  |
| SUPPLEMENTARY MATERIAL *       Please note that supplementary material will be posted as supplied by the authors. Therefore, please amend it according to the relevant comments outlined here.  *       Please cite your Supporting Information as outlined here: <https://journals.plos.org/plosmedicine/s/supporting-information> | Thank you  We did it |  |
| REFERENCES *       PLOS uses the numbered citation (citation-sequence) method and first six authors, et al. | Thank you  We did it |  |
| Please ensure that journal name abbreviations match those found in the National Center for Biotechnology Information (NCBI) databases (<http://www.ncbi.nlm.nih.gov/nlmcatalog/journals>), and are appropriately formatted and capitalised. | Thank you  We checked it |  |
| Where website addresses are cited, please include the complete URL and specify the date of access (e.g. [accessed: 12/06/2023]). | Thank you  We checked for it |  |
| Please also see <https://journals.plos.org/plosmedicine/s/submission-guidelines#loc-references> for further details on reference formatting. | Thank you  We did it |  |
| RCTs *       PLOS Medicine requires that all trials be prospectively registered in one of registries recognized by WHO. Please ensure that study registration details are included in the Methods section. | Thank you  We indicated that the trial was registered at the Pan African Clinical Trial Registry ([https://pactr.samrc.ac.za](https://pactr.samrc.ac.za/)) database, with the registration number PACTR202302505126575 |  |
| *       Please structure the Methods section using the following sub-headings: Study design and participants, Randomization and masking, Procedures, Outcomes, Statistical analysis. | Thank you  We have restructured it |  |
| Exclusion criteria not detailed in manuscript (from protocol: Women who were diagnosed to have symptomatic Pelvic Organ Prolapse stage I-III but planned to leave the study area within six months (study period), severely ill women, women with psychiatry disorders, women with delivered myoma, or women who want to have prolapse surgery within six months will be excluded.) Please clarify and explain all discrepancies between the paper and protocol. If the outcomes were not prespecified in the protocol, please define them in the Methods (Outcomes section) as post hoc and explain why they were added. Post-hoc comparisons should be presented as hypothesis generating rather than conclusive. | Thank you  Now we detailed the exclusion criteria. However, we did not get women with these criteria practically, that was why we didn’t listed these previously.  The outcomes were prespecified in the protocol under subsection “Measurement” for POP-SS and P-QoL separately (the plan was to produce two different manuscripts but now we merged it). | Line 186-89 |
| *       Please ensure that all prespecified outcomes (primary, secondary, and exploratory) are listed in the Methods/Outcomes section and indicate whether there are outcomes that are not presented in the current report. | Thank you  We presented all the prespecified outcomes. |  |
| Please specify the dates (Month Day, Year) during which study enrollment and follow up occurred. | Thank you  The enrollment period was open for eight months from 13 March to 28 October 2023. Each participant was followed for six months after enrollment. The last participants were enrolled on October 28, 2023 so the final follow-up visit was on April 27, 2024. | Line 208-10 |
| *       Please include absolute numbers wherever you report percentages; eg, n/N (%) | Thank you  We included absolute numbers for all percentage indicated. |  |
| Please present the safety data for the study including numbers of specific events and whether or not adverse events are thought to be related to treatment. AEs should be reported in the abstract, per CONSORT and CONSORT-Harms. | Thank you  We reported it in the CONSORT and main document |  |
| *       Please complete the CONSORT checklist (<https://www.equator-network.org/reporting-guidelines/consort/>) and ensure that all components of CONSORT are present in the manuscript, including how randomization was performed, allocation concealment, blinding of intervention, definition of lost to follow-up, power statement. When completing the checklist, please use section and paragraph numbers, rather than page numbers. | Thank you  We did it and attached it as a supplementary file 1 |  |
| *       Please report your abstract according to CONSORT for abstracts, following the PLOS Medicine abstract structure (Background, Methods and Findings, Conclusions) <https://www.equator-network.org/reporting-guidelines/consort-abstracts/> | Thank you  We did it as supplementary file 4 |  |
| *       If your trial had to undergo important modifications in response to extenuating circumstances, please complete the CONSERVE-CONSORT checklist and provide in your Supporting Information; (<https://www.equator-network.org/reporting-guidelines/guidelines-for-reporting-trial-protocols-and-completed-trials-modified-due-to-the-covid-19-pandemic-and-other-extenuating-circumstances-the-conserve-2021-statement/>). When completing the checklist, please use section and paragraph numbers, rather than page numbers. | Thank you  Our trial has nothing to undergo important modifications in response to extenuating circumstances. |  |
| *       In keeping with our commitment to Open Science, please include the study protocol document and analysis plan (including any amendments) as Supporting Information to be published with the manuscript if accepted. | Thank you  We uploaded it as Supplementary file 3 |  |
| *       Please note that PLOS Medicine requires prospective, public registration of a data sharing plan (as part of mandatory clinical trials registration) for all clinical trials that began enrollment on or after January 1, 2019, in accordance with ICMJE requirements. | Thank you  We indicated that the trial was registered at the Pan African Clinical Trial Registry ([https://pactr.samrc.ac.za](https://pactr.samrc.ac.za/)) database, with the registration number PACTR202302505126575.  Again we have attached the Stata data file with this manuscript as supplementary file 2. |  |

1.	Hopewell S, Clarke M, Moher D, Wager E, Middleton P, Altman DG, et al. CONSORT for reporting randomised trials in journal and conference abstracts. The Lancet. 2008;371(9609):281-3.

2.	Siyoum M, Teklesilasie W, Nardos R, Sirak B, Astatkie A. Reliability and validity of the Sidaamu Afoo version of the pelvic organ prolapse symptom score questionnaire. BMC Women's Health. 2023;23(1):324.

3.	Bland JM, Altman DG. Multiple significance tests: the Bonferroni method. Bmj. 1995;310(6973):170.

4.	Roth SM, Ivey FM, Martel GF, Lemmer JT, Hurlbut DE, Siegel EL, et al. Muscle size responses to strength training in young and older men and women. Journal of the American Geriatrics Society. 2001;49(11):1428-33.
